# Impacts of Puppy Early Life Experiences, Puppy-Purchasing Practices, and Owner Characteristics on Owner-Reported Problem Behaviours in a UK Pandemic Puppies Cohort at 21 Months of Age

**DOI:** 10.3390/ani14020336

**Published:** 2024-01-22

**Authors:** Claire L. Brand, Dan G. O’Neill, Zoe Belshaw, Fiona C. Dale, Bree L. Merritt, Kathryn N. Clover, Mi-Xue Michelle Tay, Camilla L. Pegram, Rowena M. A. Packer

**Affiliations:** 1 Department of Clinical Science and Services, The Royal Veterinary College, Hawkshead Lane, North Mymms, Hatfield, Herts AL9 7TA, UK; clbrand@rvc.ac.uk (C.L.B.); bmerritt@rvc.ac.uk (B.L.M.);; 2 Department of Pathobiology and Population Sciences, The Royal Veterinary College, Hawkshead Lane, North Mymms, Hatfield, Herts AL9 7TA, UK; doneill@rvc.ac.uk (D.G.O.); fidale@rvc.ac.uk (F.C.D.); cpegram@rvc.ac.uk (C.L.P.); 3 EviVet Evidence-Based Veterinary Consultancy, Nottingham NG2 5HY, UK; z.belshaw.97@cantab.net

**Keywords:** dogs, puppy, COVID-19, pandemic, welfare, behaviour, training

## Abstract

**Simple Summary:**

Problem behaviours are a leading cause of relinquishment and euthanasia of young dogs. Previous research has identified associations between owner-reported problem behaviours and risk factors, including how dogs were acquired as puppies, early socialisation experiences, and owners’ experience levels. Puppies acquired during the UK COVID-19 pandemic (“Pandemic Puppies”) were exposed to many of these risk factors; however, consequences for their adult behaviours are, as yet, unexplored. This study aimed to explore the impact of these early-life risk factors, in addition to owner management strategies (including training methods), upon owner-reported problem behaviours in a UK cohort of *n* = 985 Pandemic Puppies aged 21 months. Overall, 96.7% of owners reported their dog exhibited at least one problem behaviour by this age, and almost one third had displayed separation-related behaviours. Owners reporting more problem behaviours were more likely to use aversive training techniques (e.g., physical punishment), the use of which was high in this population (82.3%). Almost one third of owners had underestimated how hard training their dog would be; this view was more likely among first-time owners. Urgent efforts are required to support this vulnerable population of dogs, including providing owner education regarding humane training techniques, to improve their emotional health and avoid future relinquishment and/or behavioural euthanasia.

**Abstract:**

Problem behaviours are a leading cause of relinquishment and euthanasia of young dogs. Previous research has identified associations between owner-reported problem behaviours and risk factors, including how dogs were acquired as puppies, early socialisation experiences, and owners’ experience levels. Puppies acquired during the 2020 phase of the UK COVID-19 pandemic (“Pandemic Puppies”) were more likely to be exposed to many of these risk factors compared to puppies acquired in 2019; however, consequences for their adult behaviour are, as yet, unexplored. This study aimed to investigate the impact of these early-life and provenance-based risk factors, in addition to owner management strategies (including training methods) into early adulthood, upon adult dog behaviour aged 21 months. An online longitudinal cohort study of *n* = 985 Pandemic Puppies was conducted, recruited from a cohort of *n* = 4369 puppies originally surveyed in November–December 2020, which included data on how and why they were acquired and their socialisation/habituation experiences <16 weeks of age. Nearly all owners (96.7%) reported that their dog had exhibited at least one problem behaviour up to 21 months of age (median: 5; IQR: 3–7). Almost one third of dogs (30.9%) were reported to have displayed separation-related behaviours. Multivariable linear regression modelling revealed that owners reporting more problem behaviours were more likely to use multiple aversive training techniques (e.g., physical punishment), the use of which was notably high in this population (82.3%) compared to previous studies. Attendance at online puppy classes was the sole protective factor against owner use of aversive training methods. Almost one third of owners had underestimated how hard training their dog would be; this view was more likely among first-time owners. Urgent efforts are required to support this vulnerable population of dogs, including providing owner education regarding humane training techniques, to improve canine emotional health and avoid future relinquishment and/or behavioural euthanasia.

## 1. Introduction

Behaviours in dogs that owners find problematic are a major welfare challenge, not just for the affected dogs and the negative emotional states they may reflect [1], but also for their caregivers through “caregiver burden” (negative psychological and physiological outcomes) [2,3]. Owners of dogs showing anxiety-based behaviours including fear/avoidance and separation-related behaviours (SRBs) may resort to aversive training methods [4,5], and these dogs are at an increased risk of relinquishment [6] and euthanasia due to behaviour problems, particularly while the dogs are still young [7]. A recent UK study reported the prevalence of owner-reported problem behaviours in dogs at 76% [4]. However, many dog behaviours that owners consider problematic may be normal species-specific behaviours which conflict with human preferences or expectations (e.g., barking, motivation to chase/hunt for prey, chewing) [8]. Given the scale of these owner-reported problem behaviours and their potential negative welfare outcomes, gaining a deeper understanding of their risk factors is of great importance as this may promote enhanced prevention or mitigation in the future.

Previous risk factor studies have identified potential contributors to common owner-reported problem behaviours including limited socialisation experiences between 7 and 16 weeks of age [9,10,11], use of aversive training methods by owners [4,12,13], and first-time dog ownership [14,15,16]. However, few studies have yet explored the impact of the provenance of puppies on their later adult-dog behaviour. This is despite the sensitive period for socialisation in puppies beginning between the age of 3 and 5 weeks, which is before puppies can be legally sold in the UK [17,18]. The evidence that does exist suggests that poor provenance can have negative long-term impacts on dog behaviour, with puppies purchased via pet shops (which can serve as a proxy for potentially illegal third-party sales) [9,19,20,21] or via puppy farms [22] reported to exhibit increased levels of problem behaviours as adults compared to dogs purchased directly from breeders. Furthermore, acquisition under 8 weeks of age and puppies being purchased without their new owners seeing their dam and sire at the time of purchase have been associated with increased problem behaviours in later life [23,24].

The COVID-19 pandemic led to an international surge in puppy acquisitions, dubbed the “Pandemic Puppy” phenomenon [25,26]. A large UK-based cohort study of Pandemic Puppies acquired before 16 weeks of age following the first UK “lockdown” (between 23 March and 31 December 2020; *n* = 4369) identified that these puppies were significantly more likely to be exposed to several poor purchasing practices that have previously been linked to the development of owner-reported problem behaviours compared to puppies acquired at the same age pre-pandemic in 2019. This included owners being significantly less likely to have adhered to recommended purchasing behaviours, by, for example, being less likely to see their puppies’ mother at collection, despite this being a UK legal requirement for breeders [27]. In addition, purchasers of Pandemic Puppies were more likely to follow practices that reduced the transparency of purchase provenance, e.g., they were less likely to view their puppy in person prior to acquisition, more likely to collect their puppy outside the seller’s home, and more likely to have purchased a puppy with a passport (suggesting the puppy had been imported into the UK) [25,28]. In addition to provenance-based factors, Pandemic Puppies were significantly less likely to be exposed to some key socialisation experiences before 16 weeks of age, including attending puppy classes or experiencing visitors to their homes [28]. The risk of owners reporting behavioural problems in the future was also potentially elevated by owner experience-based risk factors, such as Pandemic Puppies being more likely to be acquired by first-time owners.

In addition to increased prevalence of poor purchasing practices by owners during the pandemic, changes in the motivations of owners for why they acquired a puppy during the pandemic may further predispose dogs to later behavioural problems. Two out of five owners of Pandemic Puppies reported that their decision to acquire a dog was motivated by the pandemic, most commonly (86.7%) from perceptions of having more time to care for a dog [25]. However, given that post-pandemic lifestyles are now likely to be very different to those that existed at the time of puppy purchase during the pandemic for many households (e.g., adults returning to working in office environments, children returning to study at school) [29], combined with evidence that temporary reductions in time dogs are left alone increase the risk of SRBs [30], major concerns for development of later behavioural problems in this Pandemic Puppies population have been raised [31]. Furthermore, perceptions of acceptable breed-based behaviours may bias owner perspectives about what is “normal” versus abnormal dog behaviour, skewing expectations among owners for how they expect their dogs should behave. For example, owners of Pandemic Puppies were significantly more likely than pre-pandemic owners to base their choice of breed on a perception as “easy to train”, even though the evidence for breed-specific differentials on ease of training is scant [25].

The Pandemic Puppies cohort thus provides a unique population and resource to examine a wide range of risk factors for the development of owner-reported problem behaviours in a predisposed group of dogs, as well as also exploring how owners manage and perceive these behaviours. Although there has been much speculation in the media and veterinary community on how puppy acquisition during the pandemic would negatively affect behaviour in this population of dogs as they aged [31], remarkably little research has been published to date that empirically reports on these issues. To the authors’ knowledge, only one small Italian study, using a cross-sectional online survey, has explored this issue [32]. The Italian study reported that dogs acquired from breeders or rehoming centres as puppies up to the age of five months between March and May 2020 (*n* = 173) were significantly more likely to display non-social fear, fear of handling, aggression towards other dogs, and general aggression aged under 34 months than dogs acquired as puppies at the same age and in the same manner between June 2020 and February 2021 (*n* = 137) [32]. To fill this information gap on how puppy acquisition and raising during the pandemic would impact dog behavioural development, the current study, for the first time, aimed to longitudinally follow a cohort of Pandemic Puppies purchased from breeders under the age of 16 weeks in the UK as these puppies reached 21 months of age. The study specifically sought to characterise and identify risk factors for:

1. Owner-reported problem behaviours, including separation-related behaviour problems;
2. Owner use of aversive training methods;
3. Owner expectations vs. realities of their dog’s behaviour and training;
4. Owners seeking professional advice for the behaviour and training of their dog.

Data from the 21-month survey were selected for analysis as part of the ongoing longitudinal “Pandemic Puppies” study, as this is the first adult “catch up” time point available for analysis following the initial survey of puppies aged under 16 weeks. As a wider longitudinal study, later timepoints in this cohort of dogs’ lives will be investigated for the same and broader outcomes in the future.

## 2. Materials and Methods

### 2.1. Ethical Statement

The Social Science Research Ethical Review Board at the Royal Veterinary College (RVC; URN: SR2020-0259) granted ethical approval for this study.

### 2.2. Survey Design and Content

An online questionnaire was designed iteratively amongst the authors and piloted on a small number of participants to ensure understanding and ease of completion. The questionnaire covered four sections related to the current analysis of problem behaviours: (i) ownership status, e.g., whether the respondent still owned their dog and, if not, the circumstances of their dog’s relinquishment or death; (ii) the dogs behaviour aged up to 21 months, e.g., owner-reported behaviour problems, SRBs, and sources of advice on dog behaviour; (iii) dog training and management, e.g., attendance at adult dog training classes, use of training methods, and changes in the periods when the dog is left alone compared to when aged <16 weeks; and (iv) owner expectations and experiences of canine behaviour and training, and consideration of relinquishment.

This online questionnaire was part of a larger longitudinal study of outcomes among puppies acquired aged <16 weeks during the 2020 phase of the COVID-19 pandemic. The full longitudinal questionnaire explored broad areas including dog–owner relationships, health outcomes and provisions, and the continued impact of the COVID-19 pandemic on the owner’s life. However, these other data were not considered in this current analysis. The survey questions relevant to this study can be found in Supplementary Materials File S1.

Data relating to the owners’ demographics (e.g., age, gender, geographical location, previous dog ownership experience), along with details of their pre-purchase, purchase, and post-purchase motivations and behaviours while acquiring their puppy were available from the original 2020 RVC Pandemic Puppies survey, from which this longitudinal cohort was recruited [25]. Dog demographic data (e.g., breed/crossbreed, date of birth), age at acquisition, and socialisation experiences <16 weeks of age were also available from this earlier dataset [28]. Variables that were liable to change over time, notably neuter status and attendance at puppy classes <16 weeks of age (i.e., for owners who completed the original study while their dog was <16 weeks), were re-collected at 21 months to increase the accuracy of the current analysis dataset.

### 2.3. Participant Recruitment

The 21-month questionnaire was hosted using a version of the Vanderbilt University’s Research Electronic Data Capture platform (REDCap) provided by the RVC, a part of the REDCap Consortium [33,34]. Owners were recruited from the original 2020 Pandemic Puppies study population, for which they were required to: (i) be over 18 years of age; (ii) be resident in the UK; (iii) have brought a dog of any breed or crossbreed home <16 weeks of age between 23 March and 31 December 2020, or the same date range in 2019; and (iv) have purchased their dog rather having a dog that had been rehomed or bred by themselves.

From this original population, a subset of owners was recruited for the current longitudinal cohort study. These owners had (v) brought their dog home between 23 March and 31 December 2020 (i.e., the dogs were “Pandemic Puppies”); (vi) indicated they were happy to be contacted for inclusion in further research; and (vii) provided a valid email address for contact.

Owners fulfilling these inclusion criteria were invited to complete the questionnaire as their dogs reached 21 months of age. A unique questionnaire link was emailed via REDCap beginning 24 January 2022 directly to each owner 14 days prior to their dog turning 21 months (639 days) of age, based upon the date of birth the owner had provided in the 2020 RVC Pandemic Puppies survey. This unique link elapsed after 28 days, with up to two reminder emails sent to each owner if they had not started or completed the survey after 14 and 26 days.

#### Informed Consent and Opt-Out

**Figure 1:**
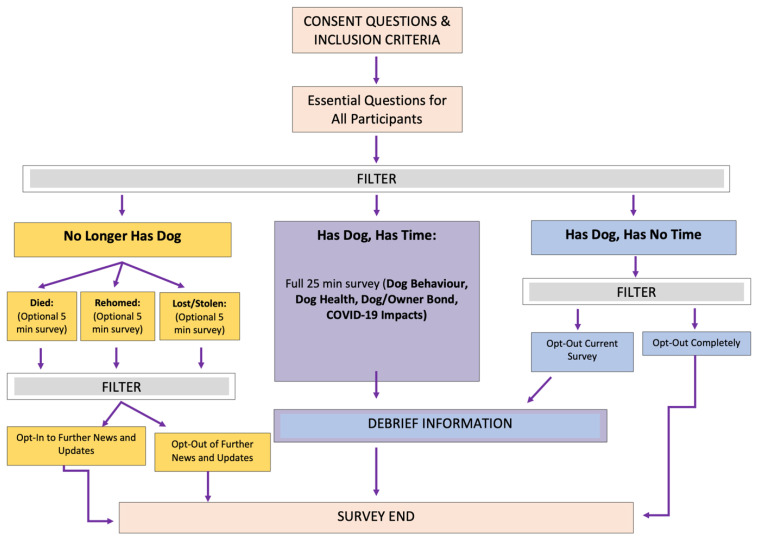
Schematic representation of the 21-month survey structure used for owners of UK Pandemic Puppies acquired <16 weeks of age between 1 July and 31 December 2020. Branching logic and filter questions gave owners a personalised survey experience. A brief survey option was available for owners who no longer had their dogs, including those dogs that had been rehomed or died, to encourage participation. An option to opt-out of the current survey and/or all future surveys was also provided. Owners indicating they were happy to take part were directed to the full survey.

All owners gave informed consent at the start of the questionnaire and had the option to opt out following the first question regarding their dog’s current ownership status. Using survey logic, owners who no longer owned their dog were directed to an optional five-minute questionnaire exploring the circumstances surrounding why they no longer owned their dog (Figure 1).

Owners were unable to go back and amend answers to previous questions to mitigate social desirability bias in the light of new information presented in follow-on questions.

### 2.4. Data Collection, Categorisation, and Cleaning

The raw survey data were exported from REDCap into Microsoft Excel for Mac (version 16.79.2) for cleaning prior to analysis. The status of variables liable to have changed between the 2020 RVC Pandemic Puppies survey and 21-month survey was checked and updated as required (see Table S1, Supplementary Materials File S2).

#### 2.4.1. Owner-Reported Problem Behaviours

Owners were asked whether, since the original survey in November/December 2020, they considered their dog had a problem with any behaviours from a list of 24 behaviours derived from the relevant scientific literature (Table 1), and if so, whether they had sought formal or informal advice regarding the problem behaviour (Question 39a). A free text option was also provided for owners to describe additional problem behaviours not on the list (Question 39b). To minimise multiple testing, the individual owner-reported problem behaviours were grouped and analysed in two ways:

1. Total number of owner-reported problem behaviours (out of 24, i.e., not including any additional behaviours derived from analysis of the free text).
2. The presence or absence of seven groupings of different types of behaviour, based upon previous categorisations from the literature [4] and including additional behaviours derived from analysis of the free text (see Table 1).

#### 2.4.2. Separation-Related Behaviours

Owners were asked two questions regarding SRBs up to 21 months of age, which were used to assign the dogs into an affected (case) or unaffected (control) group:

1. “Since the last survey (in November/December 2020), has your dog shown any of the following behaviours when at home with you/other household members while you are relaxing (e.g., watching TV)?”, with the response options “Yes” or “No” for each behaviour.
2. “To your knowledge (e.g., from seeing evidence upon your return home, on a video camera, being told by neighbours, etc.), since the last survey (in November/December 2020), has your dog shown any of the following behaviours when left at home alone (without any people present in the home)?” with response options “Yes”, “I suspect they might”, “No”, or “I don’t know”. For analysis purposes, these responses were condensed; “I suspect they might” was included in the “Yes” category and “I don’t know” was included in the “No” category.

These questions did not ask whether the behaviours were a “problem” (e.g., to either the owner or the dog), just whether they were displayed, as some of the behaviours (e.g., spinning) may not be reported as being problematic to the owner, but may reflect underlying problems with the emotional state of the dog [35].

Both questions offered options for nine commonly reported SRBs [36,37,38]: “Destructive behaviour (e.g., chewing and causing damage to household items such as furniture, not including their own toys/treats)”, “Scratches doors or around doors”, “Barks”, “Howls”, “Whines/cries”, “Wees in an inappropriate place, e.g., inside the home if usually housetrained”, “Poos in an inappropriate place, e.g., inside the home if usually housetrained”, “Spins (repeatedly turns in a circle)” and “Paces (walks repetitively around the home/room, unable to settle”.

The responses to these two questions were combined to categorise each dog based on the presence/absence of the nine behaviours:

1. SRB case: at least one behaviour was only displayed whilst the dog was left alone, the remaining behaviours could be displayed by dogs whilst relaxing in their owners’ presence, in both contexts (relaxing with owners or alone), or not displayed at all.
2. SRB control: no behaviour was displayed solely whilst the dog was left alone, all behaviours were instead either displayed by dogs whilst relaxing in their owners’ presence, in both contexts (relaxing with owners or alone), or not displayed at all.

#### 2.4.3. Owner Response to Separation-Related Behaviours

Owners who answered “Yes” to any of the nine SRB behaviours being displayed whilst the dog was left alone were further asked about their response to the behaviours soon after their return (question 53).

For analysis purposes, responses were categorised as no response (ignored what had happened), positive response (reassured or played with the dog), or negative response (used some form or verbal or physical punishment). Owners choosing options spanning both “Positive response” and “Negative response” were coded as “Mixed response”.

#### 2.4.4. Training Methods/Aids

Owners were presented with a list of commonly used training methods/aids derived from the literature [9,34] and asked, “Have you or anyone in your household ever used any of the following aids or methods on/with your dog, to try and change any aspect of their behaviour?”; a free text option was also provided. The list of methods/aids (Table 2) were categorised based on prior definitions [39] and categorisations [9]. Briefly, each training method/aid was allocated to the four quadrants of operant conditioning and categorised broadly as rewards-based (positive reinforcement/negative punishment) or aversive (negative reinforcement/positive punishment). The choice of “Harness” could fall under different quadrants dependent upon its design [40], and therefore was considered ambiguous and not categorised as rewards-based or aversive.

Owners who reported using only positive reinforcement or negative punishment aids/methods were then categorised as rewards-based-only trainers, while owners using only negative reinforcement or positive punishment were categorised as aversive trainers (Table 3). Those using a mix of rewards-based and aversive methods/aids were separated into two categories: those that used only one aversive method/aid alongside other rewards-based methods, and those that used multiple (two or more) aversive methods/aids alongside rewards-based methods [41] (Table 3). For analysis purposes, the owner training method variable was further collapsed into “rewards-based only”, “mixed reward-based/aversive” and “aversive-only”.

#### 2.4.5. Owner Expectations and Realities of Dog Behaviour and Training

Owners were asked two questions adapted from those published previously [42] to assess their expectations vs. realities of dog ownership with regard to training and behaviour. For training, owners were asked: “Compared to my expectations when I first acquired my dog, training and ongoing maintenance of their basic obedience since the last survey (in November/December 2020) has been…”, with the response options: “Harder than I expected”, “As I expected”, “Easier than I expected” or “I’m not sure/I can’t remember”. For behaviour, owners were asked to answer the question “Compared to my expectations when I first acquired my dog, their behaviour since the last survey (in November/December 2020) has been…”, with the response options: “Worse than I expected”, “As I expected”, “Better than I expected” or “I’m not sure/I can’t remember”. Both questions were supplemented with an option for owners to add free text to explain their response.

For further analysis, responses to the two expectations questions were categorised as a binary variable (1/0) where “Harder/worse than I expected” was coded as “1”, whilst “As I expected” and “Easier/better than I expected” were coded together as “0”. Responses of “I’m not sure/I can’t remember” were treated as missing data.

#### 2.4.6. Potential Indicators of Illegal Puppy Sale

Five variables from the original 2020 Pandemic Puppies study regarding how puppies were acquired [25,28] were used to indicate potentially illegal puppy sales, as a proxy for poor provenance and thus the risk of poor welfare prior to purchase (Table 4). The variables were turned into binary outcomes for the purpose of the current analysis, where any responses of “I’m not sure” or “I can’t/don’t remember”, etc., were treated as missing data and “Not applicable” was treated as “No”.

#### 2.4.7. Categorisation of Purebred Status

As described previously [28], each dog was categorised from its breed/crossbreed into four groups: purebred (i.e., those with ancestry of the same breed over several generations); designer crossbred (i.e., intentional crosses of different breeds/crossbreeds and given an indicative name of this cross, e.g., “Cockapoo” for a Cocker Spaniel crossed with a Poodle breed), crossbred (i.e., defined as mixed bred dogs of unknown origin, or crosses where only one generic aspect of their breed mix was reported, e.g., Spaniel cross), or no information (i.e., for those dogs with no breed information). Those dogs in the purebred group were further categorised into their respective breed grouping from The UK Kennel Club (KC; e.g., Toy, Gundog) [49], where applicable.

#### 2.4.8. Typical Adult Bodyweight Categorisation

Dogs breed/crossbreed was used to assign each dog a typical adult (>18 months of age) bodyweight (kg) using data collected on ≥100 dogs for each breed or crossbreed/sex combination [50,51], as previously described [28]. Bodyweight values were categorised as ≤10.0 kg, 10.0 to <20 kg, 20 to <30 kg, 30 to <40 kg, and ≥40 kg.

### 2.5. Quantitative Analysis

Following importation into IBM SPSS Statistics v29 (SPSS Inc., Chicago, IL., USA), descriptive statistics (frequency and percentage) were calculated for all variables. Distribution of continuous data was ascertained through visual inspection of histograms.

#### 2.5.1. Univariable Analysis

Demographic variables were compared individually between the 2020 RVC Pandemic Puppies survey owners and the subset of 21-month survey owners using chi-squared (*X*^2^) tests to explore whether and how this sub-population differed from the original study population.

Univariable and multivariable risk factor analysis was carried out for six outcome variables, namely:

1. Total number of owner-reported problem behaviours per dog (continuous);
2. SRB case/control (binary categorical);
3. Training methods used (binary categorical);
4. Owner expectations for behaviour (binary categorical);
5. Owner expectations for training (binary categorical);
6. Owner sources of advice for general behaviour and training (binary categorical).

The full list of risk factor variables assessed in these analyses is presented in full in Table S1, Supplementary Materials File S2; this includes owner/household demographics, dog demographics, purchase motivations and behaviours, potential indicators of illegal puppy sales, owner expectations, socialisation experiences <16 weeks of age, and ongoing behavioural management up to 21 months of age.

Univariable binary logistic regression was used for risk factor analysis for binary categorical outcome variables. The Mann–Whitney U test or Kruskal–Wallis test were used for univariable risk factor analysis of the non-normally distributed continuous outcome variable “total number of owner-reported problem behaviours per dog” and reported as median (IQR, range).

#### 2.5.2. Multivariable Analysis

Multivariable binary logistic regression modelling was used to identify risk factors for the binary categorical outcome variables. Multicollinearity was assessed iteratively by use of collinearity diagnostics and inspection of standard errors for inflation. Model building used a hybrid approach; variables that were liberally associated with the outcome (*p* ≤ 0.2) in univariable analyses were taken forward into multivariable modelling and their retention was assessed using automatic backwards elimination. In addition, some variables were identified a priori by the authors as being potential confounders for outcome associations, and were manually entered back into the final models regardless of their significance based on an “information theory” approach [52]. The quality of the model’s fit was evaluated using the Hosmer–Lemeshow test.

For the single continuous outcome variable (total number of owner-reported problem behaviours per dog), the above strategy was adopted to build a generalised linear model, with the quality of the model’s fit being evaluated using the adjusted R-squared approach.

The statistical significance for all models was set at the 5% level.

### 2.6. Qualitative Content Analysis

Qualitative content analysis of the free text options was performed as previously described [25]. Briefly, one author (C.L.B.) familiarised themselves with the data by reading all the free text responses. Where the free text responses were deemed to fit within the scope of existing, deductive fixed-choice responses, the data were back-allocated into that category if not already selected. One author (C.L.B) then developed a coding framework using an inductive approach based on the data for any unallocated responses or free text from questions with no existing fixed-choice responses; next, three authors (C.L.B., Z.B., and R.M.A.P.) agreed upon a single final set of codes, which were applied by one author (C.L.B.). For the purpose of the current study, the free text responses were analysed for seven questions with existing fixed-choice responses and four questions with no existing fixed-choice responses. The inductive and deductive coding frameworks for the questions analysed as part of this study can be found in Supplementary Materials File S3.

## 3. Results

A total of *n* = 1010 21-month survey responses were received from the *n* = 1742 owners contacted. Of these, three owners did not go beyond opening the survey, whilst one additional owner opted out of further research via email but did not indicate whether they still owned their dog. Valid responses from 1007 owners (58.4% of owners contacted) were therefore included in the current analysis.

### 3.1. Actual Relinquishment and Deaths by 21 Months of Age

Of the 1007 valid responses, 985 (97.8%) still owned their dog. Thirteen owners (1.3%) reported they had relinquished their dog and nine (0.9%) reported their dog had died. Analysis of free text provided by 17 of the *n* = 22 owners described above revealed that *n* = 4 dogs had been relinquished due to behavioural issues whilst *n* = 3 had been euthanised due to behavioural issues (Supplementary Materials File S3).

#### Considered Relinquishment by 21 Months of Age

Of the *n* = 877 owners who still owned their dog and answered the relevant question, 36 (4.1%) had considered relinquishing their dog since the initial survey in 2020 but were no longer considering doing so, whilst an additional *n* = 4 (0.5%) were still considering relinquishing their dog.

From 38 of these 40 owners who provided further information on why they considered relinquishment, 32/38 (84.2%) indicated it was due to their dog’s behaviour (Supplementary Materials File S3). From 31/40 owners who provided free text responses on what they thought may have helped/would help them to consider not relinquishing their dog, 20/31 (64.5%) would have valued post-purchase support, most commonly in the form of practical support from canine professionals, e.g., puppy training classes (*n* = 15; 48.4%). A further *n* = 8/31 (25.8%) would have valued pre-purchase advice, predominantly to improve their prior knowledge of dog ownership (*n* = 7; 22.6%) (Supplementary Materials File S3).

### 3.2. Demographics

Amongst owners who responded to the survey and indicated they still owned their dog (*n* = 985), 90.2% were female, with the largest proportion aged 45–54 years old (27.7%). In terms of ownership experience, 38.6% of owners had not previously owned a dog prior to purchasing their puppy. Analysis of household demographic data (*n* = 876 owners) indicated that *n* = 202 (23.1%) of owners worked exclusively from home, whilst *n* = 185 (21.1%) worked exclusively away from their home. An additional *n* = 268 (30.6%) of owners worked a combination of at home and away from their home, with the remaining proportion either unemployed (*n* = 30, 3.4%) or retired (*n* = 191, 21.8%). A total of *n* = 119 (13.6%, total *n* = 877) owners lived alone or were the only adult in their household.

In terms of breed, amongst the *n* = 985 responses these data were available for, the majority (68.5%) of dogs were purebred, with 26.8% categorised as a Designer Crossbreed. The five most common individual dog breeds/crossbreeds in the cohort were the Labrador Retriever (10.5%), Cockapoo (9.1%), Cocker Spaniel (7.1%), crossbreed (4.4%), and Border Collie (3.4%). Sex was evenly distributed, with just over half of the dogs being male (52.7%).

#### 3.2.1. Variables Collected in 2020 and Updated in the 21-Month Survey

Proportional neutering rose significantly between the original 2020 RVC Pandemic Puppies survey in December/November 2020 (190/984 19.3%) and the 21-month survey between January and August 2022 (*n* = 564/984, 68.9%; *X*^2^ = 498.98, *p* <0.001). Proportional pet insurance did not differ significantly between December/November 2020 (*n* = 813/930, 87.4%) and 21 months of age (*n* = 839/983, 85.4%; *X*^2^ = 1.70, *p* = 0.192).

A total of *n* = 208 (21.1%, *n* = 985) owners reported that they had not attended puppy classes in the original survey (November/December 2020) but that they intended to before their puppy was 16 weeks of age. At 21 months, 62.0% of owners (*n* = 129/208) reported that they had not attended puppy classes before their dog reached 16 weeks old, despite this intention, whilst the remaining 36.1% had attended puppy classes, either online (*n* = 23) or in-person (*n* = 52), since the last survey.

#### 3.2.2. Comparison of the 21-Month Cohort to That of the Original 2020 Pandemic Puppies Cohort

Dog and owner demographic data were compared between the *n* = 985 responses to the 21-month survey and the *n* = 4369 responses to the full original Pandemic Puppies cohort to assess the representativeness of the 21-month cohort (see Supplementary Materials File S4). Briefly, owners who responded to the 21-month survey were highly representative of the full cohort across 10 of the 13 variables examined, including dog ownership experience, owner gender, age at acquisition of puppies, and the most common breeds/crossbreeds. Three variables differed: owners in the 21-month survey were more likely to have been aged 55–≥75 in 2020; owners were less likely to have been employed in the animal care sector in 2020; and there was an increased proportion of Whippets compared to the original survey population (Supplementary Materials File S4).

### 3.3. Owner-Reported Problem Behaviours

Since the last survey, the three most common problem behaviours reported by owners were pulling on the lead (*n* = 601/892, 67.4%), jumping up at people (*n* = 507/890, 57.0%), and not coming back when called (*n* = 466/898, 51.9%) (Table 5).

**Figure 2:**
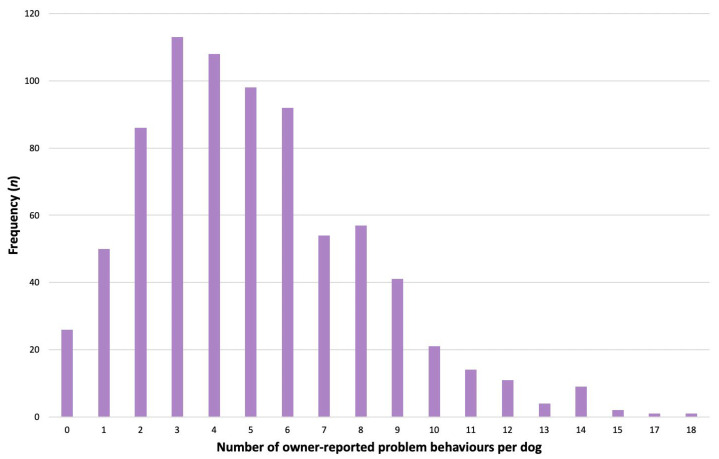
Distribution of the number of problem behaviours per dog since the last survey, reported by owners when their dog was 21 months of age, from a cohort of UK Pandemic Puppies acquired <16 weeks of age between 1 July and 31 December 2020 (*n* = 788).

From *n* = 788 owners who provided responses for all 24 problem behaviours, *n* = 762 (96.7%) reported that their dog displayed at least one problem behaviour. Among these *n* = 788 respondents, the median number of problem behaviours reported was 5.0 (IQR 3.0–7.0, range 0–18), with *n* = 161 (20.4%) owners reporting eight or more problem behaviours (Figure 2).

When categorised into behavioural problem groups as per Blackwell et al. (2008) [4], the most frequent behavioural problem group by 21 months of age was control behaviours (*n* = 742, 84.2%), followed by attention-seeking (*n* = 648, 77.3%) and fear/avoidance behaviours (*n* = 368, 41.4%) (Table 6).

#### Risk Factor Analysis for the Number of Owner-Reported Problem Behaviours

Twenty-two of the forty variables assessed using univariable linear regression for their association with the outcome measure “number of owner-reported problem behaviours per dog” were liberally associated (*p* < 0.2) (see Table S2, Supplementary Materials File S2). Following multivariable modelling, seven variables were retained in the final model in addition to the information theory variables (Table 7). The adjusted R-squared analysis indicated acceptable model fit (0.150, *p* <0.001).

The factors that were found to be significantly associated with dogs displaying a higher number of problem behaviours were the following: owner use of aversive methods/aids (compared to use of rewards-based methods/aids only); being walked off-lead once a week (compared to once per day); owners seeking advice from professional sources (compared to non-professional); being left alone for more time at 21 months than when <16 weeks of age (increased to over 4 h left alone, compared to no change in time left alone); dogs being male (compared to female).

Factors significantly associated with dogs displaying a lower number of problem behaviours were: hearing thunder whilst <16 weeks old (compared to those that did not); belonging to the KC Gundog group (compared to dogs from breeds/crossbreeds not KC-recognised); being an English Springer Spaniel (compared to a crossbreed); having owners employed in the animal care/veterinary sector (compared to those whose owners were not); being left home alone (compared to those never left alone).

### 3.4. Separation-Related Behaviours

**Figure 3:**
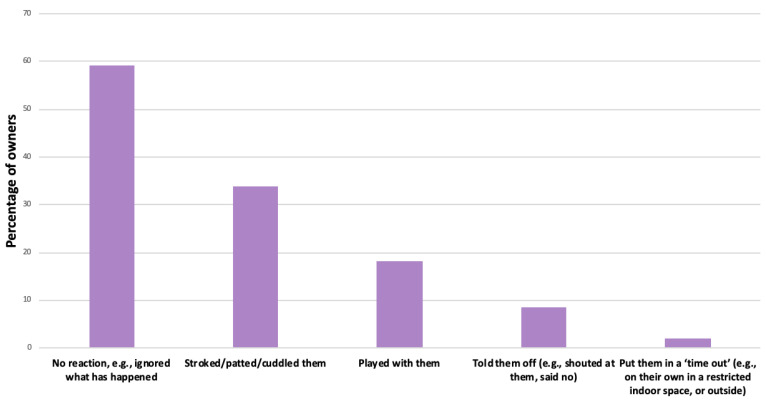
Owner response to their dogs’ separation-related behaviours upon returning to the home, as reported by their owners at 21 months old in a cohort of UK Pandemic Puppies acquired <16 weeks of age between 1 July and 31 December 2020 (*n* = 285).

Of those owners who responded to both SRB-related questions (*n* = 789), *n* = 244 dogs (30.9%) were categorised as SRB cases at the 21-month timepoint. When those owners (*n* = 285) of dogs who displayed any of the nine potential SRB-defining behaviours when left at home alone (whether they were later defined as an SRB case or not) reported their own behaviours upon returning home to their dog, *n* = 167/285 (59.2%) stated that they ignored the SRB-defining behaviour or any evidence of it. However, around one in ten owners (*n* = 26; 9.2%) reported that they either verbally or physically punished their dog upon their return when encountering SRBs or evidence of them (Figure 3).

#### 3.4.1. Length of Time and Location Dogs Were Left Alone at 21 Months

**Figure 4:**
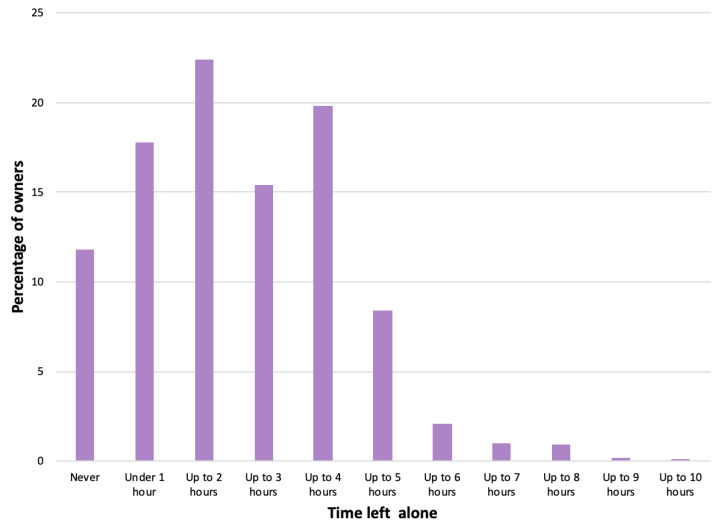
Distribution of the longest stretch of time dogs were left alone at 21 months of age in a cohort of UK Pandemic Puppies acquired <16 weeks of age between 1 July and 31 December 2020 (*n* = 897).

Owners were asked the longest length of time their dog was currently left home alone on a typical weekday at 21 months of age. Whilst *n* = 106 (11.8%) reported that they never left their dog alone, the majority of the *n* = 897 owners who responded left their dog alone for between one and four hours (*n* = 517, 57.6%), and *n* = 114 (12.7%) reported that they left their dog alone for over four hours at a time (Figure 4).

**Figure 5:**
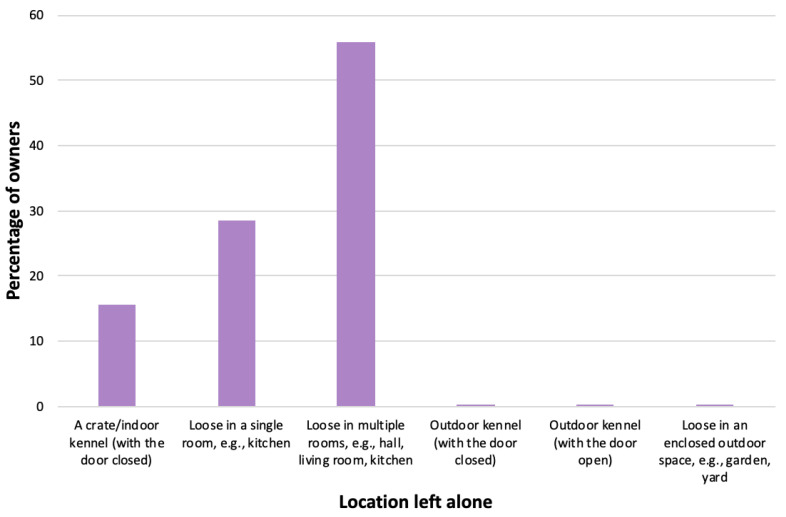
Location dogs were left alone at 21 months of age in a cohort of UK Pandemic Puppies acquired <16 weeks of age between 1 July and 31 December 2020 (*n* = 859).

With regard to the location(s) dogs left home alone could access during this time, the majority (*n* = 479, 55.8%) were left loose with access to multiple rooms, followed by loose in a single room (*n* = 244, 28.4%), or crated (*n* = 133, 15.5%), with only *n* = 3 (0.3%) owners indicating that their dogs were left outside the house (Figure 5).

#### 3.4.2. Comparative Time Dogs Were Left Alone between 2020 and 21-Month Surveys

**Figure 6:**
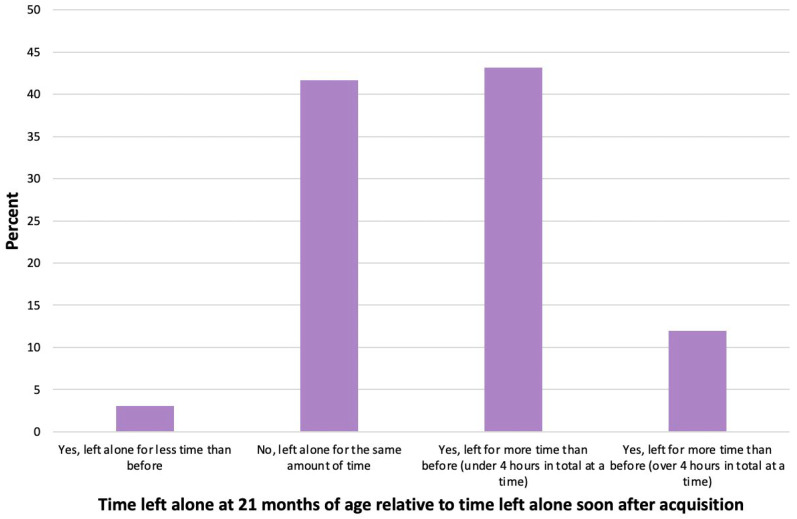
Change in time dogs were left alone between acquisition in 2020 (aged <16 weeks) and aged 21 months of age in a cohort of UK Pandemic Puppies acquired <16 weeks of age between 1 July and 31 December 2020 (*n* = 868).

Relative to the time dogs were left alone at or soon after acquisition ≤16 weeks, *n* = 479/868 owners (55.2%) indicated that they left their dog alone for longer periods of time at 21 months of age. These increased periods of time left alone at 21 months of age were most commonly for under 4 h in total (*n* = 375, 43.2%), but for over one in ten dogs this time increased to more than 4 h at a time (*n* = 104; 12.0%) (Figure 6).

#### 3.4.3. Risk Factor Analysis for Dogs Being Categorised as Separation-Related Behaviour Cases

Twenty-three of the forty-one variables assessed using univariable logistic regression for association with SRB cases were liberally associated (*p* <0.2) (see Table S3, Supplementary Materials File S2).

Four variables were identified via multivariable binary logistic regression modelling as being significantly associated with increased odds of dogs being categorised as an SRB case by 21 months of age: dogs displaying ≥1 attention-seeking behaviour or ≥1 fear/avoidance behaviour (compared to those that displayed none of these behaviour types); total number of owner-reported problem behaviours per dog; owners aged 25–34 years (compared to aged 45–54 years) (Table 8).

Three variables were significantly associated with reduced odds of being categorised as SRB cases by 21 months of age: typical adult body weight of 30 to <40 kg (compared to 10 to <20 kg); there being another dog in the household in 2020 when their dog was a puppy (compared to no other dog in the household in 2020); and being left alone >4 h at 21 months (compared to never being left alone) (Table 8).

A further four variables (general advice source for training and behaviour; aggressive behaviour score; control behaviour score, and reaction to unfamiliar people) were highlighted using automatic backwards elimination and retained in the final model to improve fit; however, these were not significant following the manual addition of the information theory variables. The Hosmer–Lemeshow test indicated an acceptable model fit (*p* = 0.604).

### 3.5. Training and Training Methods

#### 3.5.1. Attendance at Training Classes

At the 21-month time point, *n* = 392/984 owners (39.8%) reported that they had taken their dog to a puppy class whilst their dog was under the age of 16 weeks. Of these, the majority (*n* = 335, 85.5%) had attended in-person classes and *n* = 79 (20.2%) had participated in online classes. Adult dog training classes (>16 weeks of age) were attended by *n* = 415/927 (44.8%) of owners, of which *n* = 379 (40.9%) had participated in-person and *n* = 36 (3.9%) online.

#### 3.5.2. Training Methods Used in the First 21 Months of Ownership

**Figure 7:**
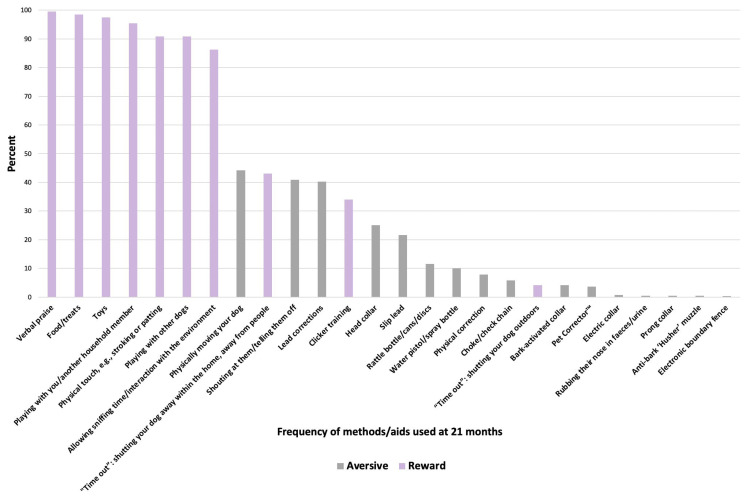
Frequency of different aversive and rewards-based training methods/aids used in the first 21 months of ownership in a cohort of UK Pandemic Puppies acquired <16 weeks of age between 1 July and 31 December 2020 (*n* = 758). N.B. Only data from owners who responded yes/no to all MCQ options presented (except harness, due to its ambiguity) are included.

The majority of owners (*n* = 754/758, 99.5%) reported using verbal praise as a training method with their dog. Six other rewards-based methods/aids were used by over 80.0% of owners, including food/treats, toys, and playing (Figure 7). The most commonly used aversive training method/aid was physically moving the dog (e.g., pushing on the dog’s hindquarters to get them into a sit, pushing them off furniture, or pushing them down if they jump up) (*n* = 335/758, 44.2%), followed by shouting at them/telling them off (*n* = 310/758, 40.9%) and lead corrections (*n* = 305/758, 40.2%) (Figure 7).

**Figure 8:**
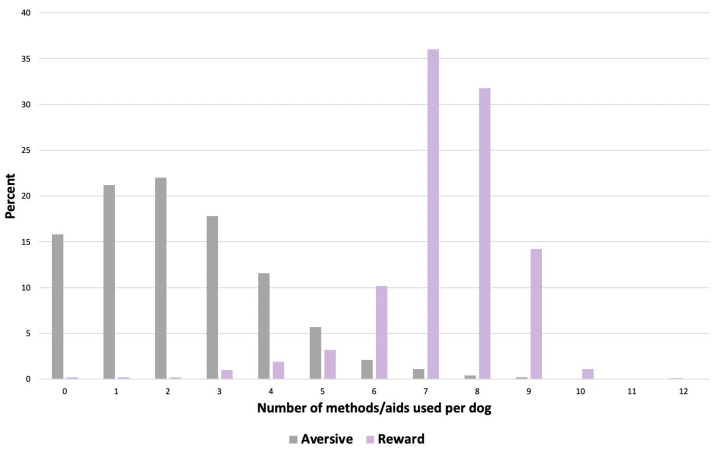
Distribution of the total number out of 26 potential options of different aversive (*n* = 898 total respondents) and rewards-based methods/aids (*n* = 925 total respondents) used per dog during the first 21 months of ownership in a cohort of UK Pandemic Puppies acquired <16 weeks of age between 1 July and 31 December 2020.

A minority of owners (*n* = 159/898, 17.7%) reported using no aversive training methods/aids, *n* = 190 (18.9%) reported using one aversive method/aid, *n* = 198 (22.0%) reported using two aversive methods/aids, and *n* = 351 (39.0%) reported using more than two aversive methods/aids (Figure 8). When considering the frequency of use of different rewards-based methods/aids, the largest proportion of owners (*n* = 333/925, 36.0%) reported using seven different methods/aids, followed by eight (*n* = 294/925, 31.8%), whilst a minority reported using five or less (*n* = 63/925, 6.8%) (Figure 8).

#### 3.5.3. Risk Factor Analysis for Owners Using One or More Aversive Training Method/Aid

Twenty-seven of the fifty variables assessed using univariable logistic regression were liberally associated (*p* <0.2) with the outcome measure “owner use of one or more aversive training method/aid(s)” (see Table S4, Supplementary Materials File S2).

The final multivariable model included 23 variables. Eleven variables were identified via multivariable binary logistic regression modelling as being significantly associated with increased odds of owners having used one or more aversive method/aid(s) during the first 21 months of ownership: dogs displaying ≥1 aggressive behaviour, ≥1 attention-seeking behaviour, ≥1 control behaviour, or ≥1 reaction to familiar people behaviour (compared to those that displayed none); total number of owner-reported problem behaviours per dog; dogs belonging to the KC Gundog or Pastoral group (compared to dogs of breeds/crossbreeds that were not KC-recognised); dogs having a typical adult body weight of 30 to <40 kg (compared to 10 to <20 kg); being left alone >4 h (compared to never being left alone); owners purchasing the breed/crossbreed because it was perceived as easy to train (compared to those without this perception) (Table 9).

Only attendance at online puppy classes <16 weeks of age was significantly associated with reduced odds of owners having used one or more aversive method/aid(s) up to their dog being 21 months of age (Table 9).

A further four variables (purchasing a dog to improve mental health; purchasing a breed/crossbreed because it was perceived to be good with children; the dog being sold as a puppy outside the breeder’s home; fear/avoidance behaviour score) were highlighted using automatic backwards elimination and retained in the final model to improve fit but were not found to be significant upon the manual addition of the information theory variables. The Hosmer–Lemeshow test indicated an acceptable model fit (*p* = 0.887).

### 3.6. Sources of Advice for Owner-Reported Problem Behaviours

**Figure 9:**
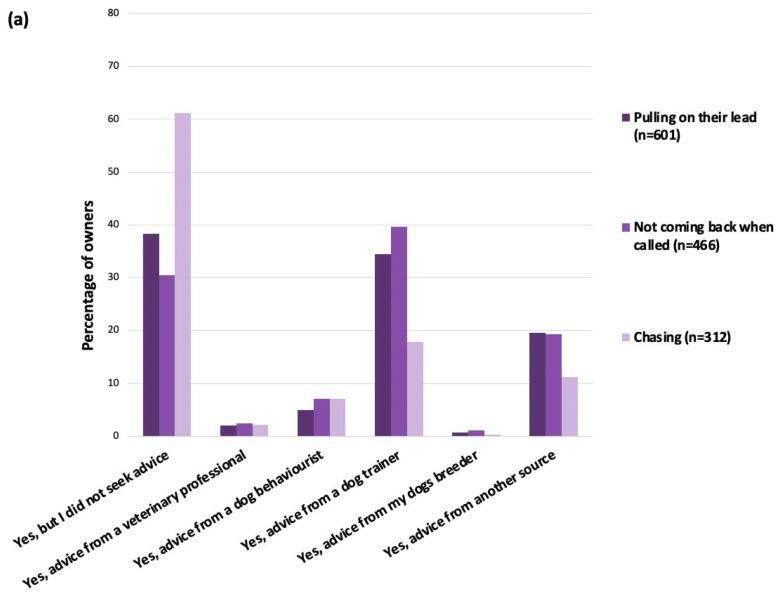

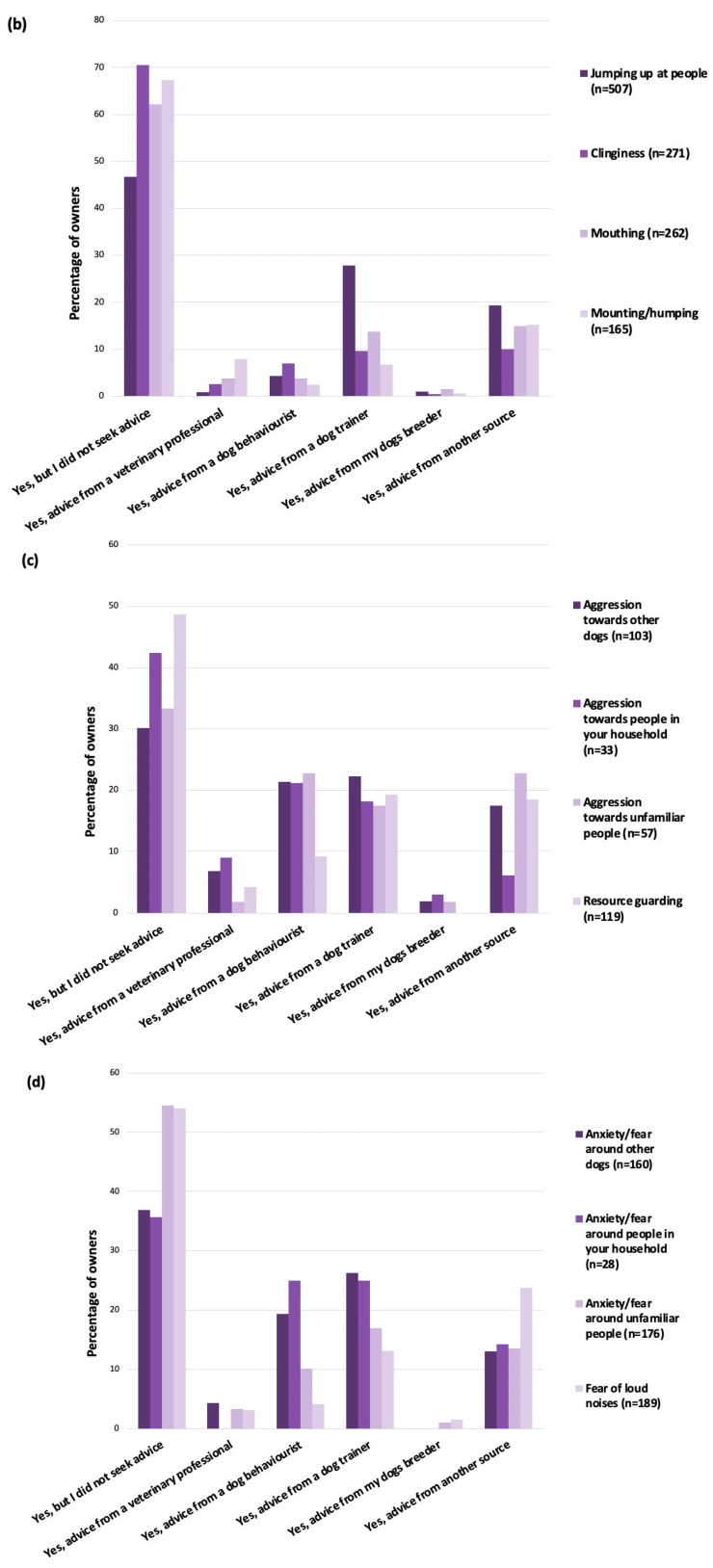

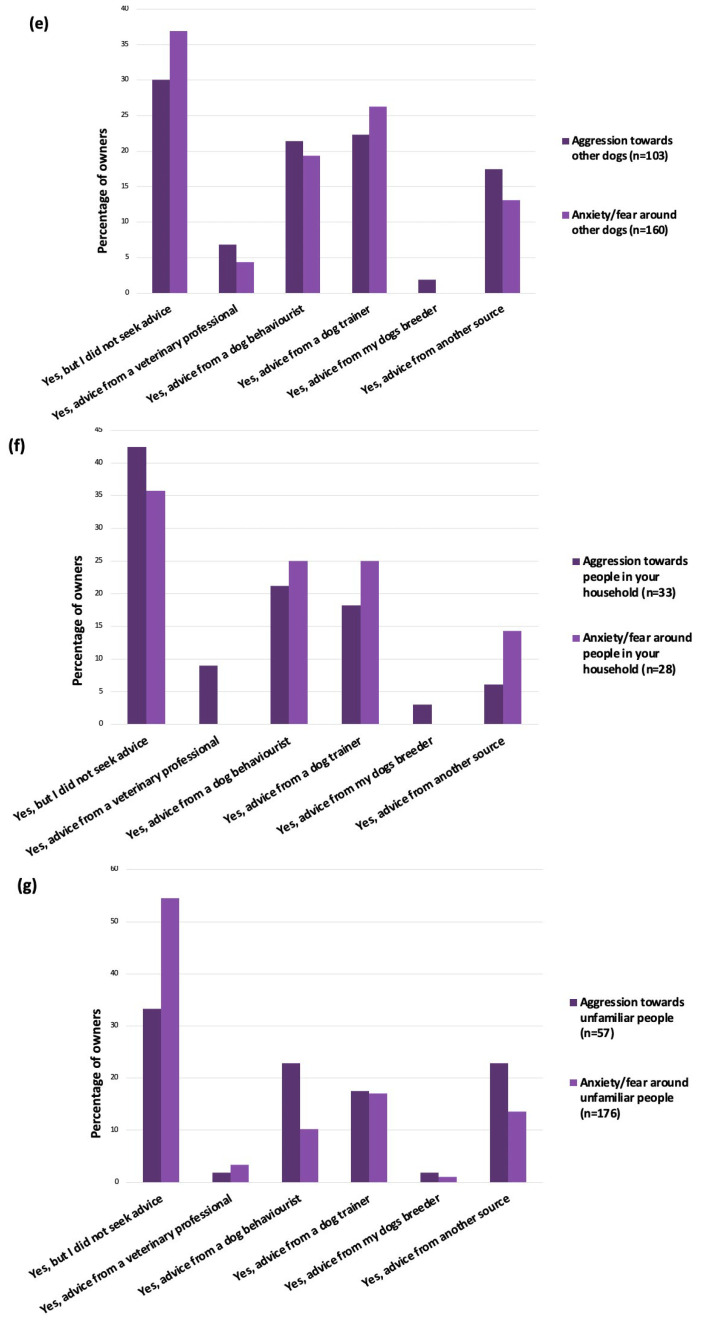

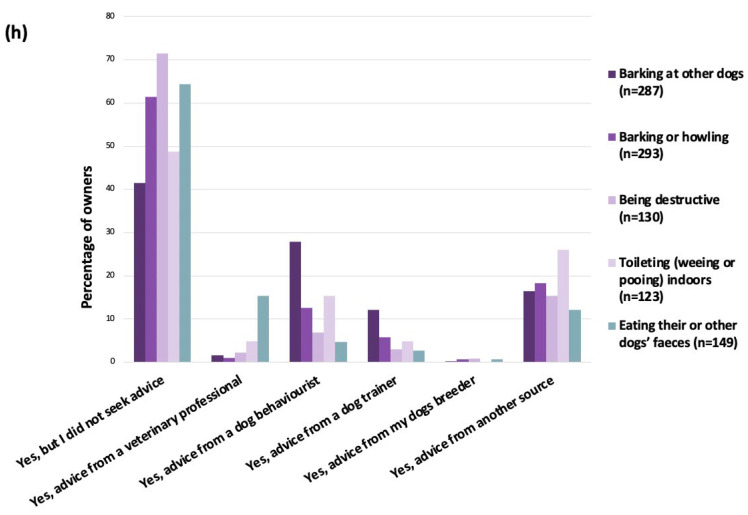
Distribution of advice sources used by owners for dogs displaying problem behaviours up to 21 months of age in a cohort of UK Pandemic Puppies acquired <16 weeks of age between 1 July and 31 December 2020. Advice sources used by owners who responded that their dog displayed the behaviours comprising the seven behaviour types [4] in addition to those uncategorised: (**a**) control behaviours; (**b**) attention-seeking behaviours; (**c**) aggressive behaviours; (**d**) fear/avoidance behaviours; (**e**) reaction to dogs’ behaviours; (**f**) reaction to familiar people behaviours; (**g**) reaction to unfamiliar people behaviours; and (**h**) uncategorised behaviours. Note that some behaviours in (**c**,**d**) are repeated in (**e**–**g**).

Across the 24 problem behaviours reported, the majority of owners did not seek any advice for their dog’s behaviour (Figure 9). The exception to this was owners who reported their dog had poor recall; these owners were more likely to have sought advice from a dog trainer (*n* = 185, 20.6%; total *n* = 466) than to have not sought any advice (Figure 9).

Owners were most likely to seek advice from a behaviourist if their dog displayed aggressive behaviour towards unfamiliar people (*n* = 13; 22.8%; total *n* = 57); aggressive behaviour towards dogs (*n* = 22; 21.4%; total *n* = 103); or aggressive behaviour towards familiar people (*n* = 7; 21.2%; total *n* = 33). Owners seeking advice from a veterinary professional was extremely rare (Figure 9), with the largest proportion of owners (*n* = 23, 15.4%; total *n* = 149) seeking advice from this source for coprophagia.

### 3.7. General Sources of Advice Training and Behaviour

When questioned about general advice sources for training and behaviour queries, the majority of owners (*n* = 583/893, 65.3%) cited their own experience from previous dog ownership followed by dog trainers (*n* = 497/895, 55.5%) and books (*n* = 439/892, 49.2%) (Table 10).

#### Risk Factor Analysis for Owners Seeking Professional Sources of Advice

Thirty-three of the forty-six variables assessed using univariable logistic regression were liberally associated (*p* <0.2) with the outcome measure “owners seeking professional sources of advice for general behaviour and training queries” (see Table S5, Supplementary Materials File S2).

Ten variables were identified via multivariable binary logistic regression modelling as being significantly associated with increased odds of owners seeking advice from professional sources since the first survey: attendance at adult dog or puppy training classes (compared to those who attended no classes); being a Golden Retriever (compared to a crossbreed); belonging to the KC Gundog group (compared to dogs of breeds/crossbreeds not KC-recognised); owners finding training and maintenance of their dogs obedience harder than expected (compared to those finding it better than expected or as expected); dogs displaying ≥1 aggressive behaviour, ≥1 attention-seeking behaviour, ≥1 fear/avoidance behaviour, or ≥1 reaction to dogs behaviour (compared to those that displayed none); total number of owner-reported problem behaviours per dog (Table 11).

The only variable that was found to be significantly associated with reduced odds of seeking advice from professional sources since the first survey was owners being aged 65–74 years old (compared to owners aged 45–54 years old) (Table 11).

A further six variables (conducting research prior to purchase; the dog being sold as a puppy outside the breeders home, without the dam present or without a microchip; control behaviour score; reaction to familiar people score; reaction to unfamiliar people behaviour score; categorisation as an SRB case) were highlighted using automatic backwards elimination and retained in the final model to improve fit, but were not found to be significant upon manual addition of information theory variables. Hosmer–Lemeshow test indicated an acceptable model fit (*p* = 0.668).

### 3.8. Owner Expectations of Their Dog’s Behaviour

A total of *n* = 407/896 (45.4%) of owners indicated that their dog’s behaviour up to 21 months of age met their expectations of behaviour soon after they acquired their puppy. A further *n* = 348 (38.8%) owners indicated their dog’s behaviour was better than they had expected; with a minority (*n* = 132, 14.7%) indicating their dog’s behaviour was worse than expected. A small number of respondents (*n* = 9, 1.0%) were either not sure or could not remember.

#### Risk Factor Analysis for Owners Finding Their Dogs Behaviour Worse Than Expected

Thirty-nine of the forty-nine variables assessed using univariable logistic regression were liberally associated (*p* < 0.2) with the outcome measure “owners finding their dogs behaviour worse than expected” (see Table S6, Supplementary Materials File S2).

Twelve variables were identified via multivariable binary logistic regression modelling as being significantly associated with increased odds of owners finding their dogs behaviour worse than expected: dogs displaying ≥1 aggressive behaviour, ≥1 attention-seeking behaviour, ≥1 control behaviour, ≥1 fear/avoidance behaviour, ≥1 reaction to dogs behaviour, ≥1 reaction to familiar people behaviour, or ≥1 reaction to unfamiliar people behaviour (compared to those that displayed none); total number of owner-reported problem behaviours per dog; dogs having a typical adult body weight of ≥40 kg (compared to 10 to <20 kg); attendance at adult training classes (compared to those who attended no classes) (Table 12).

Two variables were significantly associated with reduced odds of owners finding their dogs behaviour worse than expected: dogs being walked off-lead more than twice per day (compared to once per day); dogs being left alone >4 h (compared to never being left alone) (Table 12).

One variable (the dog being sold as a puppy without their dam present) was highlighted using automatic backwards elimination and retained in the final model to improve fit; however, this was not bound to be significant upon the manual addition of the information theory variables. The Hosmer–Lemeshow test indicated an acceptable model fit (*p* = 0.765).

### 3.9. Owner Expectations of Training and Maintaining Their Dog’s Obedience

A total of *n* = 507 (45.4%) of owners indicated that the training and maintenance of their dog’s obedience up to 21 months of age was as they expected soon after acquiring their puppy. A further *n* = 304 (32.9%) owners indicated the training and maintenance of their dog’s obedience was harder than they had expected, with a minority (*n* = 107, 11.6%) indicated the training and maintenance of their dog’s obedience was easier than they had expected. A small number of respondents (*n* = 5, 0.5%) were either not sure or could not remember.

#### Risk Factor Analysis for Owners Finding Training and Maintenance of Their Dog’s Obedience Harder Than Expected

Thirty-one of the forty-nine variables assessed using univariable logistic regression were liberally associated (*p* <0.2) with the outcome measure “owners finding training and maintenance of their dog’s obedience harder than expected” (see Table S7, Supplementary Materials File S2).

Twelve variables were identified via multivariable binary logistic regression modelling as being significantly associated with increased odds of owners finding their dogs training harder than expected: owners aged ≥75 years (compared to aged 45–54 years) or first-time dog owners (compared to experienced dog owners); dogs being a Golden Retriever (compared to crossbreed); dogs displaying ≥1 aggressive behaviour, ≥1 attention-seeking behaviour, ≥1 control behaviour, ≥1 fear/avoidance behaviour, ≥1 reaction to dogs behaviour, ≥1 reaction to familiar people behaviour, or ≥1 reaction to unfamiliar people behaviour (compared to those that displayed none); total number of owner-reported problem behaviours per dog; attendance at in-person adult training classes (compared to those who attended no classes); owners seeking professional sources of advice for general training and behaviour queries (compared to seeking advice from non-professional sources) (Table 13).

No variables were significantly associated with reduced odds of owners finding their dogs training harder than expected (Table 13).

A further three variables (current working location of owner; attendance at puppy classes <16 weeks of age; location dogs were left alone) were highlighted using automatic backwards elimination and retained in the final model to improve fit, but were not found to be significant upon the manual addition of the information theory variables. The Hosmer–Lemeshow test indicated an acceptable model fit (*p* = 0.810).

## 4. Discussion

This study longitudinally followed a large cohort of Pandemic Puppies purchased before 16 weeks of age in the UK during the 2020 phase of the COVID-19 pandemic as they reached adulthood. Within this unique and vulnerable population, we have described the prevalence of and identified risk factors for owner-reported problem behaviours, use of aversive training techniques, and negative expectation violation for owners regarding dog behaviour and training.

### 4.1. Owner-Reported Problem Behaviours

Our results indicated that the vast majority of owners of Pandemic Puppies (96.7%) reported that their dog exhibited at least one behaviour they considered problematic up to their dog being 21 months of age. The existing literature suggests that this prevalence is higher than similar studies of pre-pandemic owner-reported problem behaviour levels in the UK. Using a list of 36 problem behaviours (that were partially replicated in the present study), a prevalence of 76% of dogs displaying owner-reported problem behaviours was estimated (*n* = 192 total dogs) in a UK sample of dogs with a median age of 60 months [4]. It should be noted that estimates of the prevalence of owner-reported problem behaviours vary depending on the methods used to capture the data. For example, a recent UK-based longitudinal study used free text responses rather than predetermined lists to identify behaviours that the owners found problematic, which resulted in a markedly lower prevalence of 35% owner-reported problem behaviours at nine months of age (*n* = 784 total dogs) [12]; thus, the threshold for when a behaviour is considered “problematic” by an owner appears to be dependent on how the question is framed (as well as potential age-related differences in prevalence with regard to the cited example). The evidence suggests that ownership of almost all dogs is associated with the presence of some behavioural problems, as perceived by the owner, and this reality should be made more explicit to prospective owners prior to acquisition.

Among the problem behaviours reported, the most frequent were “control behaviours”, with 67.4% of owners reporting that their dog had a problem with pulling on the lead; this result is similar to the 69% of dogs reported by Blackwell et al. (2008) [4]. This consistency in the frequency over a 15-year period suggests little progress has been made on dealing with this issue, or it is intrinsic to dog ownership at this level. Exhibiting control behaviours was a significant risk factor for owners finding their dogs behaviour to be worse than expected, in agreement with a 2019 UK survey of *n* = 2183 dog owners, in which 24% of respondents reported lead pulling as the behaviour they would most like to change in their dog [53]. As well as impacting upon owners’ enjoyment of walks, lead pulling also has many potential negative welfare implications for dogs, including increased use of aversive training methods/aids to “correct” this behaviour, increased risk of physical injury to the dog, and reduced exercise provision [54]. These control behaviours, and particularly lead pulling prevention, are commonly covered in dog training classes [4]. Dog owners should therefore be encouraged to attend classes utilising rewards-based methods to reduce the likelihood of these common but challenging behaviours developing.

The Pandemic Puppy phenomenon has been implicated in international increases in dog bites to people during and following the COVID-19 pandemic [55,56]. In the current study, 25.1% of dogs were reported to have shown aggression, with the most common form being resource guarding (13.2%). However, even this high level is considerably lower than the 47% of dogs reported to display resource guarding in a previous UK-based study [4]. International prevalence estimates for dog aggression vary; for example, Salonen et al. (2020) [57] reported a prevalence of 14% for aggressive behaviours in a Finnish population of dogs that only included aggression towards familiar or unfamiliar people within their definition. A prevalence of 30% for aggressive behaviours was reported in a primarily US-based international sample of *n* = 4114 dogs with a median age of six years; however, this study did not include resource-guarding [58]. Given the importance of dog aggression as a reflection of an individual dog’s emotional wellbeing and to public health, greater focus should be placed on reducing the prevalence of aggression in dogs, given the range of effective interventions that are available to address this [59].

The current study identified diverse risk factors for the total number of problem behaviours reported, some of which have been speculated on during the pandemic regarding the impact of human lifestyle changes on dogs in the post-pandemic period [60,61,62]. Owners reporting more problem behaviours by 21 months was associated with an increased time of dogs being left alone compared to when under 16 weeks, particularly if this time threshold exceeded four hours. This result is of particular interest as the problem behaviour metric did not include SRBs; thus, it is a wider reflection of dog behaviour in the owners’ presence. This finding could be a result of dog’s expectations (set during their sensitive developmental period) being violated as adults, and their emotional and/or physical needs no longer being met due to this lifestyle change, e.g., via increased frustration, and reduced social enrichment during their time alone [63,64]. Similarly, dogs whose owners currently walk them less than once a week were more likely to exhibit a higher number of problem behaviours. This finding may represent reverse causality, i.e., reduced walking frequency by owners could be a response to problematic behaviours. However, reduced walking frequency could lead to deficits in physical and behavioural enrichment for these dogs that reduce emotional health, resulting in development of further behaviours that their owners find challenging [65]. Thus, the results of the current study indicate that educating owners regarding dog’s basic emotional and physical needs, and how they can be appropriately met, is of high importance. With specific regard to time left alone, prior to the acquisition of puppies, owners should critically reflect on whether they are able to maintain a consistent regime for how long their dog is likely to be left alone (aiming for <4 h at a time), and plan mitigation strategies for sudden increases (e.g., use of daycare if owners’ time outside of the house increases).

The current study identified use of aversive training techniques by owners as a key risk factor for the increased number of owner-reported problem behaviours, particularly when more than one aversive method/technique was used. Using punishment-based training has been correlated with increased problem behaviours in previous studies [5]. Furthermore, a review of existing studies on the effectiveness of aversive training techniques identified no evidence supporting aversive techniques being more effective than positive reinforcement training; indeed, some evidence reports that the opposite is true [66]. As such, the use of aversive methods being associated with an increased number of owner-reported problem behaviours in the current study is unsurprising; but it is concerning, particularly given the increased prevalence of using such techniques in this cohort and their negative welfare implications (discussed below; Section 4.2).

Despite strong media focus, and indeed legislative change over recent years regarding breed as an indicator of “dangerous” dog behaviour, the current study did not identify breed as a risk factor for increased owner-reported problem behaviours; instead, to a limited degree, breed was a protective factor, with owners of Gundogs, particularly English Springer Spaniels, found to be at reduced risk of reporting problem behaviours compared to owners of crossbred dogs. This result requires further research to fully understand genetic vs. environmental contributions; however, given that Gundogs were at particular risk of their owners using aversive training methods, their owners being less likely to report problem behaviours may not represent a positive welfare outcome if potential owner-reported problem behaviours are being suppressed via negative emotions (e.g., fear) [66].

### 4.2. Training Methods/Aids

The prevalence of using aversive methods/aids in the Pandemic Puppy cohort was high at around four in five owners (82.0%) and was higher compared to a number of previous studies in the UK over the past two decades (e.g., 74.2%, [41]; 72.0%, [4]; 79.8%, [5]). Owners whose dogs showed a range of owner-reported problem behaviours, including aggression, attention-seeking, and reactivity to familiar people, unfamiliar people, or other dogs, were more likely to use aversive methods; as discussed above, these are unlikely to resolve such behaviours and instead lead to increases in fear, anxiety [4,5,67], and aggression [13,68]. Studies have demonstrated that, because many owner-reported problem behaviours are likely to occur as a result of a dog’s negative emotional state, using aversive methods can exacerbate the issue [68,69,70]. A recent study demonstrated that dogs trained using one or more aversive method may have a more negative underlying mood compared to dogs trained without any aversive methods [71].

Owners who purchased their chosen breed/crossbreed because they perceived them to be easy to train were also at increased odds of using aversive techniques. A perception that a certain breed is easy to train may reflect owner deficits in understanding of canine behaviour (e.g., that all dogs require significant levels of training to avoid problem behaviours, and that “problem behaviours” often reflect emotional challenges in dogs, or that they not have been trained to exhibit an “appropriate” response). In the absence of this knowledge, and with high expectations of behaviour, owners may become frustrated that their dog is not behaving in a way that they expected, resulting in punishment. Furthermore, owners of dogs weighing 30 to 40 kg showed an increased likelihood of using aversive training techniques, consistent with previous research which indicated that owners of smaller dogs (<20 kg) used less aversive training methods [72]. Education on the use of rewards-based training methods should therefore be particularly focused towards owners of larger (>30 kg) dogs, emphasising that reward-based rather than aversive-based training methods can be effectively applied even for large dogs.

The only protective factor that reduced the likelihood of owners use of aversive methods/aids was attendance of online puppy classes by owners while their puppy was under 16 weeks of age. Given the significant efforts of dog behaviour organisations to pivot training activities from face-to-face to online during the pandemic, this is a positive result and demonstrates that dogs being present in a class is not essential in achieving good behaviour and welfare outcomes; this is likely due to the outcomes being dependent on educating the owner to train a dog appropriately, rather than training the dogs directly. Promotion of reward-based methods at this early stage of ownership and dog development appears key to shaping future dog–owner interactions; these dog–owner interactions were hypothesised to be worse than expected, given the high proportion of first-time owners in this cohort [25], with the owners being in need of greater support and education.

### 4.3. Behaviour and Training Advice Sources and Training Classes

Understanding why 82.3% of dog owners (*n* = 739/898) were motivated to use aversive methods/aids to train their dogs is an important dilemma given the potential negative welfare impacts of this owner choice. Key areas to consider here include passive owner exposure to training/behaviour information (e.g., via conversations with family/friends/other owners), as well as intentional information-seeking behaviours. Indeed, previous studies of information seeking among dog owners have highlighted concerns that dog owners generally utilise a diverse array of resources that are often poorly validated; here, internet resources comprised a popular choice [73]. In the current study, the most common sources of advice used by dog owners to gain dog behaviour and training information were owners’ own experiences of owning dogs in the past (65.3%) and dog trainers (55.5%). In addition, more than two in five owners used social media as a source of advice (42.3%) and more than one in three used television programmes (35.1%). Given dog training in the UK is currently unregulated [74,75], and behaviour/training information included on social media and TV is similarly unregulated and frequently raises concerns within the behavioural community regarding the promotion of aversive training methods [76,77], this is of high concern if it is promoting a wider culture of aversive training techniques in the UK.

Seeking professional advice from a suitably qualified and accredited dog behaviourist is considered to be the optimal method for owner information seeking for education on their dog’s behaviour [78]. Referral to a behaviourist is often required to occur via a veterinary surgeon, although there is evidence that this route may be suboptimal due to deficits in veterinary surgeon training and confidence regarding dog behaviour [79,80,81]. Seeking such professional advice may require owners to reach a perceptual threshold before taking this route, which may be more likely to be prohibited by cost than informal information-seeking routes, e.g., speaking to friends/family or searching online. Perhaps unsurprisingly, in the current study, those owners who found their dogs’ behaviour worse than they had initially expected at acquisition, and owners reporting an increased number of problem behaviours in their dog (particularly aggressive behaviour, fear/avoidance, reaction to other dogs and attention seeking), were significantly more likely to seek professional sources of advice for their dog. This may indicate that their threshold was initially lower or may have been higher but was exceeded in the first 21 months of their dog’s life (respectively). Reinforcing the idea that owners can take many preventative steps to improve dog behaviour and avoid problem behaviours developing is of importance, so that owners do not see professional advice as something that should only be sought once a problem is established (and thus potentially more challenging, time-consuming, and costly to address).

Owners who attended puppy training classes and adult dog training classes were significantly more likely to seek professional sources of advice for general behaviour, which could reflect these owners having established contacts within the behaviour/training industry, potentially from an earlier stage, that they could reach out to if problems were encountered. This source of owner education could also make owners more able to recognise problematic behaviours in their dog (e.g., rather than considering them normal or funny), which could also partially explain why owners who attended puppy training classes and adult dog training classes were more likely to report problem behaviours; however, the causality of this association is unknown and requires further investigation. Demographics were also found to influence advice seeking, with older owners aged 65–74 being less likely to seek professional advice for their dog’s training and behaviour, which may indicate a lack of a similar network, potentially due to reduced access to online resources that signpost professional sources [82], or because they had more opportunity to have previously owned a dog.

### 4.4. Separation-Related Behaviours

Just under one third of the dogs in the current cohort (30.9%) were defined as SRB cases; this is higher than the 22.1% reported in a previous pre-pandemic cohort of *n* = 1807 UK dogs (median age 4.25 years) [30], but similar to the 34% reported in another UK-based study (median age 6 years) [4]. It is notable that all three of these UK studies report higher prevalence than in other international studies, e.g., 5% of dogs in a Finnish population (median age 4.7 years) [57] and 13% in a primarily US-based population (median age 6 years) [58]; this may reflect differences in case definitions between countries, but could also reflect cultural differences in dog ownership that augment the likelihood of SRBs developing (e.g., how much time dogs spend with their owners as puppies, how dog-friendly their societies are, impacting how long dogs are left alone). SRBs were co-morbid with attention-seeking behaviours and a range of other fear/anxiety-related behaviours in this population, suggesting that dogs with SRBs in this population may have a more generalised anxiety phenotype in a range of contexts [83].

One potential explanation for the high levels of SRBs in the current Pandemic Puppy population could be the decreased time puppies were left alone during their sensitive developmental period, potentially creating an expectational blueprint in these dogs of their owners being a constant source of company, which may have been violated once lockdowns were lifted [84,85]. The current study identified that 55.2% of owners were leaving their dogs for an increased length of time aged 21 months compared to while their puppy was <16 weeks old, with 12.7% dogs now being left alone for >4 h. However, change in time left alone compared to whilst puppies were under 16 weeks was not found to be a risk factor in this population. It is possible that greater awareness by owners of SRB development, e.g., via media reports [86,87], motivated some owners to habituate their dog to increasing time left alone.

Protective factors for SRBs included growing up in a household with another dog. Although previous studies have implicated being left alone with another dog as a protective factor for SRBs [88], others have found no evidence for this [30,89,90]. This particular finding—of growing up with another dog in the household as a protective factor for SRBs—is novel, and it could be speculated that early social exposure to another dog who is able to relax in the absence of their owners could have a positive effect on new puppies raised in the same environment. However, this result requires further investigation, and should not be overinterpreted, particularly regarding existing SRB cases, where adding a new dog to a single-dog household where SRBs are already established could instead lead to two dogs with SRBs.

### 4.5. Owner Expectations

Over a third (32.9%) of owners reported that training their dog had been harder than expected, whilst over one in ten (14.7%) reported their dogs’ behaviour violated their expectations. A significant risk factor for finding training harder than expected was owners having no previous dog ownership experience, a demographic that was overrepresented in the Pandemic Puppies cohort. Indeed, previous research has indicated that first-time ownership is associated with increased levels of relinquishment [91]. A focus on managing prospective and new dog owners’ expectations of training, and providing practical training support (e.g., via low-cost classes or behavioural advice lines) should therefore be a priority for the animal welfare and behaviour sector.

Not surprisingly, all problem behaviour groupings were risk factors for owner expectation violations of both behaviour and training. However, this was not the case for SRBs, confirming that owners may not find some aspects of their dogs SRBs problematic (e.g., pacing), given they may not ever witness them or find evidence of them [90,92]. Attendance at adult dog training classes was a significant risk factor in both expectation models, likely due to reverse causality; owners who found that their dogs’ behaviour or training did not meet their expectations were more likely to actively seek help. In the case of training expectations, a similar relationship was seen: owners actively sought professional advice. In terms of demographics, owners over the age of 75 years were more likely to report that training their dog was harder than expected, which may reflect the physical demands of training a young dog that may be more challenging in the geriatric population. Further to this, owners of dogs weighing 40 kgs or more found their behaviour more challenging to manage than originally envisioned; this finding is consistent with those of previous research [72] and is likely to reflect the physicality of training a large dog.

Owners were less likely to report that their dog’s behaviour was worse than expected if they walked their dog off-lead more than twice per day. This could reflect that their dog has good recall and is not reactive to other dogs, making walking them a pleasant experience. It has been shown that one of the main positive expectations of dog ownership is increased walking [93], with dog walking frequency being negatively correlated with problem behaviours, including barking and escaping [15].

### 4.6. Limitations

In terms of the demographics of the owners in this study population, 90.2% were female; this is a similar bias to that identified in another UK longitudinal pet dog population study [94]. The demographics of the dogs also appeared to be affected by some biases, with a disproportionately low number of brachycephalic dogs relative to recent estimates of the most common breeds in the UK [95]. The low proportion of brachycephalic dogs in the current study is consistent with the findings of another UK longitudinal pet dog population survey [94]. The sub-population of owners examined in this study completed two large surveys; therefore, they may represent a cohort of committed owners, interested in canine welfare; if so, the results here are likely to represent a “best case scenario” for welfare in the wider population of dogs acquired during the pandemic. It is also possible that social desirability led to owners who had relinquished their dogs not responding, and some problem behaviours, training methods, and violations of expectations not being reported.

## 5. Conclusions

Levels of owner-reported behaviour problems and SRBs were elevated in the UK Pandemic Puppies cohort, which is of concern for the welfare of dogs in this vulnerable population. It is also concerning for the human–dog bond, given that so many owners appear to be struggling with diverse aspects of their dogs’ behaviour. Many Pandemic Puppy owners are first-time owners who were likely to be finding the training of their dog to be harder than they expected. This suggests a need for better setting of owner expectations of behaviour and training, even before acquisition, particularly amongst first-time dog owners, and a need for greater support for this owner demographic, e.g., provision of educational resources, training classes (online and in-person), and behaviour hotlines. More owners were found to be using aversive training methods with their dogs than at any point documented in the last two decades. Sector-wide efforts need to be made to turn the tide on this trend; however, behaviour and training advice that is commonly accessed by owners—that which is easily accessible and/or free—is often unreliable, unchecked, and in some cases, inhumane. Early educational intervention with owners (e.g., via online puppy classes, as demonstrated here) could be an effective way of promoting humane, effective, science-based techniques, whilst protecting owners from future misinformation. Given that dogs in this study were still at a relatively early age (<2 years), further efforts should be made to monitor this population as they age to understand the longer-term effects of pandemic-related acquisition and raising upon dog behaviour, including relinquishment risk, which may not yet be fully realised.

## Figures and Tables

**Table 1:** Categorisation of a list of commonly reported problem behaviours in dogs presented to owners, in addition to those derived from the free text via qualitative content analysis, into problem behaviour groups as per Blackwell et al. (2008) [4]. * Additional behaviour, which was only displayed in the presence of the owner and not the absence, was ascertained from the separation-related behaviour questions (see Section 2.4.2) and used in the categorisation of behaviours, but not in the “total number of owner-reported problem behaviours” metric. † New option derived from qualitative content analysis of free text to the response “Other” (Section 2.6) used in categorisation of behaviours, but not in the “total number of owner-reported problem behaviours” metric.

Problem Behaviour Group	Individual Behaviour Options
Control behaviours	Pulling on their lead
	Not coming back when called
	Chasing (e.g., cats, wildlife, traffic)
Attention-seeking behaviours	Jumping up at people
	Clinginess (e.g., following you, sitting close)
	Mouthing
	Mounting/humping other dogs, people, or objects
	* Destructive behaviour (e.g., chewing and causing damage to household items such as furniture, not including their own toys/treats)
Aggressive behaviours	Aggression towards other dogs
	Aggression towards people in your household (including you)
	Aggression towards unfamiliar people
	Guarding of food, toys, or other items
Fear/avoidance behaviours	Anxiety/fear around other dogs
	Anxiety/fear around people in your household (including you)
	Anxiety/fear around unfamiliar people
	Fear of loud noises (e.g., fireworks, thunderstorms) † Anxiety/fear of unfamiliar situations
Reaction to familiar people	Aggression towards people in your household (including you)
	Anxiety/fear around people in your household (including you)
Reaction to unfamiliar people	Aggression towards unfamiliar people
	Anxiety/fear around unfamiliar people
Reaction to other dogs	Aggression towards other dogs
	Anxiety/fear around other dogs
Not categorised	Barking at other dogs
	Barking or howling
	Being destructive
	Toileting (weeing or pooing) indoors
	Eating their or other dogs’ faeces
	† Frustration/overstimulation in certain situations
	† Problems sleeping overnight

**Table 2:** Categorisation of training aids/methods in the 21-month survey presented to owners of UK Pandemic Puppies acquired <16 weeks of age between 1 July and 31 December 2020. Division of common training methods/aids presented to owners into operant conditioning quadrants. † New option derived from qualitative content analysis of free text to the response “Other” (Section 2.6).

Operant Conditioning Quadrant Grouping	Individual Options
Rewards-based: Positive Reinforcement (R+)/Negative Punishment (P−)	Allowing sniffing time/interaction with the environment
	Clicker training (secondary conditioned stimulus)
	Food/treats
	Physical touch, e.g., stroking or patting
	Playing with other dogs
	Playing with you/another household member
	“Time out”: shutting your dog away within the home, away from people (e.g., in another room, in their crate)
	“Time out”: shutting your dog outdoors (e.g., in the garden/yard or an outside kennel)
	Toys
	Verbal praise
	† Restricting access to a desired behaviour/resource until an appropriate behaviour is displayed (e.g., stopping when your dog pulls on the lead, ignoring them if they jump up, etc.)
Aversive: Negative Reinforcement (R−)/Positive Punishment (P+)	Anti-bark “Husher” muzzle
	Bark-activated citronella/vibration/ultrasonic collar
	Choke/check chain
	Electric boundary fence
	Electric collar
	Head collar
	Lead corrections (e.g., quickly yanking back on/jerking the lead if your dog pulls)
	Pet Corrector™ (Chertsey, UK)
	Physical correction (e.g., smacking, tapping their nose, hitting, pinching)
	Physically moving your dog (e.g., pushing on your dog’s hindquarters to get them into a sit, pushing them off furniture or pushing them down if they jump up at you)
	Prong collar
	Rattle bottle/cans/discs as a distractor
	Rubbing their nose in faeces/urine if they toilet in an inappropriate location
	Shouting at them or telling them off
	Slip lead
	Water pistol/spray bottle
	† Chew deterrent (e.g., bitter apple spray, mustard, etc.)

**Table 3:** Categorisation of owner training aids/methods employed while training their dog. The methods/aids allocated to the four quadrants of operant conditioning and categorised as rewards-based (positive reinforcement/negative punishment) or aversive (negative reinforcement/positive punishment) were used to categorise owners training methods as reward-based-only; aversive-only; mixed: rewards-based with one aversive; mixed: rewards-based with more than one aversive.

Owner Training Method Category	Rewards-Based Methods/Aids	Aversive Methods/Aids
Rewards-based-only	≥1	0
Mixed: Rewards-based with 1 aversive	≥1	1
Mixed: Rewards-based with >1 aversive	≥1	≥2
Aversive-only	0	≥1

**Table 4:** Variables obtained during the original 2020 Pandemic Puppies study used to assess the potential legality of puppy purchases and risk of poor pre-purchase welfare of dogs [28]. The corresponding UK legislation is shown. † Due to the design of the original survey, it was not possible to identify dogs sold <8 weeks, only those sold <6 weeks of age. ‡ Due to the design of the original survey, it was not possible to identify dogs sold <15 weeks with a passport, only those sold <13 weeks of age with a passport.

Variable	UK Legislation
Sold without microchip details	The Microchipping of Dogs (Northern Ireland) Regulations, 2012 [43]; The Microchipping of Dogs (England) Regulations, 2012 [44]; The Microchipping of Dogs (Wales) Regulations, 2012 [45]; The Microchipping of Dogs (Scotland) Regulations, 2012 [46].
† Sold <8 weeks of age	The Animal Welfare (Licensing of Activities Involving Animals) (England) (Regulations, 2018) [18].
Collection without dam present	The Animal Welfare (Licensing of Activities Involving Animals) (England) (Amendment) Regulations, 2019 [27].
Collection outside of breeder’s property	The Animal Welfare (Licensing of Activities Involving Animals) (England) (Amendment) Regulations, 2019 [27].
‡ Sold <15 weeks of age with a passport	Commercial imports: The Balai Directive (Article 4 of Council Directive 92/65/EEC), Import Live Animals and Germinal Products to Great Britain under Balai Rules [47]. Non-commercial imports: Bringing Your Pet to Great Britain [48].

**Table 5:** Proportional problem behaviours reported by owners by 21 months of age in a cohort of UK Pandemic Puppies acquired <16 weeks of age between 1 July and 31 December 2020. * A separation-related behaviour displayed only in the presence of the owner and not the absence. † New options resulting from analysis of free text to the response “Other”.

Owner-Reported Problem Behaviour (Number of Respondents Answering This Option, Either Yes or No)	Frequency	
	*n* (Yes)	% (Yes)
Pulling on their lead (*n* = 892)	601	67.4
Jumping up at people (*n* = 890)	507	57.0
Not coming back when called (*n* = 898)	466	51.9
Chasing (e.g., cats, wildlife, traffic) (*n* = 897)	312	34.8
Barking or howling (*n* = 898)	293	32.6
Barking at other dogs (*n* = 899)	285	31.7
Clinginess (e.g., following you, sitting close) (*n* = 900)	271	30.1
Mouthing (*n* = 900)	262	29.1
Fear of loud noises (e.g., fireworks, thunderstorms) (*n* = 895)	189	21.1
Anxiety/fear around unfamiliar people (*n* = 901)	175	19.4
Mounting/humping other dogs, people, or objects (*n* = 900)	165	18.3
Anxiety/fear around other dogs (*n* = 902)	160	17.7
Eating their or other dogs’ faeces (*n* = 900)	149	16.6
Being destructive (*n* = 899)	130	14.5
Toileting (weeing or pooing) indoors (*n* = 898)	123	13.7
Guarding of food, toys, or other items (*n* = 902)	119	13.2
Aggression towards other dogs (*n* = 899)	103	11.5
* Destructive behaviour (e.g., chewing and causing damage to household items such as furniture, not including their own toys/treats) (*n* = 860)	73	8.5
Aggression towards unfamiliar people (*n* = 900)	57	6.3
† Frustration/overstimulation in certain situations (*n* = 738)	29	3.9
Aggression towards people in your household (including you) (*n* = 900)	33	3.7
Anxiety/fear around people in your household (including you) (*n* = 898)	28	3.1
† Anxiety/fear of unfamiliar situations (*n* = 738)	21	2.8
† Problems sleeping overnight (*n* = 738)	5	0.7

**Table 6:** Proportional problem behaviour groups [4] since the last survey, reported by owners when their dogs were 21 months of age, in a cohort of UK Pandemic Puppies acquired <16 weeks of age between 1 July and 31 December 2020.

Problem Behaviour Group (*n* = Total Number of Respondents to This Option, Either Yes or No)	Frequency	
	*n* (Yes)	% (Yes)
Control behaviours (*n* = 881)	742	84.2
Attention-seeking behaviours (*n* = 838)	648	77.3
Fear/avoidance behaviours (*n* = 888)	368	41.4
Aggressive behaviours (*n* = 893)	224	25.1
Reaction to other dogs (*n* = 896)	194	21.7
Reaction to unfamiliar people (*n* = 899)	189	21.0
Reaction to familiar people (*n* = 895)	52	5.8

**Table 7:** Final multivariable linear regression model for total number of problem behaviours per dog by 21 months of age reported by owners in a cohort of UK Pandemic Puppies acquired <16 weeks of age between 1 July and 31 December 2020 (*n* = 788). † Included as per information theory as a variable of a priori interest, regardless of significance. ‡ Dog demographic variables individually used to replace breed (most common 12 breeds at 21 months) in the original model. • Coefficient. * 95% confidence interval. Significant results in the table are formatted in bold font.

Variable	Category	B•	Std. Error	95% CI *	*p*-Value
Owner training method	Rewards-only			Ref	
	**Rewards with 1 aversive**	**1.00**	**0.34**	**0.32–1.67**	**0.004**
	**Rewards with >1 aversive**	**1.58**	**0.29**	**1.02–2.14**	**<0.001**
Exercise frequency (off-lead)	Never	0.47	0.48	−0.47–1.41	0.324
	**<Once a week**	**1.38**	**0.62**	**0.17–2.60**	**0.026**
	Once a week	1.05	0.59	−0.11–2.21	0.077
	Several times per week	0.36	0.31	−0.26–0.97	0.256
	Once per day			Ref	
	Twice per day	−0.16	0.28	−0.71–0.38	0.552
	>Twice per day	−0.39	0.33	−1.03–0.26	0.239
General advice source for training and behaviour	Non-professional			Ref	
	**Professional**	**1.17**	**0.23**	**0.72–1.63**	**<0.001**
Change in time dog left alone from <16 weeks	Same amount of time			Ref	
	Less time	0.66	0.64	−0.61–1.92	0.309
	More time (≤4 h)	0.07	0.24	−0.40–0.54	0.771
	**More time (>4 h)**	**1.01**	**0.47**	**0.08–1.94**	**0.033**
† Dog sex	Female			Ref	
	**Male**	**0.66**	**0.21**	**0.25–1.07**	**0.002**
Dog heard thunder <16 weeks	No			Ref	
	**Yes**	**−0.75**	**0.24**	**−1.22–−0.28**	**0.002**
†‡ Breed group	Not KC recognised			Ref	
	Toy	0.72	0.61	−0.49–1.92	0.243
	Hound	0.62	0.40	−0.16–1.40	0.118
	Pastoral	0.21	0.44	−0.64–1.07	0.626
	Utility	−0.59	0.43	−1.43–0.25	0.168
	Terrier	−0.68	0.40	−1.47–0.11	0.092
	Working	−0.68	0.55	−1.77–0.40	0.218
	**Gundog**	**−0.84**	**0.28**	**−1.39–−0.28**	**0.003**
Employed in animal care/veterinary sector	No			Ref	
	**Yes**	**−0.96**	**0.40**	**−1.74–−0.17**	**0.018**
Time left alone	Never			Ref	
	**Up to 1 h**	**−0.85**	**0.41**	**−1.64–−0.05**	**0.037**
	**1 to 4 h**	**−0.87**	**0.37**	**−1.59–−0.14**	**0.019**
	**Over 4 h**	**−1.26**	**0.55**	**−2.33–−0.19**	**0.021**
† Breed	Crossbred			Ref	
	Labradoodle	0.96	0.80	−0.61–2.53	0.231
	Miniature Smooth-Haired Dachshund	0.72	0.81	−0.87–2.31	0.376
			
	Border Collie	0.19	0.75	−1.28–1.66	0.541
	Cavapoo	−0.07	0.89	−1.81–1.68	0.939
	Cockapoo	−0.07	0.59	−1.24–1.10	0.906
	Cocker Spaniel	−0.29	0.63	−1.54–0.95	0.646
	Other	−0.30	0.52	−1.31–0.72	0.567
	Whippet	−0.67	0.83	−2.30–0.95	0.416
	Labrador Retriever	−1.03	0.59	−2.20–−0.13	0.083
	Border Terrier	−1.08	0.78	−2.60–0.45	0.166
	Golden Retriever	−1.28	0.78	−2.82–−0.25	0.101
	**English Springer Spaniel**	**−2.39**	**0.86**	**−4.11–−0.68**	**0.006**
† First-time dog owner	No			Ref	
	Yes	0.18	0.24	−0.29–0.66	0.450
† Owner age	18–24 years	0.06	0.55	−1.01–1.13	0.918
	25–34 years	−0.23	0.32	−0.85–0.40	0.479
	35–44 years	−0.46	0.31	−1.06–0.14	0.134
	45–54 years			Ref	
	55–64 years	−0.05	0.31	−0.66–0.57	0.878
	65–74 years	0.09	0.40	−0.69–0.86	0.827
	≥75 years	−0.82	0.99	−2.77–1.13	0.407
† Owner gender	Female			Ref	
	Male	−0.09	0.37	−0.81–0.64	0.815
† Dog Neutered	No			Ref	
	Yes	0.41	0.22	−0.03–0.85	0.065
†‡ Purebred status	Crossbred			Ref	
	Designer Crossbred	−0.09	0.54	−1.14–0.97	0.868
	Purebred	−0.50	0.52	−1.52–0.51	0.329
†‡ Typical adult bodyweight	≤10 kg	0.23	0.30	−0.36–0.81	0.449
	10 to <20 kg			Ref	
	20 to <30 kg	−0.37	0.28	−0.99–0.17	0.181
	30 to <40 kg	−0.60	0.33	−1.24–−0.04	0.067
	≥40 kg	1.05	0.83	−0.58–2.69	0.207

**Table 8:** Final multivariable binary logistic regression model for dogs categorised as separation-related behaviour cases, reported at 21 months of age in a cohort of UK Pandemic Puppies acquired <16 weeks of age between 1 July and 31 December 2020 (*n* = 709). † Included as per information theory as a variable of a priori interest, regardless of significance. ° Retained in model by selection during automatic backwards elimination. ‡ Dog demographic variables individually used to replace breed (most common 12 breeds at 21 months) in the original model. • Dog behaviour variables individually used to replace total number of undesirable behaviours in the original model. * 95% confidence interval. Significant results in the table are formatted in bold font.

Variable	Category	Odds Ratio	95% CI *	*p*-Value
• Number of attention-seeking behaviours	0		Ref	
	**≥1**	**2.34**	**1.46–3.76**	**<0.001**
† Owner age	18–24 years	1.42	0.62–3.27	0.410
	**25–34 years**	**1.71**	**1.03–2.83**	**0.038**
	35–44 years	1.21	0.73–2.00	0.463
	45–54 years		Ref	
	55–64 years	0.88	0.51–1.49	0.622
	65–≥75 years	0.53	0.25–1.09	0.083
• Number of fear/avoidance behaviours	0		Ref	
	**≥1**	**1.60**	**1.15–2.24**	**0.006**
Total number owner-reported undesirable behaviours		**1.10**	**1.04–1.17**	**0.001**
			
†‡ Typical adult bodyweight	≤10 kg	0.67	0.41–1.09	0.107
	10 to <20 kg		Ref	
	20 to <30 kg	0.77	0.49–1.21	0.255
	**30 to <40 kg**	**0.49**	**0.28–0.87**	**0.014**
	≥40 kg	0.62	0.17–2.30	0.474
Another dog(s) in household (in 2020)	No		Ref	
	**Yes**	**0.48**	**0.30–0.77**	**0.002**
Time left alone	Never		Ref	
	Up to 1 h	0.94	0.50–1.76	0.839
	1 to 4 h	0.58	0.33–1.01	0.054
	**Over 4 h**	**0.37**	**0.18–0.75**	**0.006**
† First-time dog owner	No		Ref	
	Yes	1.08	0.71–1.63	0.730
† Owner gender	Female		Ref	
	Male	1.02	0.55–1.89	0.941
† Dog sex	Female		Ref	
	Male	1.00	0.71–1.42	0.988
† Neutered	No		Ref	
	Yes	1.05	0.72–1.51	0.811
†‡ Purebred status	Crossbred		Ref	
	Purebred	1.08	0.46–2.57	0.587
	Designer Crossbred	1.51	0.62–3.66	0.365
† Breed	Crossbred		Ref	
	Whippet	2.22	0.60–8.24	0.236
	Border Collie	1.91	0.56–6.47	0.298
	Labradoodle	1.83	0.52–6.51	0.350
	Cocker Spaniel	1.77	0.63–5.01	0.279
	Cavapoo	1.76	0.43–7.26	0.431
	Border Terrier Cockapoo	1.71	0.47–6.23	0.414
		1.67	0.63–4.43	0.305
	English Springer Spaniel	1.21	0.28–5.23	0.802
	Labrador Retriever	1.05	0.39–2.83	0.923
	Other	1.01	0.42–2.43	0.977
	Golden Retriever	0.71	0.17–2.91	0.631
	Miniature Smooth-Haired Dachshund	0.61	0.15–2.37	0.470
		
†‡ Breed group	Not KC recognised		Ref	
	Working	0.99	0.41–2.38	0.977
	Hound	0.86	0.45–1.63	0.648
	Toy	0.86	0.31–2.37	0.768
	Pastoral	0.85	0.41–1.77	0.658
	Utility	0.83	0.42–1.66	0.596
	Gundog	0.80	0.51–1.25	0.324
	Terrier	0.75	0.36–1.56	0.443
° General advice source for training and behaviour	Non-professional		Ref	
	Professional	1.11	0.75–1.65	0.607
°• Number of control behaviours	0		Ref	
	≥1	1.45	0.88–2.41	0.147
°• Number of aggressive behaviours	0		Ref	
	≥1	1.09	0.75–1.59	0.655
°• Number of reaction to unfamiliar people behaviours	0		Ref	
	≥1	1.37	0.93–2.04	0.114

**Table 9:** Final multivariable binary logistic regression model for owners using ≥1 aversive method/aid(s) to train their dog up to the age of 21 months in a cohort of UK Pandemic Puppies acquired <16 weeks of age between 1 July and 31 December 2020 (*n* = 758). † Included as per information theory as a variable of a priori interest, regardless of significance. ° Retained in model by selection during automatic backwards elimination. ‡ Dog demographic variables individually used to replace breed (most common 11 breeds at 21 months) in the original model. • Dog behaviour variables individually used to replace total number of undesirable behaviours in the original model. * 95% confidence interval. Significant results in the table are formatted in bold font.

Variable	Category	Odds Ratio	95% CI *	*p*-Value
• Number of reaction to familiar people behaviours	0		Ref	
	**≥1**	**5.50**	**1.29–23.45**	**0.021**
†‡ Breed group	Not KC recognised		Ref	
	**Pastoral**	**4.80**	**1.31–17.59**	**0.018**
	**Gundog**	**2.70**	**1.51–4.83**	**<0.001**
	Terrier	1.28	0.58–2.84	0.539
	Utility	1.15	0.51–2.57	0.735
	Hound	0.94	0.44–2.00	0.874
	Working	0.88	0.35–2.17	0.773
	Toy	0.49	0.17–1.43	0.191
Time left alone	Never		Ref	
	Up to 1 h	0.89	0.41–1.93	0.762
	1 to 4 h	1.27	0.62–2.57	0.514
	**Over 4 h**	**3.23**	**1.18–8.85**	**0.023**
• Number of control behaviours	0		Ref	
	**≥1**	**2.82**	**1.79–4.45**	**<0.001**
†‡ Typical adult bodyweight	≤10 kg	0.63	0.37–1.08	0.091
	10 to <20 kg		Ref	
	20 to <30 kg	1.52	0.87–2.66	0.147
	**30 to <40 kg**	**2.36**	**1.13–4.89**	**0.022**
	≥40 kg	0.47	0.11–1.93	0.293
• Number of aggressive behaviours	0		Ref	
	**≥1**	**2.21**	**1.34–3.65**	**0.002**
• Number of reaction to unfamiliar people behaviours	0		Ref	
	**≥1**	**2.12**	**1.24–3.65**	**0.006**
• Number of attention-seeking behaviours	0		Ref	
	**≥1**	**2.02**	**1.30–3.14**	**0.002**
• Number of reaction to other dogs behaviours	0		Ref	
	**≥1**	**1.75**	**1.05–2.91**	**0.032**
Purchased breed/crossbreed because easy to train	No		Ref	
	**Yes**	**1.70**	**1.08–2.66**	**0.022**
Total number owner-reported undesirable behaviours		1.24	1.14–1.35	<0.001
			
Attended puppy training classes <16 weeks	No		Ref	
	Yes, in person	0.96	0.61–1.51	0.871
	**Yes, online**	**0.36**	**0.18–0.70**	**0.003**
† First-time dog owner	No		Ref	
	Yes	0.81	0.50–1.31	0.381
† Owner age	18–24 years	0.90	0.29–2.81	0.821
	25–34 years	0.86	0.46–1.59	0.620
	35–44 years	0.69	0.39–1.23	0.208
	45–54 years		Ref	
	55–64 years	0.89	0.48–1.66	0.702
	65–74 years	1.28	0.53–3.11	0.580
	≥75 years	1.53	0.17–13.82	0.707
† Owner gender	Female		Ref	
	Male	1.35	0.615–2.959	0.456
† Dog sex	Female		Ref	
	Male	1.27	0.84–1.91	0.262
† Neutered	No		Ref	
	Yes	1.09	0.71–1.70	0.690
†‡ Purebred status	Crossbred		Ref	
	Purebred	1.36	0.48–3.90	0.564
	Designer Crossbred	0.84	0.29–2.48	0.757
† Breed	Crossbred		Ref	
	Border Collie	4.16	0.42–40.77	0.222
	Border Terrier	2.77	0.44–17.28	0.275
	Labrador Retriever	2.17	0.62–7.62	0.225
	Golden Retriever	2.00	0.39–10.34	0.205
	Cocker Spaniel	1.34	0.37–4.86	0.660
	Miniature Smooth-Haired Dachshund	1.10	0.23–5.36	0.906
		
	Other	1.06	0.37–3.02	0.914
	Cockapoo	1.03	0.31–3.44	0.959
	Cavapoo	0.83	0.13–5.51	0.850
	Labradoodle	0.71	0.15–3.32	0.662
	Whippet	0.43	0.10–1.94	0.272
° Purchased dog to improve mental health	No		Ref	
	Yes	1.29	0.83–1.98	0.257
° Purchased breed/crossbreed because good with children	No		Ref	
	Yes	0.66	0.43–1.03	0.069
° Sold outside breeders’ home	No		Ref	
	Yes	1.47	0.95–2.27	0.088
°• Number of fear/avoidance behaviours	0		Ref	
	≥1	1.34	0.91–1.98	0.136

**Table 10:** Sources of general advice for training and behaviour for dogs up to 21 months old, amongst owners of dogs belonging to a cohort of UK Pandemic Puppies acquired <16 weeks of age between 1 July and 31 December 2020. † New options resulting from analysis of free text in response to the option “Other”.

Advice Source (*n* = Total Number of Respondents to This Option, Either Yes or No)	Frequency	
	*n* (Yes)	% (Yes)
My own experience from owning dogs in the past (*n* = 893)	583	65.3
Dog trainer (*n* = 895)	497	55.5
Book(s) (*n* = 892)	439	49.2
Friends or family who own or had owned a dog (*n* = 895)	424	47.4
Social media sites, e.g., Facebook, Instagram (*n* = 894)	378	42.3
TV programmes (*n* = 890)	312	35.1
A veterinary professional (e.g., veterinary surgeon, veterinary nurse) (*n* = 891)	295	33.1
YouTube (*n* = 888)	293	33.0
A breed/crossbreed-specific online resource (e.g., website/forum) (*n* = 892)	260	29.1
An animal charity website, e.g., Dogs Trust, RSPCA, PDSA, etc. (*n* = 891)	221	24.8
Dog behaviourist (*n* = 884)	166	18.8
My dog’s breeder (*n* = 880)	149	16.9
The Kennel Club website (*n* = 890)	103	11.6
Dog-specific magazine(s) (*n* = 879)	37	4.2
† An online training program/resource (e.g., APDT, IMDT, etc.) (*n* = 901)	13	1.4
† My own experience as I am a canine professional (*n* = 901)	6	0.7
† Another canine professional not listed above, e.g., dog walker, groomer, etc. (*n* = 901)	5	0.6
† A general online search (e.g., website/forum/internet search) (*n* = 901)	4	0.4

**Table 11:** Final multivariable binary logistic regression model of owners seeking professional advice for general training and behaviour queries up to their dog being 21 months of age, amongst owners of dogs from a cohort of UK Pandemic Puppies acquired <16 weeks of age between July and 31 December 2020 (*n* = 697). † Included as per information theory as a variable of a priori interest, regardless of significance. ° Retained in model by selection during automatic backwards elimination. ‡ Dog demographic variables individually used to replace breed (most common 11 breeds at 21 months) in the original model. • Dog behaviour variables individually used to replace total number of undesirable behaviours in the original model. * 95% confidence interval. Significant results in the table are formatted in bold font.

Variable	Category	Odds Ratio	95% CI *	*p*-Value
Attended adult training classes >16 weeks	No		Ref	
	**Yes**	**9.93**	**6.14–16.06**	**<0.001**
† Breed	Crossbred		Ref	
	**Golden Retriever**	**6.32**	**1.24–32.29**	**0.027**
	Cavapoo	4.12	0.38–44.78	0.244
	Labrador Retriever	2.11	0.66–6.69	0.205
	Whippet	1.85	0.41–8.28	0.423
	Border Terrier	1.25	0.30–5.30	0.762
	Cockapoo	1.20	0.36–3.94	0.769
	Border Collie	1.16	0.26–5.13	0.850
	Other	1.16	0.44–3.10	0.767
	Cocker Spaniel	0.76	0.22–2.61	0.658
	Miniature Smooth-Haired Dachshund	0.66	0.15–2.99	0.590
		
	Labradoodle	0.41	0.09–1.91	0.257
Attended puppy training classes <16 weeks	No		Ref	
	**Yes, in person**	**2.50**	**1.57–3.97**	**<0.001**
	**Yes, online**	**5.43**	**2.02–14.56**	**<0.001**
†‡ Breed group	Not KC recognised		Ref	
	**Gundog**	**2.20**	**1.23–3.92**	**0.008**
	Terrier	1.84	0.83–4.07	0.132
	Working	1.69	0.61–4.71	0.318
	Toy	1.24	0.40–3.89	0.711
	Hound	1.09	0.52–2.26	0.819
	Pastoral	1.06	0.44–2.55	0.897
	Utility	0.93	0.44–1.97	0.841
• Number of reaction to other dogs behaviours	0		Ref	
	**≥1**	**1.94**	**1.17–3.20**	**0.010**
• Number of aggressive behaviours	0		Ref	
	**≥1**	**1.86**	**1.16–2.99**	**0.010**
Owner expectations of training	As expected/easier		Ref	
	**Harder**	**1.73**	**1.06–2.84**	**0.030**
• Number of attention-seeking behaviours	0		Ref	
	**≥1**	**1.68**	**1.07–2.65**	**0.025**
• Number of fear/avoidance behaviours	0		Ref	
	**≥1**	**1.52**	**1.02–2.27**	**0.041**
Total number owner-reported undesirable behaviours		1.16	1.07–1.26	<0.001
			
† Owner age	18–24 years	1.07	0.37–3.15	0.897
	25–34 years	0.63	0.34–1.16	0.137
	35–44 years	0.77	0.42–1.40	0.385
	45–54 years		Ref	
	55–64 years	0.87	0.48–1.58	0.643
	**65–74 years**	**0.40**	**0.19–0.87**	**0.021**
	≥75 years	0.99	0.15–6.77	0.991
† First-time dog owner	No		Ref	
	Yes	1.34	0.80–2.27	0.268
† Owner gender	Female		Ref	
	Male	0.68	0.34–1.37	0.280
† Dog sex	Female		Ref	
	Male	0.89	0.59–1.33	0.568
† Neutered	No		Ref	
	Yes	1.37	0.89–2.12	0.152
†‡ Purebred status	Crossbred		Ref	
	Purebred	1.46	0.55–3.89	0.448
	Designer Crossbred	0.95	0.34–2.61	0.913
†‡ Typical adult bodyweight	≤10 kg	1.29	0.74–2.26	0.375
	10 to <20 kg		Ref	
	20 to <30 kg	1.21	0.71–2.08	0.489
	30 to <40 kg	1.15	0.62–2.15	0.662
	≥40 kg	0.69	0.17–2.80	0.604
° Research conducted prior to purchase	No		Ref	
	Yes	1.56	0.97–2.46	0.070
° Sold without dam present	No		Ref	
	Yes	1.39	0.83–2.35	0.214
° Sold without a microchip	No		Ref	
	Yes	0.62	0.25–1.53	0.301
°• Control behaviour score	0		Ref	
	≥1	1.14	0.69–1.91	0.608
°• Reaction to familiar people score	0		Ref	
	≥1	1.46	0.57–3.78	0.431
°• Reaction to unfamiliar people score	0		Ref	
	≥1	1.32	0.81–2.17	0.266
° SRB case	No		Ref	
	Yes	1.50	0.95–2.37	0.084

**Table 12:** Final multivariable binary logistic regression model for owners finding their dogs behaviour worse than expected up to 21 months of age relative to soon after acquisition amongst a cohort of UK Pandemic Puppies acquired <16 weeks of age between 1 July and 31 December 2020 (*n* = 756). † Included as per information theory as a variable of a priori interest, regardless of significance. ° Retained in model by selection during automatic backwards elimination. ‡ Dog demographic variables individually used to replace breed (most common 12 breeds at 21 months) in the original model. • Dog behaviour variables individually used to replace total number of undesirable behaviours in the original model. * 95% confidence interval. Significant results in the table are formatted in bold font.

Variable	Category	Odds Ratio	95% CI *	*p*-Value
• Number of reaction to familiar people behaviours	0		Ref	
	**≥1**	**5.90**	**2.97–11.74**	**<0.001**
• Number of reaction to other dogs behaviours	0		Ref	
	**≥1**	**5.70**	**3.63–8.97**	**<0.001**
• Number of control behaviours	0		Ref	
	**≥1**	**5.70**	**2.17–15.00**	**<0.001**
• Number of aggressive behaviours	0		Ref	
	**≥1**	**5.54**	**3.51–8.75**	**<0.001**
†‡ Typical adult bodyweight	≤10 kg	1.26	0.65–2.45	0.493
	10 to <20 kg		Ref	
	20 to <30 kg	1.34	0.71–2.56	0.370
	30 to <40 kg	1.86	0.90–3.83	0.094
	**≥** **40 kg**	**5.24**	**1.08–25.47**	**0.040**
Attended adult training classes >16 weeks	No		Ref	
	**Yes, in person**	**2.17**	**1.32–3.27**	**0.002**
	**Yes, online**	**3.68**	**1.39–9.76**	**0.009**
• Number of attention-seeking behaviours	0		Ref	
	**≥1**	**3.36**	**1.69–6.67**	**<0.001**
• Number of fear/avoidance behaviours	0		Ref	
	**≥1**	**2.71**	**1.77–4.16**	**<0.001**
• Number of reaction to unfamiliar people behaviours	0		Ref	
	**≥1**	**2.40**	**1.53–3.78**	**<0.001**
Total number owner reported undesirable behaviours		**1.41**	**1.30–1.53**	**<0.001**
			
Exercise frequency (off-lead)	Never	1.57	0.58–4.28	0.376
	<Once a week	0.64	0.15–2.68	0.540
	Once a week	1.80	0.63–5.12	0.272
	Several times per week	1.08	0.56–2.05	0.824
	Once per day		Ref	
	Twice per day	0.68	0.36–1.30	0.242
	**>Twice per day**	**0.26**	**0.10–0.68**	**0.006**
Change in time left alone from <16 weeks	Same amount of time		Ref	
	Less time	2.43	0.77–7.71	0.131
	More time (≤4 h)	0.65	0.39–1.08	0.098
	**More time (>4 h)**	**0.38**	**0.16–0.88**	**0.024**
† First-time dog owner	No		Ref	
	Yes	1.31	0.76–2.26	0.331
† Owner age	18–24 years	1.91	0.61–5.90	0.264
	25–34 years	2.01	0.99–4.10	0.055
	35–44 years	1.29	0.61–2.76	0.507
	45–54 years		Ref	
	55–64 years	1.62	0.79–3.33	0.186
	≥65 years	1.55	0.64–3.76	0.336
† Owner gender	Female		Ref	
	Male	0.48	0.18–1.28	0.144
† Dog sex	Female		Ref	
	Male	1.06	0.65–1.71	0.820
† Neutered	No		Ref	
	Yes	1.39	0.82–2.34	0.223
†‡ Purebred status	Crossbred		Ref	
	Purebred	1.30	0.45–3.73	0.632
	Designer Crossbred	0.77	0.25–2.38	0.646
† Breed	Crossbred		Ref	
	Border Collie	2.96	0.69–12.79	0.145
	Golden Retriever	2.19	0.39–12.18	0.372
	Labrador Retriever	1.69	0.48–5.89	0.411
	English Springer Spaniel	1.36	0.19–9.58	0.761
	Other	1.27	0.43–3.73	0.668
	Miniature Smooth-Haired Dachshund	0.90	0.15–5.41	0.909
		
	Cockapoo	0.66	0.18–2.51	0.546
	Border Terrier	0.62	0.09–4.49	0.634
	Labradoodle	0.57	0.10–3.41	0.537
	Cocker Spaniel	0.46	0.10–2.07	0.309
	Whippet	0.43	0.04–4.58	0.482
	Cavapoo	0.29	0.03–3.09	0.302
†‡ Breed group	Not KC recognised		Ref	
	Pastoral	2.26	0.96–5.35	0.063
	Terrier	1.54	0.62–3.83	0.354
	Gundog	1.36	0.72–2.59	0.344
	Working	1.31	0.37–4.61	0.674
	Utility	1.19	0.45–3.13	0.731
	Hound	1.18	0.47–2.94	0.728
	Toy	1.12	0.30–4.23	0.866
° Sold without dam present	No		Ref	
	Yes	1.47	0.85–2.56	0.170

**Table 13:** Final multivariable binary logistic regression model for owners finding training and maintaining their dog’s obedience harder than expected up to 21 months of age relative to soon after acquisition amongst a cohort of UK Pandemic Puppies acquired 1 July–31 December 2020 (*n* = 739). † Included as per information theory as a variable of a priori interest, regardless of significance. ° Retained in model by selection during automatic backwards elimination. ‡ Dog demographic variables individually used to replace breed (most common 12 breeds at 21 months) in the original model. • Dog behaviour variables individually used to replace total number of undesirable behaviours in the original model. * 95% confidence interval. Significant results in the table are formatted in bold font.

Variable	Category	Odds Ratio	95% CI *	*p*-Value
† Owner age	18–24 years	0.67	0.27–1.67	0.391
	25–34 years	1.52	0.92–2.53	0.105
	35–44 years	0.59	0.35–1.01	0.054
	45–54 years		Ref	
	55–64 years	0.99	0.55–1.80	0.982
	65–74 years	0.81	0.34–1.97	0.645
	**≥** **75 years**	**6.07**	**1.07–34.43**	**0.042**
† Breed	Crossbred		Ref	
	**Golden Retriever**	**4.13**	**1.10–15.48**	**0.035**
	Border Collie	3.10	0.84–11.50	0.091
	English Springer Spaniel	2.97	0.70–12.53	0.138
	Labradoodle	2.80	0.71–11.08	0.141
	Labrador Retriever	2.44	0.82–7.20	0.107
	Cocker Spaniel	2.43	0.77–7.68	0.130
	Whippet	2.37	0.57–9.80	0.234
	Cockapoo	2.29	0.77–6.76	0.135
	Other	1.94	0.73–5.15	0.182
	Border Terrier	1.91	0.45–8.14	0.380
	Miniature Smooth-Haired Dachshund	1.90	0.43–8.47	0.401
		
	Cavapoo	1.80	0.41–7.99	0.440
• Number of reaction to familiar people behaviours	0		Ref	
	**≥1**	**3.75**	**1.90–7.42**	**<0.001**
• Number of attention-seeking behaviours	0		Ref	
	**≥1**	**2.86**	**1.80–4.54**	**<0.001**
• Number of reaction to other dogs behaviours	0		Ref	
	**≥1**	**2.44**	**1.67–3.55**	**<0.001**
• Number of control behaviours	0		Ref	
	**≥1**	**2.38**	**1.39–4.05**	**0.001**
• Number of aggressive behaviours	0		Ref	
	**≥1**	**1.99**	**1.38–2.87**	**<0.001**
• Number of fear/avoidance behaviours	0		Ref	
	**≥1**	**1.99**	**1.43–2.76**	**<0.001**
General advice source for training and behaviour	Non-professional		Ref	
	**Professional**	**1.87**	**1.16–3.01**	**0.010**
• Number of reaction to unfamiliar people behaviours	0		Ref	
	**≥1**	**1.64**	**1.12–2.41**	**0.011**
Attended adult training classes >16 weeks	No		Ref	
	**Yes, in person**	**1.57**	**1.07–2.31**	**0.023**
	Yes, online	1.71	0.70–4.14	0.236
† First-time dog owner	No		Ref	
	**Yes**	**1.52**	**1.01–2.29**	**0.044**
Total number owner reported undesirable behaviours		**1.29**	**1.21–1.38**	**<0.001**
			
† Owner gender	Female		Ref	
	Male	0.84	0.45–1.58	0.588
† Dog sex	Female		Ref	
	Male	1.30	0.91–1.86	0.149
† Neutered	No		Ref	
	Yes	0.85	0.58–1.24	0.396
†‡ Purebred status	Crossbred		Ref	
	Purebred	2.34	0.89–6.16	0.085
	Designer Crossbred	1.88	0.70–5.09	0.214
†‡ Breed group	Not KC recognised		Ref	
	Utility	1.72	0.86–3.44	0.127
	Terrier	1.47	0.73–2.94	0.277
	Gundog	1.40	0.88–2.24	0.156
	Pastoral	1.37	0.65–2.87	0.411
	Working	1.04	0.38–2.87	0.936
	Toy	0.99	0.33–2.99	0.980
	Hound	0.85	0.42–1.72	0.659
†‡ Typical adult bodyweight	≤10 kg	1.06	0.64–1.74	0.833
	10 to <20 kg		Ref	
	20 to <30 kg	1.24	0.78–1.98	0.369
	30 to <40 kg	1.66	0.96–2.85	0.069
	≥40 kg	2.00	0.48–8.24	0.339
° Current working location of owner	At home	1.30	0.81–2.06	0.275
	Away from home	0.64	0.39–1.07	0.088
	At and away from home		Ref	
	Unemployed	0.96	0.37–2.51	0.934
	Retired	1.09	0.53–2.24	0.808
° Attended puppy classes <16 weeks	No		Ref	
	Yes, in person	1.10	0.75–1.62	0.626
	Yes, online	1.21	0.61–2.40	0.588
° Location left alone	A crate/indoor kennel (door closed)	1.40	0.84–2.31	0.193
	Loose in a single room	0.71	0.47–1.07	0.099
	Loose in multiple rooms		Ref	

## Data Availability

The data presented in this study are available on request from the corresponding author.

## Supplementary Materials

The following supporting information can be downloaded at: https://www.mdpi.com/article/10.3390/ani14020336/s1, File S1: Questionnaire; File S2: Supplementary Tables S1–S7; File S3: Qualitative Coding Framework; File S4: Demographics, Supplementary Tables S8–S11.
