# Supplementary material for: Impacts of Puppy Early Life Experiences, Puppy-Purchasing Practices, and Owner Characteristics on Owner-Reported Problem Behaviours in a UK Pandemic Puppies Cohort at 21 Months of Age
Source: Animals (Basel). 2024 Jan 22;14(2):336. doi: 10.3390/ani14020336 (PMC10812580; doi:10.3390/ani14020336)
Supplement: Supplementary file 1 [file animals-14-00336-s001.zip › File S2 Supplemental Tables Amended proofreading version.pdf]

# File S2:

## Supplementary Table S1: Predictive variables used for univariable analysis

**Table S1. Variables used for univariable analysis.**

Variables used for univariable analysis of the outcomes of (*i*) total number of problem behaviours per dog; (*ii*) SRB case; (*iii*) training methods used; owner expectations for (*iv*) behaviour and (*v*) training, and (*vi*) owner sources of advice for general behaviour and training queries †Categorical variable, binary. ‡Categorical variable, more than two categories. *Continuous variable. •Responses from the 2020 survey of "*I'm not sure*", "*I can't/don't remember*" or "*No, not as yet but I plan to before my dog is 16 weeks old (if applicable)*" recoded as missing data.

| | Variable | Survey derived from | Outcome(s) Tested |
|---|---|---|---|
| **Owner/household demographics** | †First-time dog owner, binary | 2020 | All |
| | ‡Owner age | 2020 | All |
| | †Owner gender | 2020 | All |
| | †Child ≤ 16 years in household | 2020 | All |
| | †•Employed in animal care/veterinary sector | 2020 | All |
| | †Another dog(s) in household | 2020 | All |
| | ‡Current working location of owner | 21 months | All |
| | ‡Current working location of other household members | 21 months | All |
| | †Owner lives alone/only adult in the household | 2020 | All |
| **Dog demographics** | †Dog sex | 2020 | All |
| | †Neuter status | 2020, updated 21 months | All |
| | ‡Breed purity categorisation | 2020 | All |
| | ‡Top 12 most common breeds at 21 months | 2020, analysed 21 months | All |
| | ‡UK Kennel Club breed group | 2020 | All |
| | ‡Typical adult bodyweight categorisation | 2020 | All |
| | †Insurance status | 2020, updated 21 months | Advice source |
| **Purchase motivations for a dog/breed or crossbreed chosen** | †Dog to improve mental health | 2020 | All except problem behaviours and SRBs |
| | †Breed/crossbreed size suited to owner's lifestyle | 2020 | All except problem behaviours and SRBs |
| | †Breed/crossbreed good with children | 2020 | All except problem behaviours and SRBs |
| | †Breed/crossbreed easy to train | 2020 | All except problem behaviours and SRBs |
| | †Friends or family own this breed/crossbreed | 2020 | All except problem behaviours and SRBs |
| | †Owner was driving force to get a dog | 2020 | All except problem behaviours and SRBs |
| | †Owner had more time to care for a dog due to COVID-19 pandemic | 2020 | All except problem behaviours and SRBs |

**Table S1. Contd.**

| | Variable | Survey derived from | Outcome(s) Tested |
|---|---|---|---|
| **Pre-purchase research** | †Research conducted | 2020 | All except problem behaviours and SRBs |
| **Purchase behaviours** | †Via animal-specific selling website | 2020 | All |
| | †Viewed in person prior to purchase | 2020 | All |
| **Potentially illegal sales indicators** | †•Sold without dam present | 2020 | All |
| | †Sold outside breeders' home | 2020 | All |
| | †•Sold without a microchip | 2020 | All |
| | †•Sold < 6 weeks of age | 2020 | All |
| | †•Sold with passport < 13 weeks of age | 2020 | All |
| **Owner expectations** | †Behaviour | 21 months | Advice source and training methods |
| | †Training | 21 months | Advice source and training methods |
| **Socialisation < 16 weeks** | ‡•Attended puppy classes | 2020, updated 21 months | All |
| | †•Met any people from outside their household | 2020 | Problem behaviours |
| | †•Had visitors to their home | 2020 | Problem behaviours |
| | †•Walked in a public space | 2020 | Problem behaviours |
| | †•Walked near traffic | 2020 | Problem behaviours |
| **Socialisation < 16 weeks** | †•Met any dogs from outside their household | 2020 | Problem behaviours |
| | †•Heard fireworks | 2020 | Problem behaviours |
| | †•Heard thunderstorm(s) | 2020 | Problem behaviours |
| | †•Visited a dog groomer | 2020 | Problem behaviours |
| | †•Deliberately left at home alone | 2020 | Problem behaviours and SRBs |
| **Adult management** | ‡Attended adult classes | 21 months | All |
| | ‡Owner training method | 21 months | All except training methods |
| | ‡Owner response to SRB | 21 months | SRBs |
| | ‡Exercise frequency (walks) | 21 months | All except advice source |
| | ‡Exercise frequency (off lead) | 21 months | All except advice source |
| | ‡Time left alone | 21 months | All except advice source |
| | ‡Change in time left alone from <16 weeks | 21 months | All |
| | ‡Location left alone | 21 months | All except advice source |
| | †General source of advice for training and behaviour | 21 months | All except advice source |
| | †Respondent is primary carer for dog | 2020 | Training methods and expectations |

**Table S1.** **Contd.**

| | Variable | Survey derived from | Outcome(s) Tested |
|---|---|---|---|
| **Dog behaviour** | *Total number of ‘problem behaviours’ | 21 months | All except problem behaviours |
| | †Number of ‘control behaviours’ | 21 months | All except problem behaviours |
| | †Number of ‘attention-seeking behaviours’ | 21 months | All except problem behaviours |
| | †Number of ‘aggressive behaviours’ | 21 months | All except problem behaviours |
| | †Number of ‘fear/avoidance behaviours’ | 21 months | All except problem behaviours |
| | †Number of ‘reaction to familiar people’ behaviours | 21 months | All except problem behaviours |
| | †Number of ‘reaction to unfamiliar people’ behaviours | 21 months | All except problem behaviours |
| | †Number of ‘reaction to dogs’ behaviours | 21 months | All except problem behaviours |
| | †SRB case | 21 months | All except problem behaviours and SRBs |

## Supplementary Table S2: Total number of owner-reported problem behaviours, univariable analysis

**Table S2.** **Descriptive and univariable analysis of total number of owner-reported problem behaviours at 21 months.**

Descriptive and univariable analysis (Mann-Whitney *U* or Kruskal-Wallis test as appropriate) of the total number of commonly reported problem behaviours per dog with no missing data based upon responses to the question *"At any point since the last survey (in November/December 2020) have you considered that your dog has a problem with the following behaviours? Please select all options that apply."*. Owners who had answered "*I'm not sure*" were reclassified as missing data. Three behaviours from qualitative content analysis of free-text responses were included used along with a common separation-related behaviour which was only displayed in the presence of the owner and not the absence. Significant results are emboldened.

| | Variable | Category | % | Median* | IQR | Statistics | | |
|---|---|---|---|---|---|---|---|---|
| | | | | | | *U* | $X^2$ | *p*-Value |
| **Owner/household demographics** | First-time dog owner (*n* = 787) | Yes | 39.1 | 5.00 | 3.00 – 7.00 | | | |
| | | No | 60.9 | 4.00 | 3.00 – 7.00 | 64425.000 | N/A | **0.003** |
| | Owner age (*n* = 787) | 18-24 years | 4.5 | 5.00 | 2.00 – 8.00 | | | |
| | | 25-34 years | 19.4 | 5.00 | 3.00 – 7.00 | | | |
| | | 35-44 years | 19.4 | 4.00 | 3.00 – 6.00 | | | |
| | | 45-54 years | 28.1 | 5.00 | 3.00 – 7.00 | N/A | 3.052 | 0.802 |
| | | 55-64 years | 18.2 | 5.00 | 3.00 – 7.00 | | | |
| | | 65-74 years | 9.3 | 5.00 | 3.00 – 7.00 | | | |
| | | ≥ 75 years | 1.1 | 4.00 | 3.50 – 6.50 | | | |
| | Owner gender (*n* = 787) | Female | 91.1 | 5.00 | 3.00 – 7.00 | | | |
| | | Male | 8.9 | 5.00 | 2.75 – 7.00 | 25746.500 | N/A | 0.718 |
| | Child ≤ 16 years in household (in 2020) (*n* = 787) | No | 73.1 | 5.00 | 3.00 – 7.00 | | | |
| | | Yes | 26.9 | 4.00 | 3.00 – 6.00 | 58201.500 | N/A | 0.329 |
| | Employed in animal care/veterinary sector (*n* = 788) | No | 92.4 | 5.00 | 3.00 – 7.00 | | | |
| | | Yes | 7.6 | 4.00 | 2.00 – 6.00 | 17230.000 | N/A | **0.006** |
| | Another dog(s) in household (in 2020) (*n* = 788) | No | 72.6 | 5.00 | 3.00 – 7.00 | | | |
| | | Yes | 27.4 | 4.00 | 2.00 – 6.00 | 50486.500 | N/A | **< 0.001** |

*Median number of total owner-reported problem behaviours.

**Table S2. Contd…**

| | Variable | Category | % | Median* | IQR | Statistics | | |
|---|---|---|---|---|---|---|---|---|
| | | | | | | $U$ | $X^2$ | $p$-Value |
| **Owner/household demographics** | Current working location of owner ($n$ = 774) | At home | 23.4 | 5.00 | 3.00 – 7.00 | | | |
| | | Away from home | 20.9 | 5.00 | 3.00 – 7.00 | | | |
| | | At and away from home | 31.9 | 4.00 | 3.00 – 7.00 | N/A | 2.880 | 0.578 |
| | | Unemployed | 3.6 | 5.00 | 3.00 – 8.00 | | | |
| | | Retired | 20.2 | 4.00 | 3.00 – 6.00 | | | |
| | Current working location of other household members ($n$ = 675) | At home | 19.0 | 5.00 | 3.00 – 6.00 | | | |
| | | Away from home | 32.7 | 4.00 | 3.00 – 7.00 | | | |
| | | At and away from home | 31.4 | 5.00 | 3.00 – 7.00 | N/A | 5.347 | 0.254 |
| | | Unemployed | 1.5 | 6.50 | 3.75 – 10.00 | | | |
| | | Retired | 15.4 | 4.00 | 3.00 – 6.00 | | | |
| | Owner lives alone/only adult in the household ($n$ = 774) | No | 86.6 | 5.00 | 3.00 – 7.00 | | | |
| | | Yes | 13.4 | 4.00 | 3.00 – 7.00 | 33646.000 | N/A | 0.571 |
| **Dog demographics** | Dog sex ($n$ = 787) | Female | 46.9 | 4.00 | 3.00 – 7.00 | | | |
| | | Male | 53.1 | 5.00 | 3.00 – 7.00 | 69176.000 | N/A | **0.012** |
| | Neutered ($n$ = 788) | No | 40.9 | 4.00 | 2.00 – 6.00 | | | |
| | | Yes | 59.1 | 5.00 | 3.00 – 7.00 | 86871.000 | N/A | **< 0.001** |
| | Breed purity designation ($n$ = 788) | Crossbred | 4.3 | 5.00 | 4.00 – 8.00 | | | |
| | | Purebred | 68.5 | 4.00 | 3.00 – 7.00 | | | |
| | | Designer Crossbred | 27.2 | 5.00 | 3.00 – 7.00 | N/A | 10.889 | **0.004** |
| | Top 12 most common breeds at 21 months ($n$ = 788) | Crossbred | 4.3 | 5.00 | 4.00 – 8.00 | | | |
| | | Cockapoo | 9.9 | 5.00 | 4.00 – 8.00 | | | |
| | | Labradoodle | 2.8 | 5.50 | 3.00 – 8.25 | | | |
| | | Cavapoo | 1.9 | 6.00 | 4.00 – 8.00 | | | |
| | | Labrador Retriever | 10.3 | 4.00 | 2.00 – 6.00 | | | |
| | | Cocker Spaniel | 6.9 | 5.00 | 3.00 – 7.00 | | | |
| | | Border Collie | 3.4 | 5.00 | 4.00 – 8.00 | N/A | 30.130 | **0.003** |
| | | Miniature Smooth-Haired Dachshund | 2.8 | 5.50 | 2.75 – 9.25 | | | |
| | | Border Terrier | 2.9 | 4.00 | 3.00 – 6.00 | | | |
| | | Golden Retriever | 2.9 | 4.00 | 2.00 – 6.00 | | | |
| | | Whippet | 2.4 | 4.00 | 2.00 – 7.00 | | | |
| | | English Springer Spaniel | 2.0 | 3.00 | 2.25 – 4.00 | | | |
| | | Other | 47.5 | 4.50 | 3.00 – 7.00 | | | |

*Median number of total owner-reported problem behaviours.

**Table S2. Contd…**

| | Variable | Category | % | Median* | IQR | Statistics | | |
|---|---|---|---|---|---|---|---|---|
| | | | | | | *U* | $X^2$ | *p*-Value |
| **Dog demographics** | Breed group (*n* = 788) | Not KC recognised | 32.3 | 5.00 | 3.00 – 7.00 | | | |
| | | Gundog | 28.7 | 4.00 | 2.00 – 6.00 | | | |
| | | Hound | 9.3 | 5.00 | 3.00 – 8.00 | | | |
| | | Pastoral | 7.1 | 5.00 | 3.00 – 8.00 | N/A | 24.579 | **< 0.001** |
| | | Terrier | 8.6 | 5.00 | 3.00 – 6.75 | | | |
| | | Toy | 3.0 | 5.50 | 3.25 – 6.75 | | | |
| | | Utility | 6.9 | 4.00 | 2.75 – 6.25 | | | |
| | | Working | 4.1 | 3.50 | 2.00 – 6.75 | | | |
| | Typical adult bodyweight (*n* = 756) | ≤ 10 kg | 19.2 | 5.00 | 3.00 – 7.00 | | | |
| | | 10 to < 20 kg | 41.1 | 5.00 | 3.00 – 7.00 | | | |
| | | 20 to < 30 kg | 22.8 | 4.00 | 2.25 – 6.00 | N/A | 12.679 | **0.013** |
| | | 30 to < 40 kg | 15.2 | 4.00 | 2.00 – 6.00 | | | |
| | | ≥ 40 kg | 1.7 | 4.00 | 2.00 – 10.00 | | | |
| **Purchase behaviours** | Via animal-specific selling website (*n* = 787) | No | 48.8 | 4.00 | 3.00 – 6.00 | | | |
| | | Yes | 51.2 | 5.00 | 3.00 – 7.00 | 84928.000 | N/A | **0.017** |
| | Viewed in person prior to purchase (*n* = 788) | No | 29.3 | 5.00 | 3.00 – 7.00 | | | |
| | | Yes | 70.7 | 5.00 | 3.00 – 7.00 | 61852.500 | N/A | 0.391 |
| **Potentially illegal sales indicators** | Sold without dam present (*n* = 786) | No | 22.1 | 4.00 | 3.00 – 7.00 | | | |
| | | Yes | 77.9 | 5.00 | 3.00 – 7.00 | 48907.500 | N/A | 0.099 |
| | Sold outside breeders' home (*n* = 788) | No | 63.2 | 5.00 | 3.00 – 7.00 | | | |
| | | Yes | 36.8 | 5.00 | 3.00 – 7.00 | 70680.500 | N/A | 0.618 |
| | Sold without a microchip (*n* = 772) | No | 95.7 | 5.00 | 3.00 – 7.00 | | | |
| | | Yes | 4.3 | 6.00 | 3.50 – 8.00 | 10495.000 | N/A | 0.173 |
| | Sold < 6 weeks of age (*n* = 787) | No | 99.7 | 5.00 | 3.00 – 7.00 | | | |
| | | Yes | 0.3 | 6.50 | 4.00 – 9.00 | 1030.000 | N/A | 0.443 |
| | Sold with passport < 13 weeks of age (*n* = 714) | No | 94.5 | 5.00 | 3.00 – 7.00 | | | |
| | | Yes | 5.5 | 4.00 | 2.00 – 7.00 | 11786.500 | N/A | 0.269 |

*Median number of total owner-reported problem behaviours.

**Table S2. Contd…**

| | Variable | Category | % | Median* | IQR | Statistics | | |
|---|---|---|---|---|---|---|---|---|
| | | | | | | *U* | $X^2$ | *p*-Value |
| **Socialisation < 16 weeks** | Attended puppy classes (*n* = 787) | No | 56.8 | 5.00 | 3.00 – 7.00 | | | |
| | | Yes, in person | 35.5 | 5.00 | 3.00 – 7.00 | N/A | 0.398 | 0.820 |
| | | Yes, online | 7.8 | 4.00 | 3.00 – 7.00 | | | |
| | Met any people from outside their household (*n* = 733) | No | 2.9 | 4.00 | 1.00 – 7.50 | | | |
| | | Yes | 97.1 | 5.00 | 3.00 – 7.00 | 8090.500 | N/A | 0.518 |
| | Had visitors to their home (*n* = 707) | No | 8.6 | 4.00 | 3.00 – 7.00 | | | |
| | | Yes | 91.4 | 5.00 | 3.00 – 7.00 | 20412.000 | N/A | 0.640 |
| | Walked in a public space (*n* = 623) | No | 8.3 | 6.00 | 3.25 – 8.00 | | | |
| | | Yes | 91.7 | 5.00 | 3.00 – 7.00 | 12214.000 | N/A | **0.033** |
| | Walked near traffic (*n* = 637) | No | 9.1 | 5.00 | 3.00 – 8.00 | | | |
| | | Yes | 90.9 | 4.00 | 3.00 – 7.00 | 15263.000 | N/A | 0.250 |
| | Met any dogs from outside their household (*n* = 666) | No | 11.4 | 4.00 | 2.25 – 7.75 | | | |
| | | Yes | 88.6 | 5.00 | 3.00 – 7.00 | 23128.000 | N/A | 0.652 |
| | Fireworks (*n* = 715) | No | 39.2 | 5.00 | 3.00 – 7.00 | | | |
| | | Yes | 60.8 | 5.00 | 3.00 – 7.00 | 60217.500 | N/A | 0.799 |
| | Thunderstorms (*n* = 643) | No | 63.8 | 5.00 | 3.00 – 7.00 | | | |
| | | Yes | 36.2 | 4.00 | 2.00 – 6.00 | 41007.500 | N/A | **0.003** |
| | Dog groomer (*n* = 652) | No | 79.0 | 4.00 | 3.00 – 6.00 | | | |
| | | Yes | 21.0 | 5.00 | 3.00 – 7.00 | 38854.000 | N/A | 0.066 |
| | Deliberately left at home alone (*n* = 681) | No | 15.9 | 5.00 | 3.00 – 8.00 | | | |
| | | Yes | 84.1 | 4.00 | 3.00 – 6.00 | 26429.500 | N/A | **0.016** |
| **Adult management** | Attended adult classes (*n* = 788) | No | 54.7 | 4.00 | 3.00 – 6.00 | | | |
| | | Yes, in person | 41.6 | 5.00 | 3.00 – 7.00 | N/A | 7.421 | **0.024** |
| | | Yes, online | 3.7 | 5.00 | 3.00 – 8.50 | | | |
| | Owner training method (*n* = 762) | Rewards-based only | 17.6 | 3.00 | 2.00 – 5.00 | | | |
| | | Rewards-based & 1 aversive | 20.3 | 5.00 | 3.00 – 6.00 | N/A | 38.081 | **< 0.001** |
| | | Rewards-based & > 1 aversive | 62.1 | 5.00 | 3.00 – 7.00 | | | |
| | | Aversive only | 0.0 | / | / | | | |

*Median number of total owner-reported problem behaviours.

**Table S2. Contd…**

| | Variable | Category | % | Median* | IQR | Statistics | | |
|---|---|---|---|---|---|---|---|---|
| | | | | | | *U* | $X^2$ | *p*-Value |
| **Adult management** | Exercise frequency (walks) (*n* = 784) | Never | 0.1 | 4.00 | *n* = 1 | | | |
| | | < Once a week | 0.3 | 4.00 | 0.00 – 8.00 | | | |
| | | Once a week | 0.3 | 4.50 | 0.00 – 9.00 | | | |
| | | Several times per week | 5.5 | 5.00 | 3.00 – 8.00 | N/A | 1.947 | 0.924 |
| | | Once per day | 27.2 | 4.00 | 3.00 – 7.00 | | | |
| | | Twice per day | 45.9 | 5.00 | 3.00 – 7.00 | | | |
| | | > Twice per day | 20.8 | 4.00 | 3.00 – 7.00 | | | |
| | Exercise frequency (off lead) (*n* = 782) | Never | 5.6 | 5.00 | 3.00 – 8.75 | | | |
| | | < Once a week | 3.2 | 6.00 | 3.50 – 9.50 | | | |
| | | Once a week | 3.3 | 6.50 | 3.75 – 9.25 | | | |
| | | Several times per week | 16.0 | 5.00 | 3.00 – 8.00 | N/A | 22.435 | **0.001** |
| | | Once per day | 32.1 | 5.00 | 3.00 – 7.00 | | | |
| | | Twice per day | 25.1 | 4.00 | 3.00 – 6.00 | | | |
| | | > Twice per day | 14.7 | 4.00 | 2.00 – 6.00 | | | |
| | Time left alone (*n* = 788) | Never | 10.2 | 6.00 | 3.00 – 8.00 | | | |
| | | Up to 1 hour | 18.7 | 5.00 | 3.00 – 6.00 | N/A | 7.835 | **0.050** |
| | | 1 to 4 hours | 58.1 | 4.00 | 3.00 – 7.00 | | | |
| | | Over 4 hours | 13.1 | 5.00 | 3.00 – 7.00 | | | |
| | Change in time left alone from < 16 weeks (*n* = 772) | Same amount of time | 41.6 | 5.00 | 3.00 – 6.00 | | | |
| | | Less time | 2.8 | 4.50 | 2.75 – 8.25 | N/A | 4.899 | 0.179 |
| | | More time ≤ 4 hours | 43.5 | 4.00 | 3.00 – 7.00 | | | |
| | | More time > 4 hours | 12.0 | 5.00 | 3.00 – 8.00 | | | |
| | Location left alone (*n* = 770) | A crate/indoor kennel (door closed) | 15.2 | 5.00 | 3.00 – 8.00 | | | |
| | | Loose in a single room | 28.2 | 5.00 | 3.00 – 7.00 | | | |
| | | Loose in multiple rooms | 56.2 | 4.00 | 3.00 – 7.00 | N/A | 6.573 | 0.254 |
| | | Outdoor kennel (door closed) | 0.1 | / | / | | | |
| | | Outdoor kennel (door open) | 0.1 | / | / | | | |
| | | Loose in an enclosed outdoor space | 0.1 | / | / | | | |
| | General advice source for training and behaviour (*n* = 781) | Non-professional | 29.7 | 4.00 | 2.00 – 6.00 | | | |
| | | Professional | 70.3 | 5.00 | 3.00 – 7.00 | 78581.500 | N/A | **< 0.001** |

*****Median number of total owner-reported problem behaviours.

## Supplementary Table S3: Total number of owner-reported separation-related behaviours, univariable analysis

**Table S3.** **Descriptive and univariable analysis of owner-reported separation-related behaviour cases at 21 months.**

Descriptive and univariable binary logistic regression analysis of the binary categorisation of separation-related behaviour cases based upon nine commonly reported separation-related behaviours presented to owners. Dogs not displaying individual behaviour(s) in either context were coded as '0'; those who only displayed an individual behaviour(s) whilst left alone were coded as '1'. Dogs displaying individual behaviour(s) in both contexts were coded as '2', whilst those dogs only displaying individual behaviour(s) whilst relaxing were coded as '3'. To be coded as a 'case' at least one behaviour was a '1', any/all of the remaining behaviours could be a '2' or a '3'. *95% confidence interval. Significant results are emboldened. ~ No analysis possible.

| | Variable | Category | ***n* (not a case; case)** | % (case) | Odds Ratio | 95% CI* | ***p*-Value** |
|---|---|---|---|---|---|---|---|
| **Owner/ household demographics** | First-time dog owner (*n* = 788) | No | 359; 125 | 25.8 | | Ref | |
| | | **Yes** | **185; 119** | **39.1** | **1.847** | **1.359 – 2.512** | **< 0.001** |
| | Owner age (*n* = 788) | 18-24 years | 23; 13 | 36.1 | 1.422 | 0.677 – 2.990 | 0.353 |
| | | **25-34 years** | **85; 65** | **43.3** | **1.925** | **1.239 – 2.989** | **0.004** |
| | | 35-44 years | 103; 50 | 32.7 | 1.222 | 0.778 – 1.918 | 0.384 |
| | | 45-54 years | 151; 60 | 28.4 | | Ref | |
| | | 55-64 years | 111; 39 | 26.0 | 0.884 | 0.552 – 1.417 | 0.609 |
| | | 65-74 years | 64; 16 | 20.0 | 0.629 | 0.337 – 1.174 | 0.146 |
| | | ≥ 75 years | 7; 1 | 12.5 | 0.360 | 0.043 – 2.985 | 0.343 |
| | Owner gender (*n* = 789) | Female | 493; 223 | 31.1 | | Ref | |
| | | Male | 52; 21 | 28.8 | 0.893 | 0.525 – 1.518 | 0.675 |
| | Child ≤ 16 years in household (in 2020) (*n* = 788) | No | 397; 174 | 30.5 | | Ref | |
| | | Yes | 148; 69 | 31.8 | 1.064 | 0.760 – 1.490 | 0.719 |
| | Employed in animal care/veterinary sector (*n* = 789) | No | 505; 229 | 31.2 | | Ref | |
| | | Yes | 40; 15 | 27.3 | 0.827 | 0.448 – 1.527 | 0.544 |
| | Another dog(s) in household (in 2020) (*n* = 781) | No | 361; 202 | 35.9 | | Ref | |
| | | **Yes** | **177; 41** | **18.8** | **0.414** | **0.283 – 0.606** | **< 0.001** |
| | Current working location of owner (*n* = 778) | At home | 116; 59 | 33.7 | 0.893 | 0.595 – 1.341 | 0.585 |
| | | Away from home | 123; 46 | 27.2 | 0.657 | 0.429 – 1.006 | 0.053 |
| | | At and away from home | 158; 90 | 36.3 | | Ref | |
| | | Unemployed | 17; 9 | 34.6 | 0.929 | 0.398 – 2.171 | 0.866 |
| | | **Retired** | **124; 36** | **22.5** | **0.510** | **0.324 – 0.801** | **0.004** |

**Table S3.** **Contd…**

| | Variable | Category | *n* (not a case; case) | % (case) | Odds Ratio | 95% CI* | *p*-Value |
|---|---|---|---|---|---|---|---|
| **Owner/ household demographics** | Current working location of other household members (*n* = 674) | At home | 84; 42 | 33.3 | 1.075 | 0.671 – 1.720 | 0.764 |
| | | Away from home | 147; 75 | 33.8 | 1.097 | 0.734 – 1.639 | 0.653 |
| | | At and away from home | 144; 67 | 31.8 | | Ref | |
| | | Unemployed | 7; 3 | 30.0 | 0.921 | 0.231 – 3.673 | 0.907 |
| | | **Retired** | **89; 16** | **15.2** | **0.386** | **0.211 – 0.708** | **0.002** |
| | Owner lives alone/only adult in the household (*n* = 778) | No | 469; 201 | 30.0 | | Ref | |
| | | Yes | 69; 39 | 36.1 | 1.319 | 0.861 – 2.019 | 0.203 |
| **Dog demographics** | Dog sex (*n* = 788) | Female | 263; 108 | 29.1 | | Ref | |
| | | Male | 281; 136 | 32.6 | 1.179 | 0.870 – 1.596 | 0.289 |
| | Neutered (*n* = 789) | No | 234; 89 | 27.6 | | Ref | |
| | | Yes | 311; 155 | 33.3 | 1.310 | 0.960 – 1.788 | 0.088 |
| | Breed designation (*n* = 789) | Crossbred | 24; 10 | 29.4 | | Ref | |
| | | Purebred | 391; 153 | 28.1 | 0.939 | 0.439 – 2.010 | 0.872 |
| | | Designer Crossbred | 130; 81 | 38.4 | 1.495 | 0.680 – 3.289 | 0.317 |
| | Breed (*n* = 789) | Other Crossbred | 87; 45 | 34.1 | | Ref | |
| | | Cockapoo | 45; 31 | 40.8 | 1.332 | 0.744 – 2.384 | 0.335 |
| | | Labradoodle | 14; 9 | 39.1 | 1.243 | 0.500 – 3.092 | 0.640 |
| | | Cavapoo | 8; 6 | 42.9 | 1.450 | 0.474 – 4.435 | 0.515 |
| | | Labrador Retriever | 61; 22 | 26.5 | 0.697 | 0.380 – 1.278 | 0.243 |
| | | Cocker Spaniel | 35; 21 | 37.5 | 1.160 | 0.606 – 2.222 | 0.654 |
| | | Border Collie | 18; 11 | 37.9 | 1.181 | 0.514 – 2.715 | 0.694 |
| | | Miniature Smooth-Haired Dachshund | 18; 7 | 28.0 | 0.752 | 0.292 – 1.933 | 0.554 |
| | | Border Terrier | 14; 7 | 33.3 | 0.967 | 0.364 – 2.566 | 0.946 |
| | | Golden Retriever | 20; 5 | 20.0 | 0.483 | 0.170 – 1.373 | 0.172 |
| | | Whippet | 12; 7 | 36.8 | 1.128 | 0.415 – 3.063 | 0.814 |
| | | English Springer Spaniel | 12; 6 | 33.3 | 0.967 | 0.340 – 2.746 | 0.949 |
| | | Other Purebred | 201; 67 | 25.0 | 0.644 | 0.409 – 1.015 | 0.058 |

**Table S3.** **Contd…**

| | Variable | Category | *n* (not a case; case) | % (case) | Odds Ratio | 95% CI* | *p*-Value |
|---|---|---|---|---|---|---|---|
| **Dog demographics** | Breed group (*n* = 789) | Not KC recognised | 161; 91 | 36.1 | | Ref | |
| | | **Gundog** | **169; 64** | **27.5** | **0.670** | **0.456 – 0.985** | **0.042** |
| | | Hound | 49; 25 | 33.8 | 0.903 | 0.523 – 1.558 | 0.713 |
| | | Pastoral | 41; 17 | 29.3 | 0.734 | 0.394 – 1.365 | 0.328 |
| | | Terrier | 47; 15 | 24.2 | 0.565 | 0.299 – 1.066 | 0.078 |
| | | Toy | 14; 7 | 33.3 | 0.885 | 0.345 – 2.271 | 0.799 |
| | | Utility | 42; 15 | 26.3 | 0.632 | 0.332 – 1.202 | 0.162 |
| | | Working | 22; 10 | 31.3 | 0.804 | 0.365 – 1.773 | 0.589 |
| | Typical adult bodyweight (*n* = 758) | ≤ 10 kg | 99; 41 | 29.3 | 0.740 | 0.481 – 1.139 | 0.171 |
| | | 10 to < 20 kg | 202; 113 | 35.9 | | Ref | |
| | | 20 to < 30 kg | 126; 50 | 28.4 | 0.709 | 0.475 – 1.059 | 0.093 |
| | | **30 to < 40 kg** | **90; 24** | **21.1** | **0.477** | **0.287 – 0.790** | **0.004** |
| | | ≥ 40 kg | 9; 4 | 30.8 | 0.794 | 0.239 – 2.638 | 0.707 |
| **Purchase behaviours** | Via animal-specific selling website (*n* = 788) | No | 278; 97 | 25.9 | | Ref | |
| | | **Yes** | **266; 147** | **35.6** | **1.584** | **1.166 – 2.152** | **0.003** |
| | Viewed in person prior to purchase (*n* = 789) | No | 164; 69 | 29.6 | | Ref | |
| | | Yes | 381; 175 | 31.5 | 1.092 | 0.782 – 1.524 | 0.606 |
| **Potentially illegal sales indicators** | Sold without dam present (*n* = 788) | No | 431; 189 | 30.5 | | Ref | |
| | | Yes | 113; 55 | 32.7 | 1.110 | 0.771 – 1.599 | 0.575 |
| | Sold outside breeders' home (*n* = 789) | No | 337; 164 | 32.7 | | Ref | |
| | | Yes | 208; 80 | 27.8 | 0.790 | 0.575 – 1.087 | 0.147 |
| | Sold without a microchip (*n* = 775) | No | 509; 232 | 31.3 | | Ref | |
| | | Yes | 25; 9 | 26.5 | 0.790 | 0.363 – 1.719 | 0.552 |
| | Sold < 6 weeks of age (*n* = 788) | No | 544; 243 | 30.9 | | Ref | |
| | | Yes | 0; 1 | 100.0 | ~ | ~ | ~ |
| | Sold with passport < 13 weeks of age (*n* = 719) | No | 467; 213 | 31.3 | | Ref | |
| | | Yes | 28; 11 | 28.2 | 0.861 | 0.421 – 1.763 | 0.683 |
| **Socialisation < 16 weeks** | Attended puppy classes (*n* = 788) | No | 318; 138 | 30.3 | | Ref | |
| | | Yes, in person | 186; 86 | 31.6 | 1.065 | 0.770 – 1.474 | 0.702 |
| | | Yes, online | 41; 19 | 31.7 | 1.068 | 0.598 – 1.906 | 0.824 |
| | Deliberately left at home alone (*n* = 691) | No | 68; 41 | 62.4; 37.6 | | Ref | |
| | | Yes | 414; 168 | 71.1; 28.9 | 0.673 | 0.439 – 1.032 | 0.069 |

**Table S3. Contd…**

| | Variable | Category | *n* (not a case; case) | % (case) | Odds Ratio | 95% CI* | *p*-Value |
|---|---|---|---|---|---|---|---|
| **Adult management** | Attended adult classes (*n* = 789) | No | 290; 148 | 33.8 | | Ref | |
| | | Yes, in person | 234; 90 | 27.8 | 0.754 | 0.551 – 1.031 | 0.077 |
| | | Yes, online | 21; 6 | 22.2 | 0.560 | 0.221 – 1.417 | 0.221 |
| | Owner training method (*n* = 766) | Rewards-based only | 98; 39 | 28.5 | | Ref | |
| | | Rewards-based & 1 aversive | 106; 44 | 29.3 | 1.043 | 0.626 – 1.739 | 0.872 |
| | | Rewards-based & > 1 aversive | 321; 158 | 33.0 | 1.237 | 0.815 – 1.877 | 0.318 |
| | | Aversive only | 0; 0 | 0.0 | ~ | ~ | ~ |
| | Owner response to SRB (*n* = 212) | No response | 32; 73 | 69.5 | | Ref | |
| | | Positive response | 26; 56 | 68.3 | 0.944 | 0.506 – 1.762 | 0.857 |
| | | Aversive response | 7; 13 | 65.0 | 0.814 | 0.297 – 2.232 | 0.689 |
| | | Mixed response | 1; 4 | 80.0 | 1.753 | 0.188 – 16.312 | 0.622 |
| | Exercise frequency (walks) (*n* = 785) | Never | 0; 0 | 0.0 | ~ | ~ | ~ |
| | | < Once a week | 0; 2 | 100.0 | ~ | ~ | ~ |
| | | Once a week | 1; 0 | 0.0 | ~ | ~ | ~ |
| | | Several times per week | 26; 18 | 40.9 | 1.594 | 0.839 – 3.028 | 0.154 |
| | | Once per day | 146; 71 | 32.7 | 1.120 | 0.780 – 1.608 | 0.540 |
| | | Twice per day | 251; 109 | 30.3 | | Ref | |
| | | > Twice per day | 118; 43 | 26.7 | 0.839 | 0.554 – 1.271 | 0.408 |
| | Exercise frequency (off lead) (*n* = 783) | Never | 28; 14 | 33.3 | 0.970 | 0.485 – 1.941 | 0.932 |
| | | < Once a week | 20; 8 | 28.6 | 0.776 | 0.328 – 1.836 | 0.564 |
| | | Once a week | 19; 8 | 29.6 | 0.817 | 0.343 – 1.944 | 0.648 |
| | | Several times per week | 93; 36 | 27.9 | 0.751 | 0.471 – 1.197 | 0.229 |
| | | Once per day | 163; 84 | 34.0 | | Ref | |
| | | Twice per day | 134; 61 | 31.3 | 0.883 | 0.591 – 1.319 | 0.545 |
| | | > Twice per day | 83; 32 | 27.8 | 0.784 | 0.460 – 1.216 | 0.241 |
| | Time left alone (*n* = 789) | Never | 48; 34 | 41.5 | | Ref | |
| | | Up to 1 hour | 89; 53 | 37.3 | 0.841 | 0.482 – 1.465 | 0.540 |
| | | **1 to 4 hours** | **333; 129** | **27.9** | **0.547** | **0.337 – 0.887** | **0.015** |
| | | **Over 4 hours** | **75; 28** | **27.2** | **0.527** | **0.284 – 0.977** | **0.042** |

**Table S3.** **Contd…**

| | Variable | Category | *n* (not a case; case) | % (case) | Odds Ratio | 95% CI* | *p*-Value |
|---|---|---|---|---|---|---|---|
| **Adult management** | Change in time left alone from < 16 weeks (*n* = 774) | Same amount of time | 218; 96 | 30.6 | | Ref | |
| | | **Less time** | **9; 15** | **62.5** | **3.785** | **1.601 – 8.949** | **0.002** |
| | | More time ≤ 4 hours | 239; 101 | 29.7 | 0.960 | 0.687 – 1.341 | 0.809 |
| | | More time > 4 hours | 69; 27 | 28.1 | 0.889 | 0.536 – 1.473 | 0.647 |
| | Location left alone (*n* = 768) | A crate/indoor kennel (door closed) | 85;30 | 26.1 | 0.747 | 0.470 – 1.186 | 0.215 |
| | | Loose in a single room | 152; 66 | 30.3 | 0.918 | 0.646 – 1.306 | 0.636 |
| | | Loose in multiple rooms | 294; 139 | 32.1 | | Ref | |
| | | Outdoor kennel (door closed) | 1; 0 | 0.0 | ~ | ~ | ~ |
| | | Outdoor kennel (door open) | 0; 0 | 0.0 | ~ | ~ | ~ |
| | | Loose in an enclosed outdoor space | 1; 0 | 0.0 | ~ | ~ | ~ |
| | General advice source for training and behaviour (*n* = 778) | Non-professional | 176; 58 | 24.8 | | Ref | |
| | | **Professional** | **364; 180** | **33.1** | **1.501** | **1.061 – 2.122** | **0.022** |
| **Dog behaviour** | Total number of owner-reported undesirable behaviours (*n* = 726) | / | / | / | **1.126** | **1.069 – 1.186** | **< 0.001** |
| | | / | / | / | | | |
| | Control behaviour score (*n* = 769) | 0 | 95; 28 | 22.8 | | Ref | |
| | | **1+** | **436; 210** | **32.5** | **1.634** | **1.039 – 2.569** | **0.033** |
| | Attention seeking behaviour score (*n* = 765) | 0 | 145; 28 | 16.2 | | Ref | |
| | | **1+** | **389; 203** | **34.3** | **2.702** | **1.743 – 4.191** | **< 0.001** |
| | Aggressive behaviour score (*n* = 779) | 0 | 415; 172 | 29.3 | | Ref | |
| | | 1+ | 124; 68 | 35.4 | 1.323 | 0.937 – 1.868 | 0.112 |
| | Fear/avoidance behaviour score (*n* = 776) | 0 | 340; 121 | 26.2 | | Ref | |
| | | **1+** | **197; 118** | **37.5** | **1.683** | **1.236 – 2.291** | **< 0.001** |
| | Reaction to familiar people (*n* = 783) | 0 | 515; 224 | 30.3 | | Ref | |
| | | 1+ | 28; 16 | 36.4 | 1.314 | 0.697 – 2.477 | 0.399 |
| | Reaction to unfamiliar people (*n* = 782) | 0 | 435; 181 | 29.4 | | Ref | |
| | | 1+ | 106; 60 | 36.1 | 1.360 | 0.948 – 1.952 | 0.095 |
| | Reaction to other dogs (*n* = 780) | 0 | 427; 183 | 30.0 | | Ref | |
| | | 1+ | 113; 57 | 33.5 | 1.177 | 0.819 – 1.691 | 0.378 |

## Supplementary Table S4: Owner training method score, descriptive and univariable analysis

**Table S4. Descriptive and univariable analysis of owner training methods at 21 months.**

Descriptive and univariable analysis of the binary categorisation of 27 training methods/aids presented to participants in response to the question "*Have you or anyone in your household ever used any of the following aids or methods on/with your dog, to try and change any aspect of their behaviour?*". Methods/aids were allocated to the four quadrants of operant conditioning and categorised as rewards-based (positive reinforcement/negative punishment) or aversive (negative reinforcement/positive punishment). An additional reward method/aid and an additional aversive method/aid from qualitative content analysis of free-text responses was also included. Owners who reported using only positive reinforcement or negative punishment aids/methods were categorised as rewards-based only. Those using a mix of all four operant conditioning quadrants were categorised as mixed. No respondents reported using only negative reinforcement or positive punishment (aversive only). Significant results are emboldened. ~ No analysis possible.

| | Variable | Category | *n* (reward only; mixed) | % (mixed) | Odds Ratio | 95% CI* | *p*-Value |
|---|---|---|---|---|---|---|---|
| **Owner/ household demographics** | First-time dog owner (*n* = 895) | No | 92; 455 | 83.2 | | Ref | |
| | | Yes | 65; 283 | 81.3 | 0.880 | 0.620 – 1.250 | 0.476 |
| | Owner age (*n* = 894) | 18-24 years | 5; 32 | 86.5 | 1.108 | 0.405 – 3.031 | 0.842 |
| | | 25-34 years | 31; 139 | 81.8 | 0.776 | 0.459 – 1.313 | 0.345 |
| | | 35-44 years | 37; 131 | 78.0 | 0.613 | 0.369 – 1.019 | 0.059 |
| | | 45-54 years | 36; 208 | 85.2 | | Ref | |
| | | 55-64 years | 32; 135 | 80.8 | 0.730 | 0.433 – 1.232 | 0.239 |
| | | 65-74 years | 13; 81 | 86.2 | 1.078 | 0.544 – 2.138 | 0.829 |
| | | ≥ 75 years | 2; 12 | 85.7 | 1.038 | 0.223 – 4.835 | 0.962 |
| | Owner gender (*n* = 895) | Female | 145; 666 | 82.1 | | Ref | |
| | | Male | 11; 73 | 86.9 | 1.445 | 0.748 – 2.792 | 0.274 |
| | Child ≤ 16 years in household (in 2020) (*n* = 895) | No | 114; 539 | 82.5 | | Ref | |
| | | Yes | 43; 199 | 82.2 | 0.979 | 0.665 – 1.441 | 0.914 |
| | Employed in animal care/veterinary sector (*n* = 896) | No | 147; 684 | 82.3 | | Ref | |
| | | Yes | 10; 55 | 84.6 | 1.182 | 0.589 – 2.373 | 0.638 |
| | Another dog(s) in household (in 2020) (*n* = 886) | No | 110; 540 | 83.1 | | Ref | |
| | | Yes | 43; 193 | 81.8 | 0.914 | 0.620 – 1.349 | 0.652 |

**Table S4. Contd…**

| | Variable | Category | *n* (reward only; mixed) | % (mixed) | Odds Ratio | 95% CI* | *p*-Value |
|---|---|---|---|---|---|---|---|
| **Owner/ household demographics** | Current working location of owner (*n* = 848) | At home | 28; 169 | 85.8 | 1.360 | 0.818 – 2.260 | 0.235 |
| | | Away from home | 35; 142 | 80.2 | 0.914 | 0.563 – 1.484 | 0.717 |
| | | At and away from home | 48; 213 | 81.6 | | Ref | |
| | | Unemployed | 5; 22 | 81.5 | 0.992 | 0.357 – 2.751 | 0.987 |
| | | Retired | 33; 153 | 82.3 | 1.045 | 0.640 – 1.705 | 0.861 |
| | Current working location of other household members (*n* = 741) | At home | 24; 119 | 83.2 | 0.887 | 0.501 – 1.570 | 0.681 |
| | | Away from home | 51; 193 | 83.2 | 0.677 | 0.420 – 1.092 | 0.110 |
| | | At and away from home | 34; 190 | 84.8 | | Ref | |
| | | Unemployed | 1; 9 | 90.0 | 1.611 | 0.198 – 13.125 | 0.656 |
| | | Retired | 18; 102 | 85.0 | 1.014 | 0.546 – 1.885 | 0.965 |
| | Owner lives alone/only adult in the household (*n* = 849) | No | 126; 609 | 82.9 | | Ref | |
| | | Yes | 23; 91 | 79.8 | 0.819 | 0.498 – 1.344 | 0.429 |
| **Dog demographics** | Dog sex (*n* = 895) | Female | 84; 345 | 80.4 | | Ref | |
| | | Male | 73; 393 | 84.3 | 1.311 | 0.928 – 1.851 | 0.125 |
| | Neutered (*n* = 896) | No | 69; 301 | 81.4 | | Ref | |
| | | Yes | 88; 438 | 83.3 | 1.141 | 0.806 – 1.615 | 0.457 |
| | Breed purity designation (*n* = 896) | Crossbred | 6; 33 | 84.6 | | Ref | |
| | | Purebred | 104; 514 | 83.2 | 0.899 | 0.367 – 2.199 | 0.815 |
| | | Designer Crossbred | 47; 192 | 80.3 | 0.743 | 0.294 – 1.876 | 0.529 |
| | Top 12 most common breeds at 21 months (*n* = 896) | Crossbred | 6; 33 | 84.6 | | Ref | |
| | | Cockapoo | 15; 73 | 83.0 | 0.885 | 0.315 – 2.484 | 0.816 |
| | | Labradoodle | 5; 20 | 80.0 | 0.727 | 0.196 – 2.696 | 0.634 |
| | | Cavapoo | 3; 13 | 81.3 | 0.788 | 0.171 – 3.629 | 0.760 |
| | | Labrador Retriever | 9; 88 | 90.7 | 1.778 | 0.587 – 5.382 | 0.309 |
| | | Cocker Spaniel | 11; 52 | 82.5 | 0.860 | 0.290 – 2.547 | 0.785 |
| | | Border Collie | 2; 29 | 93.5 | 2.636 | 0.493 – 14.093 | 0.257 |
| | | Miniature Smooth-Haired Dachshund | 8; 18 | 69.2 | 0.409 | 0.123 – 1.364 | 0.146 |
| | | Border Terrier | 2; 23 | 92.0 | 2.091 | 0.387 – 11.292 | 0.391 |
| | | Golden Retriever | 3; 24 | 88.9 | 1.455 | 0.330 – 6.404 | 0.620 |
| | | Whippet | 6; 13 | 68.4 | 0.394 | 0.107 – 1.447 | 0.160 |
| | | English Springer Spaniel | 1; 19 | 95.0 | 3.455 | 0.386 – 30.897 | 0.267 |
| | | Other | 86; 334 | 79.5 | 0.706 | 0.287 – 1.740 | 0.449 |

**Table S4. Contd…**

| | Variable | Category | *n* (reward only; mixed) | % (mixed) | Odds Ratio | 95% CI* | *p*-Value |
|---|---|---|---|---|---|---|---|
| **Dog demographics** | Breed group (*n* = 896) | Not KC recognised | 55; 231 | 80.8 | | Ref | |
| | | **Gundog** | **31; 235** | **88.3** | **1.805** | **1.121 – 2.906** | **0.015** |
| | | Hound | 20; 62 | 75.6 | 0.738 | 0.412 – 1.323 | 0.308 |
| | | **Pastoral** | **5; 58** | **92.1** | **2.762** | **1.058 – 7.211** | **0.038** |
| | | Terrier | 15; 63 | 80.8 | 1.000 | 0.530 – 1.888 | 1.000 |
| | | Toy | 8; 16 | 66.7 | 0.476 | 0.194 – 1.169 | 0.105 |
| | | Utility | 13; 50 | 79.4 | 0.916 | 0.465 – 1.803 | 0.799 |
| | | Working | 10; 24 | 70.6 | 0.571 | 0.258 – 1.264 | 0.167 |
| | Typical adult bodyweight (*n* = 863) | ≤ 10 kg | 43; 128 | 74.9 | 0.671 | 0.432 – 1.041 | 0.075 |
| | | 10 to < 20 kg | 64; 284 | 81.6 | | Ref | |
| | | 20 to < 30 kg | 29; 171 | 85.5 | 1.329 | 0.824 – 2.143 | 0.244 |
| | | **30 to < 40 kg** | **13; 115** | **89.8** | **1.993** | **1.057 – 3.759** | **0.033** |
| | | ≥ 40 kg | 4; 12 | 75.0 | 0.676 | 0.211 – 2.164 | 0.510 |
| **Purchase motivations for a dog/for breed chosen** | Dog to improve mental health (*n* = 896) | No | 87; 363 | 80.7 | | Ref | |
| | | Yes | 70; 376 | 84.3 | 1.287 | 0.911 – 1.820 | 0.153 |
| | Breed size suited to my lifestyle (*n* = 896) | No | 44; 234 | 84.2 | | Ref | |
| | | Yes | 113; 505 | 81.7 | 0.840 | 0.574 – 1.230 | 0.371 |
| | Breed good with children (*n* = 896) | No | 78; 413 | 84.1 | | Ref | |
| | | Yes | 79; 326 | 80.5 | 0.779 | 0.552 – 1.100 | 0.157 |
| | Breed easy to train (*n* = 896) | No | 97; 372 | 79.3 | | Ref | |
| | | **Yes** | **60; 367** | **85.9** | **1.595** | **1.121 – 2.270** | **0.009** |
| | Friends or family own this breed/crossbreed (*n* = 896) | No | 120; 562 | 82.4 | | Ref | |
| | | Yes | 37; 177 | 82.7 | 1.021 | 0.681 – 1.532 | 0.918 |
| | Respondent driving force to get a dog (*n* = 878) | No | 86; 428 | 83.3 | | Ref | |
| | | Yes | 69; 295 | 81.0 | 0.859 | 0.606 – 1.219 | 0.395 |
| | Had more time for a dog due to COVID-19 pandemic (*n* = 372) | No | 10; 39 | 79.6 | | Ref | |
| | | Yes | 54; 269 | 83.3 | 1.277 | 0.601 – 2.714 | 0.524 |
| **Pre-purchase research** | Research conducted (*n* = 895) | No | 59; 312 | 84.1 | | Ref | |
| | | Yes | 98; 426 | 81.3 | 0.822 | 0.577 – 1.172 | 0.278 |
| **Purchase behaviours** | Via animal-specific selling website (*n* = 895) | No | 81; 362 | 81.7 | | Ref | |
| | | Yes | 76; 376 | 83.2 | 1.107 | 0.784 – 1.563 | 0.563 |
| | Viewed in person prior to purchase (*n* = 896) | No | 53; 219 | 80.5 | | Ref | |
| | | Yes | 104; 520 | 83.3 | 1.210 | 0.839 – 1.746 | 0.308 |

**Table S4.** **Contd…**

| | Variable | Category | *n* (reward only; mixed) | % (mixed) | Odds Ratio | 95% CI* | *p*-Value |
|---|---|---|---|---|---|---|---|
| **Potentially illegal sales indicators** | Sold without dam present (*n* = 895) | No | 127; 577 | 82.0 | | Ref | |
| | | Yes | 30; 161 | 84.3 | 1.181 | 0.765 – 1.824 | 0.453 |
| | Sold outside breeders' home (*n* = 896) | No | 109; 452 | 80.6 | | Ref | |
| | | Yes | 48; 287 | 85.7 | 1.442 | 0.996 – 2.088 | 0.053 |
| | Sold without a microchip (*n* = 879) | No | 150; 691 | 82.2 | | Ref | |
| | | Yes | 5; 33 | 86.8 | 1.433 | 0.550 – 3.731 | 0.461 |
| | Sold < 6 weeks of age (*n* = 895) | No | 157; 736 | 82.4 | | Ref | |
| | | Yes | 0; 2 | 100.0 | ~ | ~ | ~ |
| | Sold with passport < 13 weeks of age (*n* = 812) | No | 132; 633 | 82.7 | | Ref | |
| | | Yes | 9; 38 | 80.9 | 0.880 | 0.416 – 1.865 | 0.740 |
| **Owner expectations** | Behaviour (*n* = 860) | As expected/better | 135; 593 | 81.5 | | Ref | |
| | | Worse | 17; 115 | 87.1 | 1.540 | 0.895 – 2.649 | 0.119 |
| | Training/maintaining obedience (*n* = 889) | As expected/easier | 118; 471 | 80.0 | | Ref | |
| | | **Harder** | **38; 262** | **87.3** | **1.727** | **1.163 – 2.565** | **0.007** |
| **Socialisation < 16 weeks** | Attended puppy classes (*n* = 895) | No | 82; 431 | 84.0 | | Ref | |
| | | Yes, in person | 52; 260 | 83.3 | 0.951 | 0.651 – 1.391 | 0.797 |
| | | **Yes, online** | **23; 47** | **67.1** | **0.389** | **0.224 – 0.675** | **< 0.001** |
| **Adult management** | Attended adult classes (*n* = 896) | No | 96; 395 | 80.4 | | Ref | |
| | | Yes, in person | 54; 315 | 85.4 | 1.418 | 0.984 – 2.042 | 0.061 |
| | | Yes, online | 7; 29 | 80.6 | 1.007 | 0.428 – 2.367 | 0.987 |
| | Exercise frequency (walks) (*n* = 860) | Never | 0; 1 | 100.0 | ~ | ~ | ~ |
| | | < Once a week | 0; 3 | 100.0 | ~ | ~ | ~ |
| | | Once a week | 1; 2 | 66.7 | 0.426 | 0.038 – 4.764 | 0.488 |
| | | Several times per week | 9; 36 | 80.0 | 0.852 | 0.392 – 1.850 | 0.685 |
| | | Once per day | 43; 186 | 81.2 | 0.921 | 0.604 – 1.404 | 0.703 |
| | | Twice per day | 69; 324 | 82.4 | | Ref | |
| | | > Twice per day | 31; 155 | 83.3 | 1.065 | 0.669 – 1.695 | 0.791 |
| | Exercise frequency (off lead) (*n* = 858) | Never | 12; 38 | 76.0 | 0.619 | 0.299 – 1.280 | 0.196 |
| | | < Once a week | 3; 26 | 89.7 | 1.694 | 0.491 – 5.848 | 0.404 |
| | | Once a week | 3; 24 | 88.9 | 1.564 | 0.451 – 5.425 | 0.481 |
| | | Several times per week | 26; 115 | 81.6 | 0.865 | 0.506 – 1.478 | 0.595 |
| | | Once per day | 43; 220 | 83.7 | | Ref | |
| | | Twice per day | 41; 178 | 81.3 | 0.849 | 0.530 – 1.359 | 0.495 |
| | | > Twice per day | 25; 104 | 80.6 | 0.813 | 0.471 – 1.403 | 0.457 |

**Table S4.** **Contd…**

| | Variable | Category | *n* (reward only; mixed) | % (mixed) | Odds Ratio | 95% CI* | *p*-Value |
|---|---|---|---|---|---|---|---|
| **Adult management** | Time left alone (*n* = 869) | Never | 17; 83 | 83.0 | | Ref | |
| | | Up to 1 hour | 36; 121 | 77.1 | 0.688 | 0.363 – 1.307 | 0.254 |
| | | 1 to 4 hours | 91; 411 | 81.9 | 0.925 | 0.524 – 1.634 | 0.788 |
| | | Over 4 hours | 10; 100 | 90.9 | 2.048 | 0.890 – 4.714 | 0.092 |
| | Location left alone (*n* = 833) | **A crate/indoor kennel (door closed)** | **16; 114** | **87.7** | **1.800** | **1.018 – 3.184** | **0.043** |
| | | Loose in a single room | 37; 197 | 84.2 | 1.345 | 0.866 – 2.043 | 0.164 |
| | | Loose in multiple rooms | 94; 372 | 79.8 | | Ref | |
| | | Outdoor kennel (door closed) | 0; 1 | 100.0 | ~ | ~ | ~ |
| | | Outdoor kennel (door open) | 0; 1 | 100.0 | ~ | ~ | ~ |
| | | Loose in an enclosed outdoor space | 0; 1 | 100.0 | ~ | ~ | ~ |
| | Change in time left alone from < 16 weeks (*n* = 840) | Same amount of time | 67; 282 | 80.8 | | Ref | |
| | | Less time | 4; 23 | 85.2 | 1.366 | 0.457 – 4.082 | 0.576 |
| | | More time ≤ 4 hours | 66; 298 | 81.9 | 1.073 | 0.736 – 1.564 | 0.715 |
| | | **More time > 4 hours** | **10; 90** | **90.0** | **2.138** | **1.056 – 4.330** | **0.035** |
| | General advice source for training and behaviour (*n* = 864) | Non-professional | 48; 204 | 81.0 | | Ref | |
| | | Professional | 107; 505 | 82.5 | 1.111 | 0.761 – 1.620 | 0.586 |
| | Respondent primary carer for dog (*n* = 895) | Shares care | 60; 328 | 84.5 | | Ref | |
| | | No | 1; 3 | 75.0 | 0.549 | 0.056 – 5.364 | 0.606 |
| | | Yes | 95; 408 | 81.1 | 0.786 | 0.551 – 1.120 | 0.182 |
| **Dog behaviour** | Total number of owner-reported undesirable behaviours (*n* = 762) | / / | / / | / / | **1.217** | **1.129 – 1.311** | **< 0.001** |
| | Control behaviour score (*n* = 854) | 0 | 45; 89 | 66.4 | | Ref | |
| | | **1+** | **107; 613** | **85.1** | **2.897** | **1.916 – 4.379** | **< 0.001** |
| | Attention seeking behaviour score (*n* = 812) | 0 | 45; 137 | 75.3 | | Ref | |
| | | **1+** | **97; 533** | **84.6** | **1.805** | **1.210 – 2.693** | **0.004** |
| | Aggressive behaviour score (*n* = 866) | 0 | 127; 523 | 80.5 | | Ref | |
| | | **1+** | **25; 191** | **88.4** | **1.855** | **1.171 – 2.938** | **0.008** |
| | Fear/avoidance behaviour score (*n* = 861) | 0 | 98; 402 | 80.4 | | Ref | |
| | | 1+ | 54; 307 | 85.0 | 1.386 | 0.963 – 1.994 | 0.079 |
| | Reaction to familiar people (*n* = 868) | 0 | 151; 667 | 81.5 | | Ref | |
| | | **1+** | **2; 48** | **96.0** | **5.433** | **1.306 – 22.601** | **0.020** |

**Table S4. Contd…**

| | Variable | Category | *n* (reward only; mixed) | % (mixed) | Odds Ratio | 95% CI* | *p*-Value |
|---|---|---|---|---|---|---|---|
| **Dog behaviour** | Reaction to unfamiliar people | 0 | 134; 555 | 80.6 | | Ref | |
| | (*n* = 872) | **1+** | **20; 163** | **89.1** | **1.968** | **1.192 – 3.248** | **0.008** |
| | Reaction to other dogs | 0 | 131; 547 | 80.7 | | Ref | |
| | (*n* = 869) | **1+** | **23; 168** | **88.0** | **1.749** | **1.087 – 2.815** | **0.021** |
| | SRB case | No | 998; 427 | 81.3 | | Ref | |
| | (*n* = 766) | Yes | 39; 202 | 83.8 | 1.189 | 0.791 – 1.786 | 0.405 |

## Supplementary Table S5: Owner sources of advice for general training and behaviour queries, descriptive and univariate logistic regression analysis

**Table S5.** **Descriptive and univariable analysis of categorical variables and owner sources of advice for their dog's behaviour and training queries during the first 21 months.** Descriptive and univariable binary logistic regression analysis of dogs whose owners responded to the question "*Where have you sought information about any aspect of your dog's general behaviour or training since the last survey?*". Responses were categorised as a binary outcome as to whether they sought professional advice where "*A veterinary professional (e.g., veterinary surgeon, veterinary nurse)*", "*Dog trainer*" or "*Dog behaviourist*" were coded separately from all other advice sources, which were coded together as one outcome along with four additional sources from qualitative content analysis of free-text responses. Owners who had answered "*I can't remember*" were reclassified as missing data. *95% confidence interval. Significant results are emboldened. ~ No analysis possible.

| | Variable | Category | *n* (professional; other) | % (professional) | Odds Ratio | 95% CI* | *p*-Value |
|---|---|---|---|---|---|---|---|
| **Owner/ household demographics** | First-time dog owner (*n* = 890) | No | 359; 189 | 65.5 | | Ref | |
| | | **Yes** | **267; 75** | **78.1** | **1.874** | **1.373 – 2.558** | **< 0.001** |
| | Owner age (*n* = 889) | 18-24 years | 30; 7 | 81.1 | 1.661 | 0.697 – 3.959 | 0.252 |
| | | 25-34 years | 125; 44 | 74.0 | 1.101 | 0.708 – 1.713 | 0.669 |
| | | 35-44 years | 121; 44 | 73.3 | 1.066 | 0.684 – 1.660 | 0.777 |
| | | 45-54 years | 178; 69 | 72.1 | | Ref | |
| | | 55-64 years | 109; 53 | 67.3 | 0.797 | 0.519 – 1.226 | 0.302 |
| | | **65-74 years** | **53; 43** | **55.2** | **0.478** | **0.293 – 0.779** | **0.003** |
| | | ≥ 75 years | 9; 4 | 69.2 | 0.872 | 0.260 – 2.925 | 0.825 |
| | Owner gender (*n* = 890) | Female | 575; 233 | 71.2 | | Ref | |
| | | Male | 51; 31 | 62.2 | 0.667 | 0.416 – 1.068 | 0.092 |
| | Child ≤ 16 years in household (in 2020; *n* = 890) | No | 458; 193 | 70.4 | | Ref | |
| | | Yes | 168; 71 | 70.3 | 0.997 | 0.721 – 1.379 | 0.986 |
| | Employed in animal care/veterinary sector (*n* = 891) | No | 579; 250 | 69.8 | | Ref | |
| | | Yes | 48; 14 | 77.4 | 1.480 | 0.802 – 2.734 | 0.210 |
| | Another dog(s) in household (in 2020) (*n* = 881) | No | 465; 174 | 72.8 | | Ref | |
| | | **Yes** | **157; 85** | **64.9** | **0.691** | **0.504 – 0.948** | **0.022** |

**Table S5.** **Contd…**

| | Variable | Category | *n* (professional; other) | % (professional) | Odds Ratio | 95% CI* | *p*-Value |
|---|---|---|---|---|---|---|---|
| **Owner/ household demographics** | Current working location of owner (*n* = 863) | At home | 149; 49 | 75.3 | 1.119 | 0.733 – 1.706 | 0.603 |
| | | Away from home | 126; 58 | 68.5 | 0.799 | 0.529 – 1.208 | 0.288 |
| | | At and away from home | 193; 71 | 73.1 | | Ref | |
| | | Unemployed | 23; 7 | 76.7 | 1.209 | 0.497 – 2.940 | 0.676 |
| | | **Retired** | **120; 67** | **64.2** | **0.659** | **0.440 – 0.987** | **0.043** |
| | Current working location of other household members (*n* = 751) | At home | 103; 39 | 72.5 | 0.886 | 0.551 – 1.424 | 0.616 |
| | | Away from home | 174; 73 | 70.4 | 0.799 | 0.533 – 1.199 | 0.279 |
| | | At and away from home | 170; 57 | 74.9 | | Ref | |
| | | Unemployed | 7; 3 | 70.0 | 0.782 | 0.196 – 3.127 | 0.728 |
| | | Retired | 83; 42 | 66.4 | 0.663 | 0.411 – 1.068 | 0.091 |
| | Owner lives alone/only adult in the household (*n* = 864) | No | 532; 213 | 71.4 | | Ref | |
| | | Yes | 79; 40 | 66.4 | 0.791 | 0.524 – 1.194 | 0.264 |
| **Dog demographics** | Dog sex (*n* = 890) | Female | 290; 131 | 68.9 | | Ref | |
| | | Male | 336; 28.4 | 71.6 | 1.141 | 0.856 – 1.522 | 0.369 |
| | Neutered (*n* = 891) | No | 247; 122 | 66.9 | | Ref | |
| | | Yes | 380; 142 | 72.8 | 1.322 | 0.898 – 1.767 | 0.060 |
| | Breed purity designation (*n* = 891) | Crossbred | 26; 12 | 68.4 | | Ref | |
| | | Purebred | 440; 176 | 71.4 | 1.154 | 0.570 – 2.042 | 0.691 |
| | | Designer Crossbred | 161; 76 | 67.9 | 0.978 | 0.468 – 2.042 | 0.952 |
| | Top 12 most common breeds at 21 months (*n* = 891) | Crossbred | 26; 12 | 68.4 | | Ref | |
| | | Cockapoo | 62; 22 | 73.8 | 1.301 | 0.562 – 3.011 | 0.539 |
| | | Labradoodle | 16; 10 | 61.5 | 0.738 | 0.260 – 2.100 | 0.570 |
| | | Cavapoo | 14; 4 | 77.8 | 1.615 | 0.438 – 5.956 | 0.471 |
| | | Labrador Retriever | 72; 23 | 75.8 | 1.445 | 0.630 – 3.312 | 0.385 |
| | | Cocker Spaniel | 44; 18 | 71.0 | 1.128 | 0.470 – 2.711 | 0.787 |
| | | Border Collie | 24; 8 | 75.0 | 1.385 | 0.483 – 3.967 | 0.545 |
| | | Miniature Smooth-Haired Dachshund | 19; 8 | 70.4 | 1.096 | 0.375 – 3.204 | 0.867 |
| | | Border Terrier | 16; 10 | 61.5 | 0.738 | 0.260 – 2.100 | 0.570 |
| | | Golden Retriever | 22; 5 | 81.5 | 2.031 | 0.619 – 6.660 | 0.242 |
| | | Whippet | 16; 5 | 76.2 | 1.477 | 0.438 – 4.978 | 0.529 |
| | | English Springer Spaniel | 18; 0 | 100.0 | ~ | ~ | ~ |
| | | Other | 278; 139 | 66.7 | 0.923 | 0.452 – 1.884 | 0.826 |

**Table S5.** **Contd…**

| | Variable | Category | *n* (professional; other) | % (professional) | Odds Ratio | 95% CI* | *p*-Value |
|---|---|---|---|---|---|---|---|
| **Dog demographics** | Breed group ($n$ = 891) | Not KC recognised | 195; 88 | 68.9 | | Ref | |
| | | **Gundog** | **199; 60** | **76.8** | **1.497** | **1.021 – 2.195** | **0.039** |
| | | Hound | 58; 26 | 69.0 | 1.007 | 0.595 – 1.705 | 0.980 |
| | | Pastoral | 44; 18 | 71.0 | 1.103 | 0.603 – 2.017 | 0.750 |
| | | Terrier | 54; 29 | 65.1 | 0.840 | 0.501 – 1.409 | 0.509 |
| | | Toy | 16; 8 | 66.7 | 0.903 | 0.372 – 2.187 | 0.820 |
| | | Utility | 39; 23 | 62.9 | 0.765 | 0.431 – 1.358 | 0.360 |
| | | Working | 22; 12 | 64.7 | 0.827 | 0.392 – 1.746 | 0.619 |
| | Typical adult bodyweight ($n$ = 860) | ≤ 10 kg | 123; 51 | 70.7 | 1.042 | 0.699 – 1.553 | 0.839 |
| | | 10 to < 20 kg | 243; 105 | 69.8 | | Ref | |
| | | 20 to < 30 kg | 146; 53 | 73.4 | 1.190 | 0.807 – 1.756 | 0.380 |
| | | 30 to < 40 kg | 89; 36 | 71.2 | 1.068 | 0.681 – 1.675 | 0.774 |
| | | ≥ 40 kg | 7; 7 | 50.0 | 0.432 | 0.148 – 1.263 | 0.125 |
| | Insured ($n$ = 891) | No | 76; 36 | 67.9 | | Ref | |
| | | Yes | 551; 228 | 70.7 | 1.145 | 0.748 – 1.752 | 0.534 |
| **Purchase motivations for a dog/for breed chosen** | Dog to improve mental health ($n$ = 891) | No | 307; 143 | 68.2 | | Ref | |
| | | Yes | 320; 121 | 72.6 | 1.232 | 0.923 – 1.644 | 0.156 |
| | Breed size suited to my lifestyle ($n$ = 891) | No | 190; 94 | 66.9 | | Ref | |
| | | Yes | 437; 170 | 72.0 | 1.272 | 0.938 – 1.724 | 0.121 |
| | Breed good with children ($n$ = 891) | No | 347; 142 | 71.0 | | Ref | |
| | | Yes | 280; 122 | 69.7 | 0.939 | 0.704 – 1.254 | 0.670 |
| | Breed easy to train ($n$ = 891) | No | 313; 153 | 67.2 | | Ref | |
| | | **Yes** | **314; 111** | **73.9** | **1.383** | **1.034 – 1.848** | **0.029** |
| | Friends or family own this breed/crossbreed ($n$ = 891) | No | 468; 210 | 69.0 | | Ref | |
| | | Yes | 159; 54 | 74.6 | 1.321 | 0.932 – 1.873 | 0.118 |
| | Respondent driving force to get a dog ($n$ = 873) | No | 353; 161 | 68.7 | | Ref | |
| | | Yes | 263; 96 | 73.3 | 1.249 | 0.927 – 1.685 | 0.144 |
| | Had more time for a dog due to COVID-19 pandemic ($n$ = 363) | No | 26; 24 | 52.0 | | Ref | |
| | | **Yes** | **243; 70** | **77.6** | **3.204** | **1.732 – 5.928** | **< 0.001** |
| **Pre-purchase research** | Research conducted ($n$ = 889) | No | 233; 142 | 62.1 | | Ref | |
| | | **Yes** | **392; 122** | **76.3** | **1.958** | **1.463 – 2.620** | **< 0.001** |

**Table S5.** **Contd…**

| | Variable | Category | *n* (professional; other) | % (professional) | Odds Ratio | 95% CI* | *p*-Value |
|---|---|---|---|---|---|---|---|
| **Purchase behaviours** | Via animal-specific selling website (*n* = 890) | No | 312; 123 | 71.7 | | Ref | |
| | | Yes | 314; 141 | 69.0 | 0.878 | 0.658 – 1.171 | 0.376 |
| | Viewed in person prior to purchase (*n* = 891) | No | 189; 84 | 69.2 | | Ref | |
| | | Yes | 438; 180 | 70.9 | 1.081 | 0.793 – 1.475 | 0.621 |
| **Potentially illegal sales indicators** | Sold without dam present (*n* = 889) | No | 481; 219 | 68.7 | | Ref | |
| | | **Yes** | **145; 44** | **76.7** | **1.500** | **1.033 – 2.179** | **0.033** |
| | Sold outside breeders' home (*n* = 891) | No | 392; 165 | 70.4 | | Ref | |
| | | Yes | 235; 99 | 70.4 | 0.999 | 0.742 – 1.345 | 0.996 |
| | Sold without a microchip (*n* = 874) | No | 593; 243 | 70.9 | | Ref | |
| | | Yes | 22; 16 | 57.9 | 0.563 | 0.291 – 1.091 | 0.089 |
| | Sold < 6 weeks of age (*n* = 890) | No | 624; 264 | 70.3 | | Ref | |
| | | Yes | 2; 0 | 100.0 | ~ | ~ | ~ |
| | Sold with passport < 13 weeks of age (*n* = 808) | No | 546; 215 | 71.1 | | Ref | |
| | | **Yes** | **27; 20** | **57.4** | **0.532** | **0.292 – 0.968** | **0.039** |
| **Owner expectations** | Behaviour (*n* = 876) | As expected/better | 500; 244 | 67.2 | | Ref | |
| | | **Worse** | **115; 17** | **87.1** | **3.301** | **1.940 – 5.619** | **< 0.001** |
| | Training/maintaining obedience (*n* = 886) | As expected/easier | 371; 219 | 62.9 | | Ref | |
| | | **Harder** | **252; 44** | **85.1** | **3.381** | **2.356 – 4.851** | **< 0.001** |
| **Socialisation < 16 weeks** | Attended puppy classes (*n* = 890) | No | 311; 202 | 60.6 | | Ref | |
| | | **Yes, in person** | **255; 52** | **83.1** | **3.185** | **2.252 – 4.506** | **< 0.001** |
| | | **Yes, online** | **61; 9** | **87.1** | **4.402** | **2.139 – 9.062** | **< 0.001** |
| **Adult management** | Attended adult classes (*n* = 891) | No | 262; 228 | 53.5 | | Ref | |
| | | **Yes, in person** | **332; 34** | **90.7** | **8.498** | **5.724 – 12.614** | **< 0.001** |
| | | **Yes, online** | **33; 2** | **94.3** | **14.359** | **3.408 – 60.500** | **< 0.001** |
| | Owner training method (*n* = 864) | Rewards-based only | 107; 48 | 69.0 | | Ref | |
| | | Rewards-based & 1 aversive | 125; 56 | 69.1 | 1.001 | 0.630 – 1.592 | 0.996 |
| | | Rewards-based & > 1 aversive | 612; 252 | 72.0 | 1.152 | 0.780 – 1.701 | 0.477 |
| | | Aversive only | 0; 0 | 0.0 | ~ | ~ | ~ |
| | Change in time left alone from < 16 weeks (*n* = 856) | Same amount of time | 233; 122 | 65.6 | | Ref | |
| | | Less time | 21; 6 | 77.8 | 1.833 | 0.721 – 4.660 | 0.203 |
| | | **More time ≤ 4 hours** | **272; 98** | **73.5** | **1.453** | **1.057 – 1.998** | **0.021** |
| | | **More time > 4 hours** | **79; 25** | **76.0** | **1.655** | **1.003 – 2.729** | **0.049** |

**Table S5. Contd…**

| | Variable | Category | n (professional; other) | % (professional) | Odds Ratio | 95% CI* | *p*-Value |
|---|---|---|---|---|---|---|---|
| **Dog behaviour** | Total number of owner-reported undesirable behaviours (*n* = 781) | / / | / / | / / | **1.162** | **1.097 – 1.230** | **< 0.001** |
| | Control behaviour score | 0 | 80; 55 | 59.3 | | Ref | |
| | (*n* = 868) | **1+** | **530; 203** | **72.3** | **1.795** | **1.228 – 2.623** | **0.003** |
| | Attention seeking behaviour score | 0 | 114; 74 | 60.6 | | Ref | |
| | (*n* = 829) | **1+** | **468; 173** | **73.0** | **1.756** | **1.249 – 2.469** | **0.001** |
| | Aggressive behaviour score | 0 | 447; 211 | 67.9 | | Ref | |
| | (*n* = 880) | **1+** | **173; 49** | **77.9** | **1.667** | **1.166 – 2.381** | **0.005** |
| | Fear/avoidance behaviour score | 0 | 342; 171 | 66.7 | | Ref | |
| | (*n* = 875) | **1+** | **277; 85** | **76.5** | **1.629** | **1.202 – 2.210** | **0.002** |
| | Reaction to familiar people | 0 | 582; 247 | 70.2 | | Ref | |
| | (*n* = 881) | 1+ | 41; 11 | 78.8 | 1.582 | 0.800 – 3.129 | 0.188 |
| | Reaction to unfamiliar people | 0 | 484; 216 | 69.1 | | Ref | |
| | (*n* = 886) | 1+ | 141; 45 | 75.8 | 1.398 | 0.964 – 2.028 | 0.077 |
| | Reaction to other dogs | 0 | 472; 220 | 68.2 | | Ref | |
| | (*n* = 882) | **1+** | **149; 41** | **78.4** | **1.694** | **1.157 – 2.479** | **0.007** |
| | SRB case | No | 364; 176 | 67.4 | | Ref | |
| | (*n* = 778) | **Yes** | **180; 58** | **75.6** | **1.501** | **1.061 – 2.122** | **0.022** |

## Supplementary Table S6: Owner expectations of behaviour, descriptive and univariate logistic regression analysis

**Table S6. Descriptive and univariable analysis of categorical variables and owner expectations of their dog's behaviour at acquisition versus at 21 months.**

Descriptive and univariable binary logistic regression analysis of dogs whose owners responded to the question "*Compared to my expectations when I first acquired my dog, their behaviour since the last survey (in November/December 2020) has been...*". Responses were categorised as a binary outcome as to whether expectations had been met where "*Worse than expected*" were coded separately from "*As expected*" and "*Better than expected*", which were coded together as one outcome. Owners who had answered "*I'm not sure*" were reclassified as missing data. *95% confidence interval. Significant results are emboldened. ~ No analysis possible.

| | Variable | Category | *n* (worse; better/as) | % (worse) | Odds Ratio | 95% CI* | *p*-Value |
|---|---|---|---|---|---|---|---|
| **Owner/ household demographics** | First-time dog owner (*n* = 886) | No | 72; 474 | 13.2 | | Ref | |
| | | Yes | 60; 280 | 17.6 | 1.411 | 0.971 – 2.049 | 0.071 |
| | Owner age (*n* = 885) | 18-24 years | 8; 29 | 21.6 | 1.986 | 0.831 – 4.745 | 0.123 |
| | | **25-34 years** | **36; 131** | **21.6** | **1.979** | **1.164 – 3.365** | **0.012** |
| | | 35-44 years | 20; 145 | 12.1 | 0.993 | 0.543 – 1.816 | 0.982 |
| | | 45-54 years | 30; 216 | 12.2 | | Ref | |
| | | 55-64 years | 26; 138 | 15.9 | 1.357 | 0.770 – 2.391 | 0.292 |
| | | 65-74 years | 8; 85 | 8.6 | 0.678 | 0.299 – 1.537 | 0.352 |
| | | ≥ 75 years | 4; 9 | 30.8 | 3.200 | 0.928 – 11.038 | 0.066 |
| | Owner gender (*n* = 886) | Female | 122; 682 | 15.2 | | Ref | |
| | | Male | 10; 72 | 12.2 | 0.776 | 0.390 – 1.546 | 0.472 |
| | Child ≤ 16 years in household (in 2020; *n* = 886) | No | 100; 548 | 15.4 | | Ref | |
| | | Yes | 31; 207 | 13.0 | 0.821 | 0.532 – 1.266 | 0.372 |
| | Employed in animal care/veterinary sector (*n* = 887) | No | 123; 702 | 14.9 | | Ref | |
| | | Yes | 9; 53 | 14.5 | 0.969 | 0.466 – 2.015 | 0.933 |
| | Another dog(s) in household (in 2020) (*n* = 877) | No | 103; 532 | 16.2 | | Ref | |
| | | Yes | 29; 213 | 12.0 | 0.703 | 0.452- 1.094 | 0.118 |
| | Current working location of owner (*n* = 864) | At home | 30; 169 | 15.1 | 1.094 | 0.650 – 1.842 | 0.736 |
| | | Away from home | 24; 158 | 13.2 | 0.936 | 0.539 – 1.626 | 0.815 |
| | | At and away from home | 37; 228 | 14.0 | | Ref | |
| | | Unemployed | 7; 23 | 23.3 | 1.875 | 0.751 – 4.681 | 0.178 |
| | | Retired | 31; 157 | 16.5 | 1.217 | 0.724 – 2.044 | 0.459 |

**Table S6.** **Contd…**

| | Variable | Category | *n* (worse; better/as) | % (worse) | Odds Ratio | 95% CI* | *p*-Value |
|---|---|---|---|---|---|---|---|
| **Owner/ household demographics** | Current working location of other household members (*n* = 755) | At home | 21; 124 | 14.5 | 0.792 | 0.446 – 1.407 | 0.426 |
| | | Away from home | 31; 216 | 12.6 | 0.671 | 0.404 – 1.115 | 0.124 |
| | | At and away from home | 40; 187 | 17.6 | | Ref | |
| | | Unemployed | 2; 8 | 20.0 | 1.169 | 0.239 – 5.712 | 0.847 |
| | | Retired | 18; 108 | 14.3 | 0.779 | 0.426 – 1.426 | 0.419 |
| | Owner lives alone/only adult in the household (*n* = 865) | No | 109; 640 | 14.6 | | Ref | |
| | | Yes | 20; 96 | 17.2 | 1.223 | 0.725 – 2.063 | 0.450 |
| **Dog demographics** | Dog sex (*n* = 886) | Female | 50; 365 | 12.0 | | Ref | |
| | | **Male** | **82; 289** | **17.4** | **1.539** | **1.053 – 2.249** | **0.026** |
| | Neutered (*n* = 887) | No | 49; 318 | 13.4 | | Ref | |
| | | Yes | 83; 437 | 16.0 | 1.233 | 0.842 – 1.805 | 0.283 |
| | Breed purity designation (*n* = 887) | Crossbred | 8; 31 | 20.5 | | Ref | |
| | | Purebred | 93; 516 | 15.3 | 0.698 | 0.311 – 1.567 | 0.384 |
| | | Designer Crossbred | 31; 208 | 13.0 | 0.578 | 0.243 – 1.370 | 0.213 |
| | Top 12 most common breeds at 21 months (*n* = 887) | Crossbred | 8; 31 | 20.5 | | Ref | |
| | | Cockapoo | 10; 74 | 11.9 | 0.524 | 0.189 – 1.452 | 0.214 |
| | | Labradoodle | 5; 21 | 19.2 | 0.923 | 0.265 – 3.211 | 0.899 |
| | | Cavapoo | 1; 17 | 5.6 | 0.228 | 0.026 – 1.979 | 0.180 |
| | | Labrador Retriever | 13; 82 | 13.7 | 0.614 | 0.232 – 1.625 | 0.326 |
| | | **Cocker Spaniel** | **4; 57** | **6.6** | **0.272** | **0.076 – 0.976** | **0.046** |
| | | Border Collie | 8; 24 | 25.0 | 1.292 | 0.423 – 3.941 | 0.653 |
| | | Miniature Smooth-Haired Dachshund | 5; 21 | 19.2 | 0.923 | 0.265 – 3.211 | 0.899 |
| | | Border Terrier | 2; 23 | 8.0 | 0.337 | 0.065 – 1.738 | 0.194 |
| | | Golden Retriever | 4; 24 | 14.3 | 0.646 | 0.174 – 2.401 | 0.514 |
| | | Whippet | 1; 20 | 4.8 | 0.194 | 0.022 – 1.669 | 0.135 |
| | | English Springer Spaniel | 2; 18 | 10.0 | 0.431 | 0.082 – 2.253 | 0.318 |
| | | Other | 69; 343 | 16.7 | 0.780 | 0.344 – 1.768 | 0.551 |

**Table S6.** **Contd…**

| | Variable | Category | *n* (worse; better/as) | % (worse) | Odds Ratio | 95% CI* | *p*-Value |
|---|---|---|---|---|---|---|---|
| **Dog demographics** | Breed group (*n* = 887) | Not KC recognised | 44; 244 | 14.7 | | Ref | |
| | | Gundog | 30; 230 | 11.5 | 0.758 | 0.459 – 1.252 | 0.279 |
| | | Hound | 13; 68 | 16.0 | 1.111 | 0.564 – 2.187 | 0.761 |
| | | Pastoral | 15; 48 | 23.8 | 1.815 | 0.933 – 3.533 | 0.079 |
| | | Terrier | 13; 67 | 16.3 | 1.127 | 0.572 – 2.221 | 0.729 |
| | | Toy | 5; 19 | 20.8 | 1.529 | 0.541 – 4.317 | 0.423 |
| | | Utility | 8; 51 | 13.6 | 0.911 | 0.404 – 2.057 | 0.823 |
| | | Working | 6; 28 | 17.6 | 1.245 | 0.486 – 3.189 | 0.648 |
| | Typical adult bodyweight (*n* = 853) | ≤ 10 kg | 28; 138 | 16.9 | 1.371 | 0.821 – 2.289 | 0.228 |
| | | 10 to < 20 kg | 45; 304 | 12.9 | | Ref | |
| | | 20 to < 30 kg | 28; 170 | 14.1 | 1.113 | 0.670 – 1.849 | 0.680 |
| | | 30 to < 40 kg | 21; 105 | 16.7 | 1.351 | 0.769 – 2.374 | 0.295 |
| | | **≥ 40 kg** | **5; 9** | **35.7** | **3.753** | **1.204 – 11.702** | **0.023** |
| **Purchase motivations for a dog/for breed chosen** | Dog to improve mental health (*n* = 887) | No | 63; 384 | 14.1 | | Ref | |
| | | Yes | 69; 371 | 15.7 | 1.134 | 0.783 – 1.641 | 0.507 |
| | Breed size suited to my lifestyle (*n* = 887) | No | 42; 240 | 14.9 | | Ref | |
| | | Yes | 90; 515 | 14.9 | 0.999 | 0.671 – 1.485 | 0.995 |
| | Breed good with children (*n* = 887) | No | 76; 411 | 15.6 | | Ref | |
| | | Yes | 56; 344 | 14.0 | 0.880 | 0.606 – 1.279 | 0.504 |
| | Breed easy to train (*n* = 887) | No | 67; 394 | 14.5 | | Ref | |
| | | Yes | 65; 361 | 15.3 | 1.059 | 0.731 – 1.533 | 0.762 |
| | Friends or family own this breed/crossbreed (*n* = 887) | No | 103; 571 | 15.3 | | Ref | |
| | | Yes | 29; 184 | 13.6 | 0.874 | 0.560 – 1.362 | 0.552 |
| | Respondent driving force to get a dog (*n* = 869) | No | 69; 444 | 13.5 | | Ref | |
| | | Yes | 61; 295 | 17.1 | 1.331 | 0.915 – 1.935 | 0.135 |
| | Had more time for a dog due to COVID-19 pandemic (*n* = 361) | No | 8; 41 | 16.3 | | Ref | |
| | | Yes | 53; 259 | 17.0 | 1.049 | 0.465 – 2.365 | 0.909 |
| **Pre-purchase research** | Research conducted (*n* = 885) | No | 46; 327 | 12.3 | | Ref | |
| | | Yes | 86; 426 | 16.8 | 1.435 | 0.976 – 2.111 | 0.067 |
| **Purchase behaviours** | Via animal-specific selling website (*n* = 886) | No | 64; 368 | 14.8 | | Ref | |
| | | Yes | 68; 386 | 15.0 | 1.013 | 0.700 – 1.466 | 0.946 |
| | Viewed in person prior to purchase (*n* = 887) | No | 35; 235 | 13.0 | | Ref | |
| | | Yes | 97; 520 | 15.7 | 1.252 | 0.826 – 1.899 | 0.289 |

**Table S6. Contd…**

| | Variable | Category | *n* (worse; better/as) | % (worse) | Odds Ratio | 95% CI* | *p*-Value |
|---|---|---|---|---|---|---|---|
| **Potentially illegal sales indicators** | Sold without dam present (*n* = 885) | No | 97; 599 | 13.9 | | Ref | |
| | | Yes | 35; 154 | 18.5 | 1.403 | 0.917 – 2.147 | 0.118 |
| | Sold outside breeders' home (*n* = 887) | No | 77; 480 | 13.8 | | Ref | |
| | | Yes | 55; 275 | 16.7 | 1.247 | 0.856 – 1.817 | 0.251 |
| | Sold without a microchip (*n* = 870) | No | 121; 710 | 14.6 | | Ref | |
| | | Yes | 8; 31 | 20.5 | 1.514 | 0.680 – 3.373 | 0.310 |
| | Sold < 6 weeks of age (*n* = 886) | No | 132; 752 | 14.9 | | Ref | |
| | | Yes | 0; 2 | 0.0 | ~ | ~ | ~ |
| | Sold with passport < 13 weeks of age (*n* = 804) | No | 115; 642 | 15.2 | | Ref | |
| | | Yes | 8; 39 | 17.0 | 1.145 | 0.522 – 2.514 | 0.735 |
| **Socialisation < 16 weeks** | Attended puppy classes (*n* = 886) | No | 71; 442 | 13.8 | | Ref | |
| | | Yes, in person | 50; 254 | 16.4 | 1.225 | 0.827 – 1.816 | 0.311 |
| | | Yes, online | 11; 58 | 15.9 | 1.181 | 0.591 – 2.358 | 0.638 |
| **Adult management** | Attended adult classes (*n* = 887) | No | 50; 439 | 10.2 | | Ref | |
| | | **Yes, in person** | **71; 292** | **19.6** | **2.135** | **1.444 – 3.156** | **< 0.001** |
| | | **Yes, online** | **11; 24** | **31.4** | **4.024** | **1.861 – 8.702** | **< 0.001** |
| | Owner training method (*n* = 860) | Rewards-based only | 17; 135 | 11.2 | | Ref | |
| | | Rewards-based & 1 aversive | 20; 160 | 11.1 | 0.993 | 0.500 – 1.971 | 0.983 |
| | | **Rewards-based & > 1 aversive** | **95; 433** | **18.0** | **1.742** | **1.004 – 3.023** | **0.048** |
| | | Aversive only | 0; 0 | 0.0 | ~ | ~ | ~ |
| | Exercise frequency (walks) (*n* = 876) | Never | 0; 1 | 0.0 | ~ | ~ | ~ |
| | | < Once a week | 1; 2 | 33.3 | 2.508 | 0.224 – 28.058 | 0.456 |
| | | Once a week | 0; 3 | 0.0 | ~ | ~ | ~ |
| | | Several times per week | 5; 42 | 10.6 | 0.597 | 0.228 – 1.566 | 0.294 |
| | | Once per day | 38; 196 | 16.2 | 0.972 | 0.628 – 1.504 | 0.900 |
| | | Twice per day | 66; 331 | 16.6 | | Ref | |
| | | **> Twice per day** | **20; 171** | **10.5** | **0.587** | **0.344 – 1.000** | **0.050** |
| | Exercise frequency (off lead) (*n* = 874) | Never | 11; 40 | 21.6 | 1.400 | 0.667 – 2.939 | 0.374 |
| | | < Once a week | 5; 24 | 17.2 | 1.061 | 0.384 – 2.930 | 0.910 |
| | | Once a week | 8; 19 | 29.6 | 2.144 | 0.883 – 5.204 | 0.092 |
| | | Several times per week | 28; 116 | 19.4 | 1.229 | 0.728 – 2.076 | 0.441 |
| | | Once per day | 44; 224 | 16.4 | | Ref | |
| | | Twice per day | 24; 198 | 10.8 | 0.617 | 0.362 – 1.051 | 0.076 |
| | | **> Twice per day** | **9; 124** | **6.8** | **0.370** | **0.175 – 0.782** | **0.009** |

**Table S6. Contd…**

| | Variable | Category | *n* (worse; better/as) | % (worse) | Odds Ratio | 95% CI* | *p*-Value |
|---|---|---|---|---|---|---|---|
| **Adult management** | Time left alone (*n* = 886) | Never | 24; 81 | 22.9 | | Ref | |
| | | Up to 1 hour | 32; 127 | 20.1 | 0.850 | 0.468 – 1.547 | 0.595 |
| | | **1 to 4 hours** | **65; 445** | **12.7** | **0.493** | **0.292 – 0.833** | **0.008** |
| | | **Over 4 hours** | **10; 102** | **8.9** | **0.331** | **0.150 – 0.731** | **0.006** |
| | Change in time left alone from < 16 weeks (*n* = 856) | Same amount of time | 57; 297 | 16.1 | | Ref | |
| | | **Less time** | **9; 18** | **33.3** | **2.605** | **1.115 – 6.088** | **0.027** |
| | | More time ≤ 4 hours | 51; 322 | 13.7 | 0.825 | 0.548 – 1.243 | 0.358 |
| | | More time > 4 hours | 12; 90 | 11.8 | 0.695 | 0.357 – 1.352 | 0.284 |
| | Location left alone (*n* = 849) | A crate/indoor kennel (door closed) | 22; 109 | 16.8 | 1.232 | 0.728 – 2.085 | 0.437 |
| | | Loose in a single room | 36; 203 | 15.1 | 1.083 | 0.698 – 1.679 | 0.723 |
| | | Loose in multiple rooms | 67; 409 | 14.1 | | Ref | |
| | | Outdoor kennel (door closed) | 0; 1 | 0.0 | ~ | ~ | ~ |
| | | Outdoor kennel (door open) | 0; 1 | 0.0 | ~ | ~ | ~ |
| | | Loose in an enclosed outdoor space | 0; 1 | 0.0 | ~ | ~ | ~ |
| | General advice source for training and behaviour (*n* = 876) | Non-professional | 17; 244 | 6.5 | | Ref | |
| | | **Professional** | **115; 500** | **18.7** | **3.301** | **1.940 – 5.619** | **< 0.001** |
| | Respondent primary carer for dog (*n* = 886) | Shares care | 50; 333 | 13.1 | | Ref | |
| | | No | 2; 2 | 50.0 | 6.660 | 0.917 – 48.352 | 0.061 |
| | | Yes | 80; 419 | 16.0 | 1.272 | 0.868 – 1.862 | 0.217 |
| **Dog behaviour** | Total number of owner-reported undesirable behaviours (*n* = 784) | / | / | / | **1.374** | **1.282 – 1.472** | **< 0.001** |
| | | / | / | / | | | |
| | Control behaviour score (*n* = 863) | 0 | 5; 132 | 3.6 | | Ref | |
| | | **1+** | **123; 603** | **16.9** | **5.385** | **2.159 – 13.429** | **< 0.001** |
| | Attention seeking behaviour score (*n* = 829) | 0 | 11; 178 | 5.8 | | Ref | |
| | | **1+** | **114; 526** | **17.8** | **3.507** | **1.846 – 6.663** | **< 0.001** |
| | Aggressive behaviour score (*n* = 874) | 0 | 59; 598 | 9.0 | | Ref | |
| | | **1+** | **72; 145** | **33.2** | **5.003** | **3.411 – 7.427** | **< 0.001** |
| | Fear/avoidance behaviour score (*n* = 870) | 0 | 47; 462 | 9.2 | | Ref | |
| | | **1+** | **82; 279** | **22.7** | **2.889** | **1.960 – 4.259** | **< 0.001** |
| | Reaction to familiar people (*n* = 876) | 0 | 107; 718 | 13.0 | | Ref | |
| | | **1+** | **24; 27** | **47.1** | **5.965** | **3.319 – 10.719** | **< 0.001** |
| | Reaction to unfamiliar people (*n* = 880) | 0 | 82; 612 | 11.8 | | Ref | |
| | | **1+** | **49; 137** | **26.3** | **2.669** | **1.790 – 3.980** | **< 0.001** |

**Table S6.** **Contd…**

| | Variable | Category | *n* (worse; better/as) | % (worse) | Odds Ratio | 95% CI* | *p*-Value |
|---|---|---|---|---|---|---|---|
| **Dog behaviour** | Reaction to other dogs | 0 | 63; 624 | 9.2 | | Ref | |
| | (*n* = 877) | **1+** | **67; 123** | **35.3** | **5.395** | **3.636 – 8.005** | **< 0.001** |
| | SRB case | No | 71; 469 | 13.1 | | Ref | |
| | (*n* = 781) | Yes | 42; 199 | 17.4 | 1.394 | 0.920 – 2.113 | 0.117 |

## Supplementary Table S7: Owner expectations of training, descriptive and univariate logistic regression analysis

**Table S7. Descriptive and univariable analysis of categorical variables and owner expectations of training and maintaining obedience training with their dog at acquisition versus at 21 months.**

Descriptive and univariable binary logistic regression analysis of dogs whose owners responded to the question "*Compared to my expectations when I first acquired my dog, training and ongoing maintenance of their basic obedience since the last survey (in November/December 2020) has been...*". Responses were categorised as a binary outcome as to whether expectations had been met where "*Harder than expected*" were coded separately from "*As expected*" and "*Easier than expected*", which were coded together as one outcome. Owners who had answered "*I'm not sure*" were reclassified as missing data. *95% confidence interval. Significant results are emboldened. ~ No analysis possible.

| | Variable | Category | *n* (harder; easier/as) | % (harder) | Odds Ratio | 95% CI* | *p*-Value |
|---|---|---|---|---|---|---|---|
| **Owner/ household demographics** | First-time dog owner (*n* = 917) | No | 162; 401 | 28.8 | | Ref | |
| | | **Yes** | **142; 212** | **40.1** | **1.658** | **1.253 – 2.194** | **< 0.001** |
| | Owner age (*n* = 916) | 18-24 years | 13; 24 | 35.1 | 1.096 | 0.532 – 2.261 | 0.804 |
| | | **25-34 years** | **76; 96** | **44.2** | **1.602** | **1.075 – 2.387** | **0.020** |
| | | 35-44 years | 45; 128 | 26.0 | 0.711 | 0.463 – 1.092 | 0.120 |
| | | 45-54 years | 84; 170 | 33.1 | | Ref | |
| | | 55-64 years | 53; 117 | 31.2 | 0.917 | 0.604 – 1.391 | 0.683 |
| | | 65-74 years | 26; 70 | 27.1 | 0.752 | 0.447 – 1.265 | 0.283 |
| | | ≥ 75 years | 6; 8 | 42.9 | 1.518 | 0.510 – 4.516 | 0.453 |
| | Owner gender (*n* = 917) | Female | 276; 555 | 33.2 | | Ref | |
| | | Male | 27; 59 | 31.4 | 0.920 | 0.571 – 1.484 | 0.733 |
| | Child ≤ 16 years in household (in 2020; *n* = 917) | No | 231; 437 | 34.6 | | Ref | |
| | | Yes | 73; 176 | 29.3 | 0.785 | 0.572 – 1.076 | 0.133 |
| | Employed in animal care/veterinary sector (*n* = 918) | No | 284; 570 | 33.3 | | Ref | |
| | | Yes | 20; 44 | 31.3 | 0.912 | 0.528 – 1.577 | 0.742 |
| | Another dog(s) in household (in 2020) (*n* = 908) | No | 247; 417 | 37.2 | | Ref | |
| | | **Yes** | **56; 188** | **23.0** | **0.503** | **0.359 – 0.705** | **< 0.001** |
| | Current working location of owner (*n* = 870) | At home | 83; 118 | 41.3 | 1.383 | 0.947 – 2.020 | 0.093 |
| | | Away from home | 47; 136 | 25.7 | 0.680 | 0.448 – 1.032 | 0.070 |
| | | At and away from home | 90; 177 | 33.7 | | Ref | |
| | | Unemployed | 11; 18 | 37.9 | 1.202 | 0.544 – 2.653 | 0.649 |
| | | Retired | 59; 131 | 31.1 | 0.886 | 0.595 – 1.319 | 0.551 |

**Table S7.** **Contd…**

| | Variable | Category | ***n* (harder; easier/as)** | **% (harder)** | **Odds Ratio** | **95% CI*** | ***p*-Value** |
|---|---|---|---|---|---|---|---|
| **Owner/ household demographics** | Current working location of other household members (*n* = 760) | At home | 44; 103 | 29.9 | 0.838 | 0.536 – 1.310 | 0.438 |
| | | Away from home | 85; 163 | 34.3 | 1.023 | 0.700 – 1.495 | 0.908 |
| | | At and away from home | 77; 151 | 33.8 | | Ref | |
| | | Unemployed | 6; 4 | 60.0 | 2.942 | 0.806 – 10.735 | 0.102 |
| | | Retired | 37; 90 | 29.1 | 0.806 | 0.503 – 1.291 | 0.370 |
| | Owner lives alone/only adult in the household (*n* = 871) | No | 247; 506 | 32.8 | | Ref | |
| | | Yes | 43; 75 | 36.4 | 1.175 | 0.784 – 1.760 | 0.436 |
| **Dog demographics** | Dog sex (*n* = 917) | Female | 118; 314 | 27.3 | | Ref | |
| | | **Male** | **186; 299** | **38.4** | **1.655** | **1.251 – 2.190** | **< 0.001** |
| | Neutered (*n* = 918) | No | 126; 256 | 33.0 | | Ref | |
| | | Yes | 178; 358 | 33.2 | 1.010 | 0.764 – 1.335 | 0.943 |
| | Breed purity designation (*n* = 918) | Crossbred | 12; 26 | 31.6 | | Ref | |
| | | Purebred | 214; 418 | 33.9 | 1.109 | 0.549 – 2.242 | 0.773 |
| | | Designer Crossbred | 78; 170 | 31.5 | 0.994 | 0.477 – 2.072 | 0.987 |
| | Top 12 most common breeds at 21 months (*n* = 918) | Crossbred | 12; 26 | 31.6 | | Ref | |
| | | Cockapoo | 35; 53 | 39.8 | 1.432 | 0.639 – 3.204 | 0.384 |
| | | Labradoodle | 10; 18 | 35.7 | 1.204 | 0.429 – 3.379 | 0.725 |
| | | Cavapoo | 5; 13 | 27.8 | 0.833 | 0.242 – 2.873 | 0.773 |
| | | Labrador Retriever | 31; 67 | 31.6 | 1.002 | 0.448 – 2.244 | 0.995 |
| | | Cocker Spaniel | 21; 42 | 33.3 | 1.083 | 0.458 – 2.564 | 0.856 |
| | | Border Collie | 11; 21 | 34.4 | 1.135 | 0.418 – 3.085 | 0.804 |
| | | Miniature Smooth-Haired Dachshund | 11; 18 | 37.9 | 1.324 | 0.480 – 3.654 | 0.588 |
| | | Border Terrier | 8; 18 | 30.8 | 0.963 | 0.328 – 2.829 | 0.945 |
| | | Golden Retriever | 12; 16 | 42.9 | 1.625 | 0.590 – 4.479 | 0.348 |
| | | Whippet | 6; 16 | 27.3 | 0.813 | 0.254 – 2.595 | 0.726 |
| | | English Springer Spaniel | 7; 13 | 35.0 | 1.167 | 0.371 – 3.668 | 0.792 |
| | | Other Purebred | 135; 293 | 31.5 | 0.998 | 0.489 – 2.038 | 0.996 |

**Table S7.** **Contd…**

| | Variable | Category | *n* (harder; easier/as) | % (harder) | Odds Ratio | 95% CI* | *p*-Value |
|---|---|---|---|---|---|---|---|
| **Dog demographics** | Breed group (*n* = 918) | Not KC recognised | 94; 200 | 32.0 | | Ref | |
| | | Gundog | 91; 178 | 33.8 | 1.088 | 0.765 – 1.547 | 0.640 |
| | | Hound | 28; 60 | 31.8 | 0.993 | 0.596 – 1.655 | 0.978 |
| | | Pastoral | 22; 42 | 34.4 | 1.114 | 0.630 – 1.973 | 0.710 |
| | | Terrier | 26; 55 | 32.1 | 1.006 | 0.594 – 1.704 | 0.983 |
| | | Toy | 8; 17 | 32.0 | 1.001 | 0.417 – 2.403 | 0.998 |
| | | Utility | 24; 39 | 38.1 | 1.309 | 0.745 – 2.302 | 0.349 |
| | | Working | 11; 23 | 32.4 | 1.018 | 0.476 – 2.174 | 0.964 |
| | Typical adult bodyweight (*n* = 883) | ≤ 10 kg | 61; 117 | 34.3 | 1.131 | 0.772 – 1.657 | 0.527 |
| | | 10 to < 20 kg | 112; 243 | 31.5 | | Ref | |
| | | 20 to < 30 kg | 67; 137 | 32.8 | 1.061 | 0.734 – 1.533 | 0.752 |
| | | 30 to < 40 kg | 50; 80 | 38.5 | 1.356 | 0.893 – 2.060 | 0.154 |
| | | ≥ 40 kg | 7; 9 | 43.8 | 1.687 | 0.613 – 4.646 | 0.311 |
| **Purchase motivations for a dog/for breed chosen** | Dog to improve mental health (*n* = 918) | No | 133; 326 | 29.0 | | Ref | |
| | | **Yes** | **171; 62.7** | **37.3** | **1.455** | **1.104 – 1.919** | **0.008** |
| | Breed size suited to my lifestyle (*n* = 918) | No | 100; 189 | 34.6 | | Ref | |
| | | Yes | 204; 425 | 32.4 | 0.907 | 0.676 – 1.218 | 0.517 |
| | Breed good with children (*n* = 918) | No | 175; 328 | 34.8 | | Ref | |
| | | Yes | 129; 286 | 31.1 | 0.845 | 0.641 – 1.116 | 0.235 |
| | Breed easy to train (*n* = 918) | No | 162; 320 | 33.6 | | Ref | |
| | | Yes | 142; 294 | 32.6 | 0.954 | 0.724 – 1.257 | 0.738 |
| | Friends or family own this breed/crossbreed (*n* = 918) | No | 231; 470 | 33.0 | | Ref | |
| | | Yes | 73; 144 | 33.6 | 1.031 | 0.747 – 1.424 | 0.851 |
| | Respondent driving force to get a dog (*n* = 900) | No | 167; 361 | 31.6 | | Ref | |
| | | Yes | 132; 240 | 35.5 | 1.189 | 0.898 – 1.574 | 0.227 |
| | Had more time for a dog due to COVID-19 pandemic (*n* = 377) | No | 15; 36 | 29.4 | | Ref | |
| | | Yes | 133; 193 | 40.8 | 1.654 | 0.871 – 3.141 | 0.124 |
| **Pre-purchase research** | Research conducted (*n* = 916) | No | 96; 286 | 25.1 | | Ref | |
| | | **Yes** | **207; 327** | **38.8** | **1.886** | **1.412 – 2.519** | **< 0.001** |
| **Purchase behaviours** | Via animal-specific selling website (*n* = 917) | No | 145; 305 | 32.2 | | Ref | |
| | | Yes | 158; 309 | 33.8 | 1.076 | 0.817 – 1.417 | 0.604 |
| | Viewed in person prior to purchase (*n* = 918) | No | 95; 184 | 34.1 | | Ref | |
| | | Yes | 209; 430 | 32.7 | 0.941 | 0.699 – 1.268 | 0.691 |

**Table S7.** **Contd…**

| | Variable | Category | *n* (harder; easier/as) | % (harder) | Odds Ratio | 95% CI* | *p*-Value |
|---|---|---|---|---|---|---|---|
| **Potentially illegal sales indicators** | Sold without dam present (*n* = 916) | No | 230; 491 | 31.9 | | Ref | |
| | | Yes | 74; 121 | 37.9 | 1.306 | 0.940 – 1.814 | 0.112 |
| | Sold outside breeders' home (*n* = 918) | No | 185; 392 | 32.1 | | Ref | |
| | | Yes | 119; 222 | 34.9 | 1.136 | 0.856 – 1.508 | 0.378 |
| | Sold without a microchip (*n* = 901) | No | 279; 580 | 32.5 | | Ref | |
| | | Yes | 19; 23 | 45.2 | 1.717 | 0.920 – 3.206 | 0.090 |
| | Sold < 6 weeks of age (*n* = 917) | No | 304; 611 | 33.2 | | Ref | |
| | | Yes | 0; 2 | 0.0 | ~ | ~ | ~ |
| | Sold with passport < 13 weeks of age (*n* = 833) | No | 266; 520 | 33.8 | | Ref | |
| | | Yes | 11; 36 | 23.4 | 0.597 | 0.299 – 1.192 | 0.144 |
| **Socialisation < 16 weeks** | Attended puppy classes (*n* = 917) | No | 159; 369 | 30.1 | | Ref | |
| | | **Yes, in person** | **119; 197** | **37.3** | **1.402** | **1.045 – 1.881** | **0.024** |
| | | Yes, online | 26; 47 | 35.6 | 1.284 | 0.768 – 2.146 | 0.341 |
| **Adult management** | Attended adult classes (*n* = 918) | No | 128; 379 | 25.2 | | Ref | |
| | | **Yes, in person** | **159; 216** | **42.4** | **2.180** | **1.637 – 2.903** | **< 0.001** |
| | | **Yes, online** | **17; 19** | **47.2** | **2.649** | **1.336 – 5.252** | **0.005** |
| | Owner training method (*n* = 889) | Rewards-based only | 38; 118 | 24.4 | | Ref | |
| | | Rewards-based & 1 aversive | 56; 132 | 29.8 | 1.317 | 0.814 – 2.131 | 0.261 |
| | | **Rewards-based & > 1 aversive** | **206; 339** | **37.8** | **1.887** | **1.259 – 2.828** | **0.002** |
| | | Aversive only | 0; 0 | 0.0 | ~ | ~ | ~ |
| | Exercise frequency (walks) (*n* = 882) | Never | 0; 1 | 0.0 | ~ | ~ | ~ |
| | | < Once a week | 1; 2 | 33.3 | 0.926 | 0.083 – 10.296 | 0.950 |
| | | Once a week | 1; 2 | 33.3 | 0.926 | 0.083 – 10.296 | 0.950 |
| | | Several times per week | 17; 30 | 36.2 | 1.049 | 0.559 – 1.968 | 0.882 |
| | | Once per day | 75; 160 | 31.9 | 0.868 | 0.616 – 1.222 | 0.416 |
| | | Twice per day | 141; 261 | 35.1 | | Ref | |
| | | > Twice per day | 57; 134 | 29.8 | 0.787 | 0.543 – 1.142 | 0.207 |
| | Exercise frequency (off lead) (*n* = 880) | **Never** | **26; 24** | **52.0** | **1.852** | **1.009 – 3.400** | **0.047** |
| | | **< Once a week** | **5; 26** | **16.1** | **0.329** | **0.122 – 0.884** | **0.027** |
| | | Once a week | 12; 15 | 44.4 | 1.368 | 0.616 – 3.039 | 0.442 |
| | | Several times per week | 53; 91 | 36.8 | 0.996 | 0.655 – 1.514 | 0.985 |
| | | Once per day | 100; 171 | 36.9 | | Ref | |
| | | Twice per day | 64; 160 | 28.6 | 0.684 | 0.467 – 1.001 | 0.051 |
| | | **> Twice per day** | **31; 102** | **23.3** | **0.520** | **0.324 – 0.833** | **0.007** |

**Table S7. Contd…**

| | Variable | Category | *n* (harder; easier/as) | % (harder) | Odds Ratio | 95% CI* | *p*-Value |
|---|---|---|---|---|---|---|---|
| **Adult management** | Time left alone (*n* = 892) | Never | 43; 62 | 41.0 | | Ref | |
| | | Up to 1 hour | 60; 999 | 37.7 | 0.874 | 0.528 – 1.447 | 0.600 |
| | | **1 to 4 hours** | **150; 366** | **29.1** | **0.591** | **0.383 – 0.911** | **0.017** |
| | | Over 4 hours | 41; 71 | 36.6 | 0.833 | 0.482 – 1.439 | 0.512 |
| | Change in time left alone from < 16 weeks (*n* = 862) | Same amount of time | 108; 251 | 30.1 | | Ref | |
| | | Less time | 11; 16 | 40.7 | 1.598 | 0.718 – 3.556 | 0.251 |
| | | More time ≤ 4 hours | 123; 251 | 32.9 | 1.139 | 0.834 – 1.556 | 0.414 |
| | | **More time > 4 hours** | **46; 56** | **45.1** | **1.909** | **1.217 – 2.996** | **0.005** |
| | Location left alone (*n* = 855) | **A crate/indoor kennel (door closed)** | **56; 77** | **42.1** | **1.581** | **1.065 – 2.346** | **0.023** |
| | | Loose in a single room | 74; 169 | 30.5 | 0.952 | 0.681 – 1.330 | 0.772 |
| | | Loose in multiple rooms | 150; 326 | 31.5 | | Ref | |
| | | Outdoor kennel (door closed) | 0; 1 | 0.0 | ~ | ~ | ~ |
| | | Outdoor kennel (door open) | 0; 1 | 0.0 | ~ | ~ | ~ |
| | | Loose in an enclosed outdoor space | 0; 1 | 0.0 | ~ | ~ | ~ |
| | General advice source for training and behaviour (*n* = 886) | Non-professional | 44; 219 | 16.7 | | Ref | |
| | | **Professional** | **252; 371** | **40.4** | **3.381** | **2.356 – 4.851** | **< 0.001** |
| | Respondent primary carer for dog (*n* = 917) | Shares care | 129; 269 | 32.4 | | Ref | |
| | | No | 2; 2 | 50.0 | 2.085 | 0.290 – 14.970 | 0.465 |
| | | Yes | 172; 343 | 33.4 | 1.046 | 0.791 – 1.382 | 0.753 |
| **Dog behaviour** | Total number of owner-reported undesirable behaviours (*n* = 784) | / / | / / | / / | **1.301** | **1.230 – 1.377** | **< 0.001** |
| | Control behaviour score (*n* = 876) | 0 | 23; 115 | 16.7 | | Ref | |
| | | **1+** | **269; 469** | **36.4** | **2.868** | **1.789 – 4.598** | **< 0.001** |
| | Attention seeking behaviour score (*n* = 834) | 0 | 30; 159 | 15.9 | | Ref | |
| | | **1+** | **248; 397** | **38.4** | **3.311** | **2.173 – 5.045** | **< 0.001** |
| | Aggressive behaviour score (*n* = 888) | 0 | 186; 480 | 27.9 | | Ref | |
| | | **1+** | **106; 116** | **47.7** | **2.358** | **1.724 – 3.225** | **< 0.001** |
| | Fear/avoidance behaviour score (*n* = 883) | 0 | 137; 379 | 26.6 | | Ref | |
| | | **1+** | **157; 210** | **42.8** | **2.068** | **1.556 – 2.749** | **< 0.001** |
| | Reaction to familiar people (*n* = 890) | 0 | 260; 578 | 31.0 | | Ref | |
| | | **1+** | **35; 17** | **67.3** | **4.577** | **2.518 – 8.320** | **< 0.001** |
| | Reaction to unfamiliar people (*n* = 894) | 0 | 215; 491 | 30.5 | | Ref | |
| | | **1+** | **80; 108** | **42.6** | **1.692** | **1.215 – 2.354** | **0.002** |

**Table S7. Contd…**

| | Variable | Category | *n* (harder; easier/as) | % (harder) | Odds Ratio | 95% CI* | *p*-Value |
|---|---|---|---|---|---|---|---|
| **Dog behaviour** | Reaction to other dogs (*n* = 891) | 0 | 197; 501 | 28.2 | | Ref | |
| | | **1+** | **98; 95** | **50.8** | **2.623** | **1.892 – 3.638** | **< 0.001** |
| | SRB case (*n* = 786) | No | 150; 392 | 27.7 | | Ref | |
| | | **Yes** | **99; 145** | **40.6** | **1.784** | **1.299 – 2.451** | **< 0.001** |
