# Supplementary material for: Impacts of Puppy Early Life Experiences, Puppy-Purchasing Practices, and Owner Characteristics on Owner-Reported Problem Behaviours in a UK Pandemic Puppies Cohort at 21 Months of Age
Source: Animals (Basel). 2024 Jan 22;14(2):336. doi: 10.3390/ani14020336 (PMC10812580; doi:10.3390/ani14020336)
Supplement: Supplementary file 1 [file animals-14-00336-s001.zip › File S3 Qualitative Coding Framework.pdf]

File S3: Qualitative Coding Framework for Content Analysis

The entire data set consisting of 1007 valid responses was subjected to content analysis at the same time.

In the case of question (Q's 27, 49 and 54), where only one response was requested, the first comment in the free-text was either assigned to a new category or back allocated to an existing category.

Back Allocating to Existing Deductive Category:
Free-text responses were allocated to the existing deductive categories (MCQ choices) if not selected by the respondent.

Miscellaneous free-text response:
Free-text responses which did not explicitly answer the question were classified as 'Miscellaneous' and were not included in the analysis.

Uninterpretable:
Free-text responses consisting of random digits or punctuation that didn't appear to mean anything were classified as 'uninterpretable' and not included in the analysis.

Creating New Inductive Categories:
Where comments did not correlate to the existing deductive categories (MCQ choices), new categories were created as described by Packer *et al*., Pandemic Puppies: Characterising motivations and behaviours of UK owners who purchased puppies during the 2020 COVID-19 Pandemic. *Animals*. **2021**, DOI 10.3390/ani11092500 (section 2.5. Qualitative Content Analysis of Free-Text Options).

The following tables give details of both the existing and new categories for the question analysed for this publication along with examples of comments.

Q3: If you are comfortable doing so, please describe in your own words why you decided to rehome, sell or give away or otherwise stop owning this dog?

- (*n* = 13)

Content Analysis Category	Example
I rehomed/sold/gave away this dog due to behavioural issues (owner-perceived behavioural issues) (*n* = 4)	"He was a sheep chaser and killed sheep. So we had to rehome him", "One of our older dogs was not happy with the 'pandemic pup' and they were fighting more and more. The pandemic pup then tried to attack a dog in the street and I decided then that it would be best to find her a home as an only dog. She also continually chased our cats."

Q8: If you are comfortable doing so, please describe in your own words what led to the decision for your dog to be put to sleep.

- (*n* = 9)

Content Analysis Category	Example(s)
My dog was put to sleep as a result of a behavioural issue (owner-perceived behavioural issues) (*n* = 3)	"He became very aggressive and killed a cat and attacked me", "Our dog had severe behaviour issues. He became reactive to both strangers and others dogs which lead to him becoming reactive to sounds outside of the house. He was extremely anxious all the time and began to attack myself and my partner randomly without triggers. He became extremely dangerous to be around and developed a bite history. We involved our vets and a behaviourist but unfortunately they were unable to help and agreed that behavioural euthinasia was our only option."

Q27: Is your dog insured?

- No, other (please describe below) (*n* = 14)

**Back Allocating to Existing Deductive Category**	**Example(s) Back Allocated**
Yes	"Thankfully - we are insured with Petplan they have paid our full entitlement of £7,000 within 7 days of submitting claim"
Yes – at the moment, but we are undecided whether to continue with it at the next renewal	NONE
No – and I do not plan to insure them	"Did a cost benefit analysis of insurance costs, my dog (a lurcher) and my savings and decided I would pay-as-I-go.", "Unlikely to insure."
No – but I plan to insure them in the future	NONE
No – they were insured but I have since cancelled or did not renew their policy	NONE
No – they came with 4 weeks free insurance as a puppy, but I did not continue with this	NONE
No – I have never heard of pet insurance	NONE

Q37: At any point in time have you or anyone in your household used any other aids or methods not listed previously on/with your dog to try and change any aspect of their behaviour?

- Yes (please describe below) (*n* = 140)

**Back Allocating to Existing Deductive Category**	**Example(s) Back Allocated**
Allowing sniffing time/interaction with the environment	NONE
Anti-bark 'Husher' muzzle	NONE
Bark-activated citronella/vibration/ultrasonic collar	"We used a spray collar that was activated by a button to stop her from eating things that she shouldn't …", 'Ultrasonic device'
Choke/check chain	NONE
Clicker training	"Marker word rather than clicker"
Electric collar	NONE
Electronic boundary fence	NONE
Food/treats	"Use of food …", "Food ball and snuffle mat"
Harness	NONE
Head collar	"Figure of eight lead.", "We use a dogmatic for walking on lead. He is a dreadful puller on lead and the dogmatic helps immensely."
Lead corrections (e.g., quickly yanking back on/jerking the lead if your dog pulls)	NONE
Pet Corrector™	NONE
Physical correction (e.g., smacking, tapping their nose, hitting, pinching)	NONE
Physical touch, e.g., stroking or patting	NONE
Physically moving your dog (e.g., pushing on your dog's hindquarters to get them into a sit, pushing them off furniture or if they jump up at you)	"…occassionally it has been necessary to raise our voice when he is over excited and also gently push/guide him away from jumping up us and visitors …"
Playing with other dogs	NONE
Playing with you/another household member	"using play as a reward"
Prong collar	NONE
Rattle bottle/cans/discs as a distractor	"Trainer recommended a soft rolled up towel to throw near them to shock them'"
Rubbing their nose in faeces/urine if they toilet in an inappropriate location	NONE
Shouting at them or telling them off	"Using different tone of voice", "…occassionally it has been necessary to raise our voice when he is over excited and also gently push/guide him away from jumping up us and visitors …"
"Time out": shutting your dog away within the home, away from people (e.g., in another room, in their crate)	NONE

Q37: Contd…

Back Allocating to Existing Deductive Category	Example(s) Back Allocated
"Time out": shutting your dog outdoors (e.g., in the garden/yard or an outside kennel)	NONE
Slip lead	NONE
Toys	"Squeaky ball", "We use his ball for recall/distraction"
Verbal praise	"Calmly talking to them", "Using different tone of voice", "Love and praise."
Water pistol/spray bottle	NONE

New Category	Example(s)
Chew deterrent (e.g., bitter apple spray, mustard, etc.) (n = 2)	"Bitter apple spray for chewing and mustard!", "Anti chew spray on furniture during teething"
Restricting access to a desired behaviour/resource until an appropriate behaviour is displayed (e.g., stopping when your dog pulls on the lead, ignoring them if they jump up, etc.) (n = 9)	"Lead walking - we stop when he is pulling on the lead and only carry on when he is calm", "Ignored when their behaviour wasn't desired but rewarded when it was I.e jumping up turning away but giving them praise when paws are on the floor"

Q39: At any point in time have you or anyone in your household used any other aids or methods not listed previously on/with your dog to try and change any aspect of their behaviour?

- (n = 131)

Back Allocating to Existing Deductive Category	Example(s) Back Allocated
Pulling on their lead	"…This leads to lead pulling, …"
Jumping up at people	"…Jumping up is a trait from his dad! …", "… It's just the friendly jumping only in the house never outside that I'm still working on"
Barking at other dogs	
Not coming back when called	"…He was dreadful always getting a scent and would not come back or excaping from the front door and calling him did not work.. …", "Recall when too close to other dogs, he wants to play with everyone."
Fear of loud sounds (e.g., fireworks, thunderstorms)	"Currently waiting for an appointment with a behaviourist due to an adverse reaction to fireworks resulting in him trigger stacking and it effecting his daily life", "Very occasional frightened by loud sounds eg fireworks if very close to her. Otherwise not worried"
Chasing (e.g., cats, wildlife, traffic)	NONE
Clinginess (e.g., following you, sitting close)	NONE
Anxiety/fear around other dogs	"A little nervous/submissive around other dogs, people", "He is very big and what appears to be aggression is really anxiety. This is getting better although very small dogs scare him rigid - the smaller the worse it is."
Anxiety/fear around people in your household (including you)	NONE
Anxiety/fear around unfamiliar people	"A little nervous/submissive around other dogs, people", "She can be very nervous with people - backing away from and barking at them if they approach her."
Aggression towards other dogs	"…aggression towards other intact male dogs …", "… recently has become a little aggressive to both dogs and humans …"
Aggression towards people in your household (including you)	NONE
Aggression towards unfamiliar people	"… recently has become a little aggressive to both dogs and humans ..."
Guarding of food, toys, or other items	NONE
Mouthing	**"Dog has been nipping me when excited when training"**
Barking or howling	"…he bowls / cries …", "Demand barking", "…barking, howling"
Being destructive	NONE
Toileting (weeing or pooing) indoors	"…Toileting indoors totally my fault. …"
Eating their or other dogs' faeces	"… still eats his own poo occasionly if I dont pick it up quickly enough.", "He does sometimes eat other dogs faeces when out in the field."
Mounting/humping other dogs, people or objects	"Humping me when over excited but no one else", "Oversexualised behaviour …"

Q39: Contd…

New Category	Example(s)
Anxiety/fear of unfamiliar situations (*n* = 21)	"Travel sickness and anxiety on long car journeys", "Being worried being inside buildings/ shops. He cowered and lay down …", "Fear of hot air balloons !"
Frustration/overstimulation in certain situations (*n* = 29)	"Being at a high arousal level, causing frustration and barking/howling in certain situations such as stopping on a walk.", "Barking with excitement when we get ready for a walk and barking at me when I stop to talk on a walk"
Problems sleeping overnight (*n* = 5)	"Barking early in morning around 5.30-6 am every day …", "My dog struggled to sleep overnight in new environments when visiting friends, despite crate training …"

Q46: Please describe here in your own words, any other sources of information not listed above that you have used for any aspect of your dog's general behaviour and training since the last survey (in November/December 2020).

- (*n* = 63)

Back Allocating to Existing Deductive Category	Example(s) Back Allocated
A veterinary professional (e.g., veterinary surgeon, veterinary nurse)	"Our daughters who is a degree qualified RVN"
Dog trainer	"We have attended at least 25 weekly dog training classes …"
Dog behaviourist	"1 : 1 with a dog behaviourist …"
My dog's breeder	"Breeder set up a What's App group to share experiences.", "Only contacted her breeder …"
Friends or family who own or had owned a dog	"Friends and acquaintances who are knowledgeable about dogs", "… we seek advice from friends and trusted sites e.g. RSPCA …"
The Kennel Club website	NONE
An animal charity website, e.g., Dogs Trust, RSPCA, PDSA, etc.	"… we seek advice from friends and trusted sites e.g. RSPCA …"
A breed/crossbreed-specific online resource (e.g., website/forum)	"Breed groups online now and again as this is our first time owning this breed of dog."
Social media sites, e.g., Facebook, Instagram	"Local Facebook groups for dogs/dog owners/parks in the area", "Pinterest"
YouTube	"We like watching YouTube videos …",
Book(s)	NONE
TV programmes	"We do watch dogs behaving badly …", "Dogs behaving very badly on TV is good", "The Ch 5 programme "Dogs Behaving Very Badly" is a good source of advice on generalised training approaches. (As well as being very entertaining!"
Dog-specific magazine(s)	NONE
My own experience from owning dogs in the past	"I am an experienced dog owner, …", "I am an experienced dog owner and most of the behaviours I feel confident in managing myself …"

New Category	Example(s)
My own experience as I am a canine professional (*n* = 6)	"Graduating vet student this year", "I am a veterinary behaviourist"
Another canine professional not listed above, e.g., dog walker, groomer, etc. (*n* = 5)	"And my dog walker", "Spoken to dog daycare staff"
An online training program/resource (e.g., APDT, IMDT, etc.) (*n* = 13)	"I use training advice from The School of Canine Science and IMDT, COAPE, APDT and Similar", "Online training courses (non-breed specific)"
A general online search (e.g., website/forum/internet search) (*n* = 4)	"online search", "Internet search/Google"

Q49: When your dog is left at home alone (without any people present in the home), which of the following spaces do they most often have access to?

- Other (please describe below) (*n* = 99)

**Back Allocating to Existing Deductive Category**	**Example(s) Back Allocated**
A crate/indoor kennel (with the door closed)	"During the day he is left in an extra large dog crate for a couple hours …", "… If she is unsupervised she has to be crated as she is destructive ..."
Loose in a single room, e.g., kitchen	"Loose in single room (bedroom) …", "She is left in a Kitchen with her crate open"
Loose in multiple rooms, e.g., hallway, living room, kitchen	"Free rein of the house with her crate open and available should she choose to go in it", "Loose in kitchen and hallway"
Outdoor kennel (with the door closed)	NONE
Outdoor kennel (with the door open)	"I sometimes leave her outside in a kennel and run weather permitting", "Outdoor double kennel and run with other dog in next kennel"
Loose in an enclosed outdoor space, e.g., garden, yard	NONE

Q53: When your dog has previously shown any of these behaviours when left at home alone, how have you responded soon after your return?

- Other (please describe below) (*n* = 31)

**Back Allocating to Existing Deductive Category**	**Example(s) Back Allocated**
No reaction, e.g., ignored what has happened	"Calm reaction from us upon arrival, once he had settled, then he will get praise and love."
Stroked/patted/cuddled them	"Calm reaction from us upon arrival, once he had settled, then he will get praise and love.", "…we cuddle and pat him on our return."
Played with them	"Let him out to play in the garden"
Told them off (e.g., shouted at them, said no)	"I have previously shouted and tapped his nose when he pooed inside after a long period of sucessful training …", "Say "who was that?!" and pointing at any damage ...", "Have said no and bad dog …"
Physically reprimanded them (e.g., tapped them, hit them, held them down)	"I have previously shouted and tapped his nose when he pooed inside after a long period of sucessful training …"
Put them in a 'time out' (e.g., on their own in a restricted indoor space, or outside)	"…Only if excessive do I resort to putting him in another room."

Q54: If you go out to work, do you take your dog to your place of work?

- No, other reasons (please describe below) (*n* = 97)

Back Allocating to Existing Deductive Category	Example(s) Back Allocated
Not applicable, I don't go out to work	"Do not work. Retired.", "Work from home"
Yes, always	NONE
Yes, most times (at least half or more of my workdays)	NONE
Yes, occasionally (less than half of my workdays)	NONE
No, I don't want to	NONE
No, my workplace is not suitable for dogs	"My partner could take him to work but he works in empty properties which isn't suitable for his allergies.", "Unfortunately it's generally not suitable for dogs …"
No, my workplace doesn't allow it	"Workplace doesn't allow …", "I am a teacher so I am unable to take her to work."
No, I have tried taking my dog to work previously but their behaviour was not suited to my work environment	"…she would not settle in the office and would be running about all day disturbing people and making it difficult to concentrate on work …"
No, I have not asked but may request to do this in the future	NONE
No, but another member of my household takes my dog to work with them	"My partner takes her to work very occasionally …", "Accompanies my partner to work …"

Q70: If you are comfortable doing so, please describe in your own words why you considered rehoming your dog.

- (*n* = 38)

Content Analysis Category	Example
I considered rehoming my dog due to its behaviour (*n* = 32)	"We are really struggling with her reactivity. We love her so much but we just want to be able to do "normal" activities with her and take her for a walk that doesn't feel high stress and high alert for triggers at all times.", "We can't leave him with our coming home to pee or poo", "Because he snapped at my daughter's school friend and was bossy around the house."

Q71: If you are comfortable doing so, please describe in your own words what you think would help/have helped you to avoid having to consider rehoming your dog. This could be advice pre- or post-purchase, access to resources, etc.

- (*n* = 31)

Content Analysis Category	Example
I/we would have improved my own knowledge pre-purchase (*n* = 7)	"I should have met more dogs of her breed. Spoken to owners. I would probably not chased this breed.", "Would not have purchased a border collie if I'd known how complex they can be"
I/we would have liked more practical support from canine professional's post-purchase (e.g., puppy training classes) (*n* = 15)	"Easier access to a behaviourist at reasonable cost", "…access to training classes at an earlier age (he had been booked in for training classes but these were cancelled due to lock-down)"
