# Supplementary material for: Impacts of Puppy Early Life Experiences, Puppy-Purchasing Practices, and Owner Characteristics on Owner-Reported Problem Behaviours in a UK Pandemic Puppies Cohort at 21 Months of Age
Source: Animals (Basel). 2024 Jan 22;14(2):336. doi: 10.3390/ani14020336 (PMC10812580; doi:10.3390/ani14020336)
Supplement: Supplementary file 1 [file animals-14-00336-s001.zip › File S4 Demographics.pdf]

File S4: Demographics

Comparison of 2020 owner demographics between the 21-month and full cohort

The majority of the respondents to the 21-month survey were female (21-month cohort: 90.2% vs. full cohort: 90.0%; $X^2 = 3.39$, $p = 0.183$) and between 45 – 54 years of age (21-month cohort: 27.7% vs. full cohort: 24.6%), with a lower proportion of owners aged between 18 – 44 years of age (21-month cohort: 42.1% vs. full cohort: 52.2%) and a higher proportion of older respondents aged 45 – ≥ 75 when compared to the full cohort (21-month cohort: 57.9% vs. full cohort: 47.8%; $X^2 = 38.09$, $p < 0.001$). In terms of the respondents to the 21-month survey who had answered the question regarding the age of children in the household in the original 2020 RVC Pandemic Puppies survey (21-month cohort: $n = 302$ vs. full cohort: $n = 1325$), 90.4% had indicated they had children under the age of 16 compared to 89.7% in the full cohort ($X^2 = 0.12$, $p = 0.731$). A total of 7.3% of the 21-month respondents had indicated in 2020 that they worked with animals in some capacity compared to 10.0% in the full cohort ($X^2 = 6.63$, $p = 0.010$). The proportion of respondents to the 21-month survey who had indicated they were first-time dog owners when purchasing their Pandemic Puppy was 38.6% compared to 40.4% in the full cohort ($X^2 = 1.02$, $p = 0.312$).

Table S8. Demographic characteristics of owners in 2020 in both the 21-month and full cohort.

Demographic characteristics of the *n* = 985 participants who responded to the 21 month survey and indicated they still owned their dog ('21 month cohort'). The characteristics were compared to those of all the *n* = 4369 respondents to the original 2020 RVC Pandemic Puppies survey ('full cohort'). Significant differences are emboldened.

Category (21-month, 2020)	Response	Cohort		Statistics
		**21-month %**	**2020 %**	
**Owner gender** (21-month: *n* = 984, 2020: *n* = 3788)	Male	9.8	9.6	$X^2 = 3.393$ $p = 0.183$
	Female	90.2	90.0	
	Other	0.0	0.3	
	I prefer not to say	0.0	0.0	
**Owner age** (21-month: *n* = 983, 2020: *n* = 3790)	18 – 24 years	4.3	5.6	**$X^2 = 38.094$ $p < 0.001$**
	25 – 34 years	19.1	24.1	
	35 – 44 years	18.7	22.5	
	45 – 54 years	27.7	24.6	
	55 – 64 years	18.7	15.6	
	65 – 74 years	10.1	6.7	
	75 years and over	1.4	0.9	
**Respondents with children who indicated they were ≤ 15 years of age** (21-month: *n* = 302, 2020: *n* = 1325)		90.4	89.7	$X^2 = 0.118$ $p = 0.731$
**Employed in animal care sector** (21-month: *n* = 985, 2020: *n* = 3789)	Yes	7.3	10.0	**$X^2 = 6.628$ $p = 0.010$**
	No	92.7	90.0	
	I'm not sure	0.0	0.0	
**First time dog owner** (21-month: *n* = 984, 2020: *n* = 3783)		38.6	40.4	$X^2 = 1.023$ $p = 0.312$

Comparison of 2020 dog demographics between the 21-month and full cohort

There was no significant difference between the sex distribution of dogs amongst the 21-month survey cohort and the full cohort (male, 21-month cohort: 52.7% vs. male, full cohort: 53.4%; X^2 = 0.12, p = 0.710) or the dogs age at acquisition (X^2 = 8.70, p = 0.069). In terms of neuter status there was no significant difference at the time of the original 2020 RVC Pandemic Puppies between the 21-month cohort and full cohort (19.3% vs. 19.5% respectively; X^2 = 0.02, p = 0.879). The proportion of dogs that were insured at the time of the original 2020 RVC Pandemic Puppies survey also did not differ significantly between the two cohorts (21-month cohort: 85.4% vs. full cohort: 84.0%; X^2 = 1.16, p = 0.282). There was no significant difference in the proportion of designer crossbred dogs between both cohorts (21-month cohort: 26.8% vs. full cohort: 26.1%; X^2 = 2.44, p = 0.295) or in the proportion of purebred puppies (21-month cohort: 68.5% vs. full cohort: 70.5%). There was no significant difference in the proportion of dogs registered with The Kennel Club between the 21-month cohort and the full cohort (21-month cohort: 45.8% vs. full cohort: 46.2%; X^2 = 2.97, p = 0.226).

Table S9. Demographic characteristics of owners in 2020 in both the 21-month and full cohort.

Demographic characteristics of the *n* = 985 dogs whose owners responded to the 21-month survey, indicating they still owned their dog ('21 month cohort'). The characteristics were compared to those of all the *n* = 4369 dogs from respondents to the original 2020 RVC Pandemic Puppies survey ('full cohort').

**Category (21-month, 2020)**	**Response**	**Cohort**		**Statistics**
		**21-month %**	**2020 %**	
**Dog age at acquisition** (21-month: *n* = 984, 2020: *n* = 4118)	13 – 16 weeks	1.0	2.4	$X^2 = 8.703$ $p = 0.069$
	11 – 12 weeks	4.7	5.5	
	9 – 10 weeks	24.7	24.5	
	7 – 8 weeks	69.4	67.3	
	Under 6 weeks	0.2	0.3	
	Can't remember	0.0	0.0	
**Dog gender** (21-month: *n* = 983, 2020: *n* = 4371)	Male	52.7	53.4	$X^2 = 0.139$ $p = 0.710$
	Female	47.3	46.6	
**Neutered** (21-month: *n* = 984, 2020: *n* = 4072)		19.3	19.5	$X^2 = 0.023$ $p = 0.879$
**Insured** (21-month: *n* = 983, 2020: *n* = 4732)		85.4	84.0	$X^2 = 1.158$ $p = 0.282$
**Designer crossbred, purebred or crossbred status** (21-month: *n* = 985, 2020: *n* = 4386)	Designer	26.8	26.1	$X^2 = 2.441$ $p = 0.295$
	Purebred	68.8	70.5	
	Crossbred	4.4	3.4	
**Kennel Club Registered** (21-month: *n* = 981, 2020: *n* = 4379)	Yes	45.8	46.2	$X^2 = 2.970$ $p = 0.226$
	No	52.7	51.5	
	I'm not sure	1.5	2.4	

The 12 most popular dog breeds/crossbreeds among the dogs owned by 21-month survey respondents are described in Table A10. All the top ten most frequently reported breeds/crossbreeds in the full cohort were represented within the top 12 most frequently reported breeds/crossbreeds in the 21-month cohort. Additionally, there was a significant increase in the proportion of Whippets in the 21-month cohort (21-month cohort: ranked 10th vs. full cohort: ranked 14th; $X^2 = 4.27$, $p = 0.039$).

Table S10. Most popular individual breeds/crossbreeds in the 21-month (*n* = 985) and full (*n* = 4358) cohorts in 2020.

Crossbred dogs were defined as mixed breeds of unknown origin, or crosses who were not reported using a breed-indicative designer crossbreed name, e.g., Spaniel Cross. The breeds with the greatest frequency, which formed the top twelve most popular breeds for the 21-month cohort, are shown along with their ranking (1 = most popular) for each cohort. Significant differences are emboldened.

**Breed/Crossbreed**	**21-month cohort**			**Full cohort**			**Statistics**	
	n	**%**	**Rank**	***n***	**%**	**Rank**	**X^2**	***p* Value**
Labrador Retriever	103	10.5	1	429	9.9	1	0.34	0.562
Cockapoo	90	9.1	2	362	8.3	2	0.72	0.398
Cocker Spaniel	70	7.1	3	324	7.4	3	0.13	0.722
Crossbreed	43	4.4	4	149	3.5	6	2.08	0.149
Border Collie	33	3.4	5	150	3.4	5	0.02	0.886
Miniature Smooth-Haired Dachshund	31	3.1	6	188	4.4	4	2.78	0.095
Border Terrier	30	3.0	7	140	3.2	7	0.07	0.788
Labradoodle	29	2.9	=8	127	2.9	8	0.00	0.960
Golden Retriever	29	2.9	=8	120	2.8	9	0.11	0.743
**Whippet**	**23**	**2.3**	**10**	**62**	**1.4**	**14**	**4.27**	**0.039**
English Springer Spaniel	20	2.0	11	96	2.2	11	0.11	0.737
Cavapoo	19	1.9	12	111	2.6	10	1.29	0.255

Spatial analysis of respondents

The spatial distribution of 21-month survey respondents is described in Table A11., with no significant differences seen between the two cohorts (X^2 = 4.12, p = 0.967).

Table S11. Spatial distribution of respondents in 2020 in the 21-month (*n* = 962) and full (*n* = 3507) cohorts. Respondents' partial postcode data was used to assess the representativeness of the study sample compared to the UK population. As described previously, the full cohort showed no significant difference in geographical distribution compared with mid-2020 Office for National Statistics population data (Packer *et al.*, 2021).

**UK Region (21-month: *n* = 962, 2020: *n* = 3507)**	**Cohort**		**Statistics**
	**21-month %**	**2020 %**	
East Midlands	6.2	6.7	X^2 = 4.107 p = 0.967
East of England	13.6	13.5	
London	12.2	10.8	
North East	4.0	4.5	
North West	8.3	8.1	
Northern Ireland	0.5	0.7	
Scotland	8.2	9.2	
South East	15.6	16.1	
South West	11.7	10.9	
Wales	4.5	4.2	
West Midlands	7.2	7.5	
Yorkshire and The Humber	8.0	7.8	
